# Identification of orphan histidine kinases that impact sporulation and enterotoxin production by *Clostridium perfringens* type F strain SM101 in a pathophysiologically-relevant *ex vivo* mouse intestinal contents model

**DOI:** 10.1371/journal.ppat.1011429

**Published:** 2023-06-01

**Authors:** Iman Mehdizadeh Gohari, Jihong Li, Mauricio A. Navarro, Fábio S. Mendonça, Francisco A. Uzal, Bruce A. McClane

**Affiliations:** 1 Department of Microbiology and Molecular Genetics, University of Pittsburgh School of Medicine, Pittsburgh, Pennsylvania, United States of America; 2 California Animal Health and Food Safety Laboratory System, School of Veterinary Medicine, University of California Davis, San Bernardino, California, United States of America; Boston Children’s Hospital, UNITED STATES

## Abstract

When causing food poisoning or antibiotic-associated diarrhea, *Clostridium perfringens* type F strains must sporulate to produce *C*. *perfringens* enterotoxin (CPE) in the intestines. *C*. *perfringens* is thought to use some of its seven annotated orphan histidine kinases to phosphorylate Spo0A and initiate sporulation and CPE production. We previously demonstrated the CPR0195 orphan kinase, but not the putative CPR1055 orphan kinase, is important when type F strain SM101 initiates sporulation and CPE production in modified Duncan-Strong (MDS) sporulation medium. Since there is no small animal model for *C*. *perfringens* sporulation, the current study used diluted mouse intestinal contents (MIC) to develop an *ex vivo* sporulation model and employed this model to test sporulation and CPE production by SM101 CPR0195 and CPR1055 null mutants in a pathophysiologically-relevant context. Surprisingly, both mutants still sporulated and produced CPE at wild-type levels in MIC. Therefore, five single null mutants were constructed that cannot produce one of the previously-unstudied putative orphan kinases of SM101. Those mutants implicated CPR1316, CPR1493, CPR1953 and CPR1954 in sporulation and CPE production by SM101 MDS cultures. Phosphorylation activity was necessary for CPR1316, CPR1493, CPR1953 and CPR1954 to affect sporulation in those MDS cultures, supporting their identity as kinases. Importantly, only the CPR1953 or CPR1954 null mutants exhibited significantly reduced levels of sporulation and CPE production in MIC cultures. These phenotypes were reversible by complementation. Characterization studies suggested that, in MDS or MIC, the CPR1953 and CPR1954 mutants produce less Spo0A than wild-type SM101. In addition, the CPR1954 mutant exhibited little or no Spo0A phosphorylation in MDS cultures. These studies, i) highlight the importance of using pathophysiologically-relevant models to investigate *C*. *perfringens* sporulation and CPE production in a disease context and ii) link the CPR1953 and CPR1954 kinases to *C*. *perfringens* sporulation and CPE production in disease-relevant conditions.

## Author summary

*Clostridium perfringens* type F strains are a major cause of several human gastrointestinal diseases, including food poisoning. Their virulence requires the *in vivo* production of *C*. *perfringens* enterotoxin (CPE). Sporulation also plays a crucial role in the pathogenesis of type F-associated infections since spores contribute to disease transmission and CPE is only produced during sporulation. Because regulation and initiation of sporulation in *C*. *perfringens* is incompletely understood, our study evaluated the role of annotated orphan histidine kinases in regulating *C*. *perfringens* sporulation and CPE production using a newly-developed *ex vivo* mouse intestinal contents (MIC) model, as well as modified Duncan-Strong (MDS) laboratory sporulation medium. This work identified two orphan histidine kinases that play a critical role in regulating *C*. *perfringens* sporulation and CPE toxin production in both MIC and MDS. It was also determined that, at natural expression levels, those two kinases (named CPR1953 and CPR1954) regulate Spo0A production, with CPR1954 also modulating Spo0A phosphorylation, the key initial step in sporulation.

## Introduction

The anaerobic, Gram-positive, spore-former *Clostridium perfringens* is a major pathogen of humans and other animals [1–3]. This bacterium is a proficient toxin-producer capable of making more than 20 different toxins. However, toxin production is highly variable among strains, allowing classification of *C*. *perfringens* isolates into seven toxinotypes (A-G) based upon their carriage of genes encoding six toxins named alpha toxin (which is encoded by all toxin types), beta toxin, enterotoxin (CPE), epsilon toxin, iota toxin, and NetB toxin [4].

By definition, *C*. *perfringens* type F strains must carry the genes encoding CPE and alpha toxin, but not the other four typing toxins [4]. CPE production is required for the virulence of type F strains in relevant animal models [5]. Type F strains are of major importance for causing several human gastrointestinal (GI) diseases. Specifically, these bacteria cause *C*. *perfringens* type F food poisoning, which is the 2^nd^ most common bacterial foodborne diseases in the USA, where about 1 million cases/year occur [6]. Type F strains are also responsible for causing ~5–10% of all cases of nonfoodborne gastrointestinal diseases, such as sporadic diarrhea or antibiotic-associated diarrhea [2,7].

Like most clostridia, *C*. *perfringens* forms spores to survive in adverse conditions. Spores also contribute to *C*. *perfringens* pathogenesis by facilitating transmission of this bacterium from the environment to the host. Sporulation plays a particularly critical role in type F-associated diseases. Food poisoning initiates when type F strains, often in the spore form, contaminate food [6]. Compared to the spores of other *C*. *perfringens* strains, spores made by type F food poisoning strains are typically more resistant to food environment stresses such as heat, cold or preservatives, often because they produce a α/β-type small acid soluble protein-4 variant [8]. In improperly prepared/stored foods, those spores germinate into vegetative cells, which then rapidly multiply. Once ingested in contaminated food, some type F vegetative cells are killed while transiting the stomach but survivors multiply, and later sporulate, in the intestinal lumen [6]. During this intestinal sporulation, CPE is produced [6]. When type F strains cause nonfoodborne GI diseases, spores are ingested from the health care-associated environment and, after intestinal spore germination and vegetative cell multiplication, *in vivo* sporulation then results in CPE production [6,9].

The processes of *C*. *perfringens* sporulation and sporulation-associated CPE production are only partially understood. Sporulation initiation for this bacterium requires the master regulator Spo0A [10]. Once phosphorylated, Spo0A is thought to cause transcriptional changes that induce a sporulation cascade involving sporulation-associated sigma factors named SigE, SigF, SigG, and SigK. In type F strains, SigF is produced early and affects production of the other three sigma factors [11]. All four sporulation-associated sigma factors are required for *C*. *perfringens* sporulation [11,12]. However, the sporulation-dependent nature of CPE production by type F strains only requires SigF, SigE and SigK [11,12]. SigF controls production of both SigE and SigK [11], which then direct RNA polymerase to recognize SigE- and SigK-dependent promoters located upstream of the *cpe* ORF and thus drive *cpe* gene expression [13].

A remaining gap in understanding *C*. *perfringens* sporulation concerns the steps leading to Spo0A phosphorylation, which is believed to initiate sporulation and CPE production. In *Bacillus subtilis*, the paradigm species for understanding sporulation by Gram-positive spore-forming bacteria, sporulation begins when Spo0A is phosphorylated via a phosphorelay mechanism triggered by several histidine kinases in the presence of appropriate environmental signals such as starvation or population density [14–16]. Like other pathogenic clostridia, *C*. *perfringens* lacks a phosphorelay, so it has been proposed that orphan histidine kinases direct the phosphorylation of Spo0A to initiate *C*. *perfringens* sporulation and, later, CPE production [17].

Previous bioinformatic genome analysis [17] of SM101 (a transformable derivative of a type F food poisoning strain) identified seven putative orphan histidine kinase genes, i.e. *cpr0195*, *cpr1055*, *cpr1316*, *cpr1493*, *cpr1728*, *cpr1953*, and *cpr1954*. That previous study also reported orphan histidine kinase CPR0195, but not the putative CPR1055 orphan kinase, is important for efficient sporulation and CPE production when SM101 is cultured in modified Duncan-Strong (MDS) sporulation medium [17]. Confirming its identity as a kinase, CPR0195 was shown to phosphorylate Spo0A *in vitro* [17].

Notably, all previous studies of sporulation and CPE production by type F strains used laboratory sporulation media, such as MDS, with questionable pathophysiologic relevance. One approach to improving understanding of type F strain intestinal sporulation and CPE production under more pathophysiologically-relevant conditions would be to utilize a small animal intestinal challenge model. Unfortunately, developing such a model has not been successful to date, e.g., no spores were recovered after oral challenge of mice with type F strain F4969 [18]. This difficulty may reflect type F strain sporulation and CPE production/release being a slow (> 8 h) process [6], while the intestinal transit time for small animals such as mice is only ~6 h, i.e., a type F bacterial challenge may be largely excreted from mouse intestines before sporulation or CPE production and release can be completed.

Consequently, the current study first sought to develop an *ex vivo* diluted mouse intestine luminal contents (referred to as MIC) model to study the regulation of sporulation and CPE production in an environment resembling the intestinal lumen where type F disease occurs. This MIC model was then used to test whether the CPR0195 kinase also regulates sporulation and CPE production under more pathophysiologically-relevant conditions. Last, this MIC model and MDS laboratory sporulation medium were employed to evaluate the role of the five annotated, but previously-unstudied, *C*. *perfringens* putative orphan histidine kinases in controlling sporulation and CPE production in MDS and MIC.

## Results

### Development of a mouse diluted small intestine luminal contents *ex vivo* model for studying type F strain sporulation and CPE production

In the absence of a small animal type F intestinal challenge model, the current study first sought to develop a more pathophysiologically-relevant *ex vivo* model than laboratory sporulation media, such as MDS, to study *C*. *perfringens* type F strain sporulation and CPE production. For this purpose, viable vegetative cell numbers, sporulation and CPE production were evaluated for two type F strains, i.e., SM101 (a transformable derivative of a food poisoning strain carrying a chromosomal *cpe* gene [13]) and F4969 (a sporadic diarrhea strain carrying a plasmid *cpe* gene [19]), when cultured *ex vivo* in sterile PBS-diluted mouse intestinal contents, here after referred to as MIC, or PBS without mouse intestinal contents. Since *C*. *perfringens* is anaerobic, MIC and PBS were supplemented with Oxyrase to create a more reduced environment, which significantly enhanced vegetative cell survival and sporulation in both culture conditions (S1 Fig). Note that laboratory sporulation media also typically contain a reducing agent, e.g., MDS is supplemented with sodium thioglycolate.

**Fig 1:**
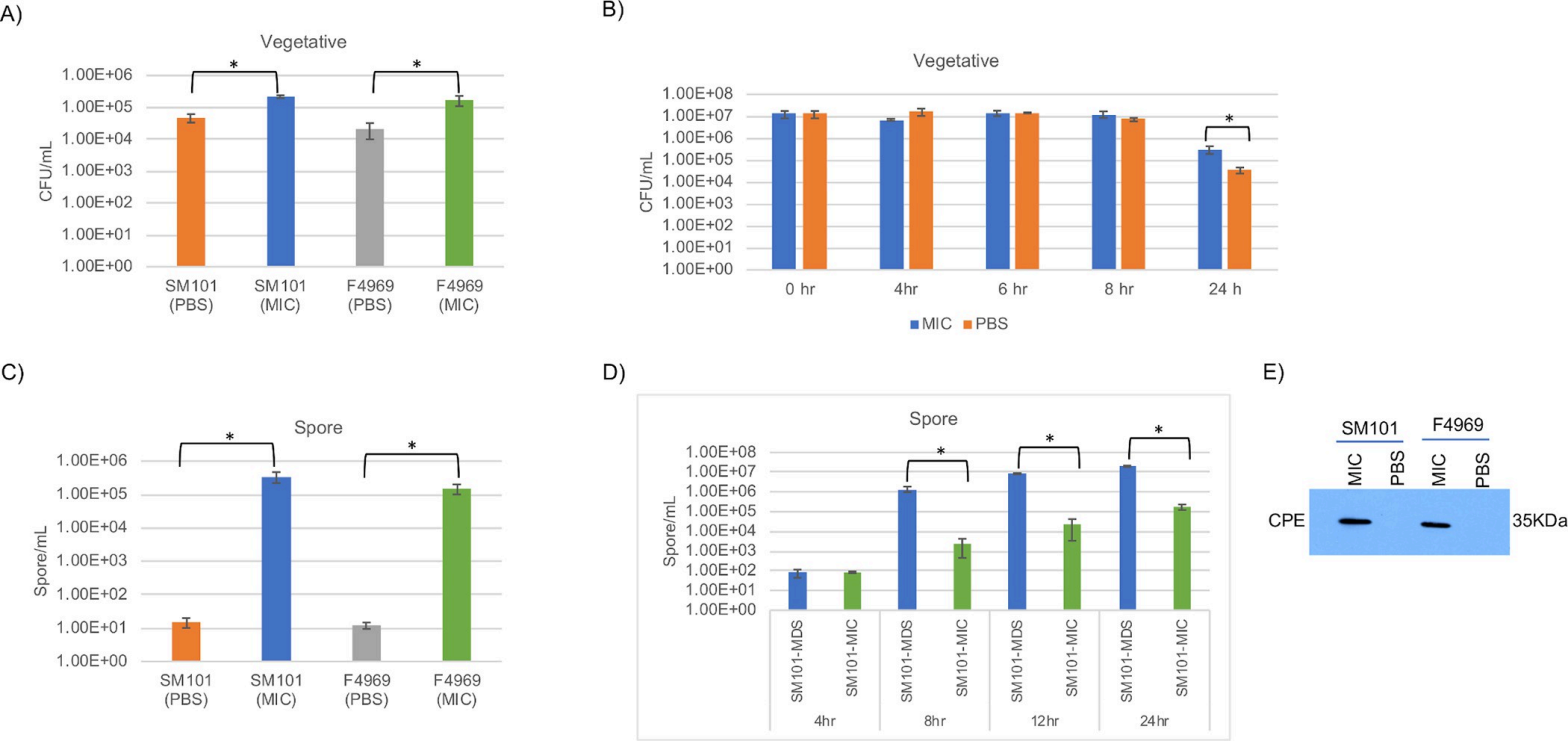
Development of an *ex vivo* model for studying type F strain sporulation and CPE toxin production. (A) Viable vegetative cells (CFU/mL) for SM101 or F4969 cultured overnight at 37°C in PBS or sterile PBS-diluted mice intestinal contents (MIC), each containing 5% Oxyrase. (B) Viable vegetative cells (CFU/mL) for SM101 grown at 37°C for the indicated times in MIC or PBS, each containing 5% Oxyrase. (C) Heat-resistant spores (CFU/mL) for SM101 or F4969 grown overnight at 37°C in PBS or MIC, each containing 5% Oxyrase. (D) Heat-resistant spores (CFU/mL) for SM101 grown at 37°C for the indicated times in MDS or MIC containing 5% Oxyrase. (E) Western blot analysis of CPE levels in supernatants of SM101 and F4969 cultured overnight at 37°C in PBS or MIC containing 5% Oxyrase. The blot shown is representative of three independent experiments. A loading control for the Western blot experiment is presented in S2 Fig. Experiments shown in panels A, B, C, and D are presented as the mean ± SD of three independent experiments. Student’s unpaired *t* test was used for statistical analysis of matching MIC vs. PBS cultures in panels A, B, C, and for matching MDS vs. MIC cultures in panel D. Asterisk indicates *p* < 0.05.

In overnight (16–18 h) cultures supplemented with 5% Oxyrase, significantly more viable vegetative cells of SM101 or F4969 were present in MIC vs. PBS cultures (Fig 1A). A time-course study (Fig 1B) showed that the number of viable SM101 vegetative cells remained similar to the inoculum in those PBS and MIC cultures through at least the initial 8 h, but then dropped by 24 h. However, by the 24 h timepoint, the number of viable SM101 vegetative cells in MIC was slightly higher when cultured in MIC vs PBS.

When those overnight SM101 and F4969 MIC cultures supplemented with 5% Oxyrase were heat-shocked at 70°C for 20 min to select for heat-resistant spores, they both contained 10^5^ to 10^6^ heat-resistant spores per mL, whereas the overnight PBS cultures of those strains contained only negligible (~10 per mL) numbers of heat-resistant spores (Fig 1C). Since 5% Oxyrase was present in both the MIC and PBS cultures, these results indicate that MIC was the factor inducing sporulation in the MIC cultures.

A time-course study then compared the kinetics of heat-resistant spore formation by SM101 when cultured in MDS vs MIC (plus 5% Oxyrase). This analysis detected significant production of heat-resistant spores after ~8 h of incubation in either culture condition (Fig 1D). For MDS cultures, the number of heat-resistant spores then increased ~10-fold thereafter up to 24 h. In contrast, formation of heat-resistant spores in MIC cultures increased nearly 100-fold between 8 h and 24 h. Fig 1D results also revealed that, after a 24 incubation, the formation of heat-resistant spores was approximately 100-fold higher for SM101 cultured in MDS vs. MIC.

Western blot analysis (Fig 1E) detected CPE production by both type F strains when cultured overnight in MIC plus 5% Oxyrase, but not when cultured overnight in PBS containing 5% Oxyrase. Since CPE is only produced during sporulation, those Fig 1E CPE Western blot results are consistent with the Fig 1C results detecting significant sporulation by SM101 and F4969 cultured in MIC plus Oxyrase, but not PBS plus Oxyrase.

Collectively Fig 1 results indicated that MIC are a useful pathophysiologically-relevant *ex vivo* model to study *C*. *perfringens* sporulation and CPE production. Since Oxyrase significantly enhanced *C*. *perfringens* survival and sporulation, in MIC, and Oxyrase did not itself induce sporulation or CPE production, all further experiments using MIC were supplemented with 5% Oxyrase.

### Comparing the importance of CPR0195 and CPR1055 for vegetative cell viability, sporulation and CPE production when SM101 is cultured in MIC vs. MDS

Our previous work implicated the orphan histidine kinase CPR0195, but not the putative CPR1055 orphan histidine kinase, in regulating sporulation and CPE production when SM101 is cultured in MDS [17]. Therefore, the current study next compared the effects of inactivating *cpr0195* or *cpr1055* expression on vegetative cell viability, sporulation and CPE production when SM101 is cultured overnight in MIC vs. MDS.

**Fig 2:**
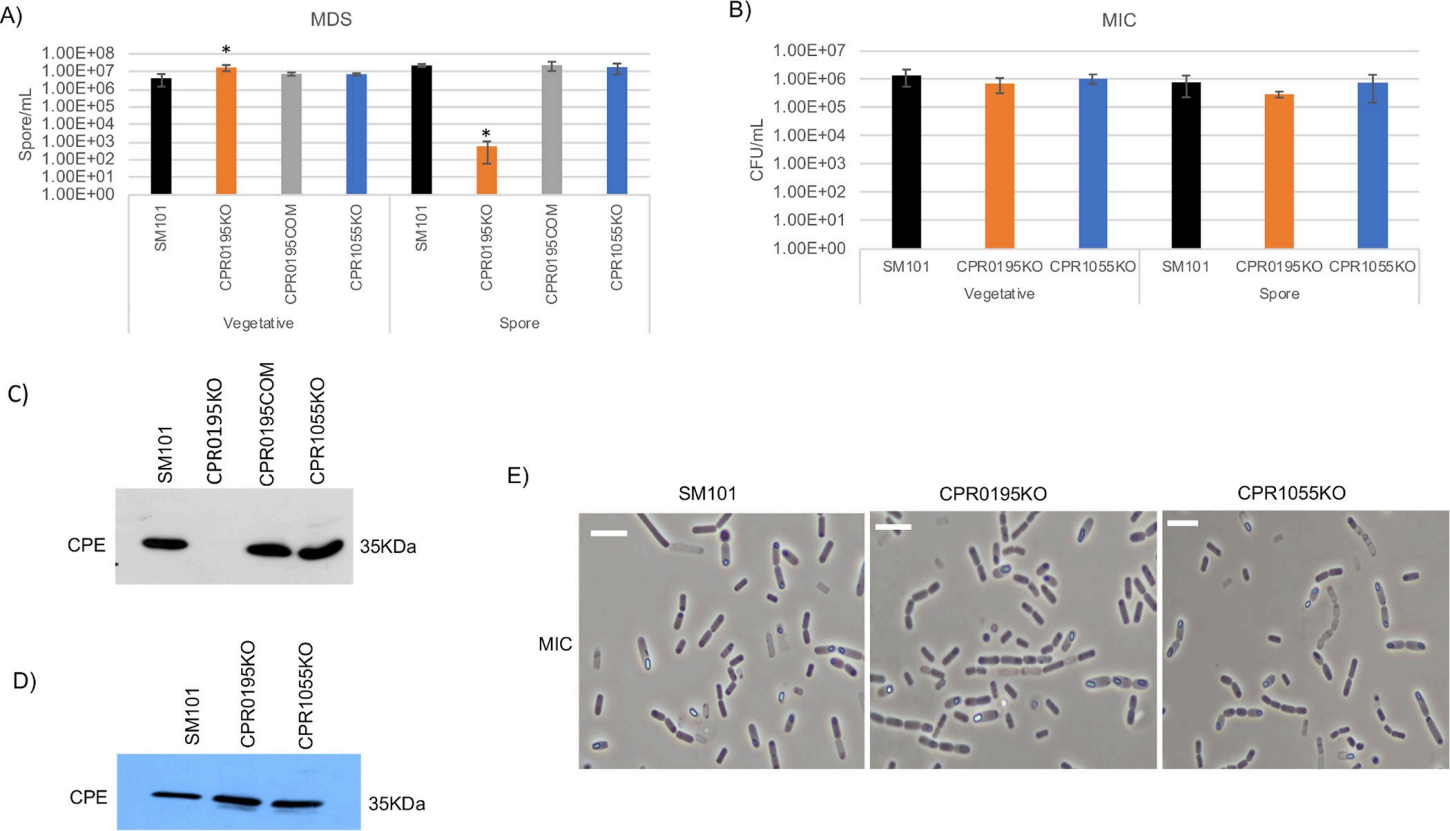
Comparison of the importance of CPR0195 and CPR1055 for viable vegetative cell numbers, sporulation and CPE production when SM101 is cultured in MDS vs. MIC. (A) “Vegetative”, viable vegetative cells (CFU/mL) for SM101, CPR0195KO, CPR0195COM, or CPR1055KO cultured overnight at 37°C in MDS. “Spores”, heat-resistant spores (CFU/mL) in aliquots of those same MDS cultures after 20 min treatment at 70°C to kill vegetative cells. (B) “Vegetative”, viable vegetative cells (CFU/mL) for SM101, CPR0195KO, or CPR1055KO cultured overnight at 37°C in MIC. “Spores”, heat-resistant spores (CFU/mL) in aliquots of those same MIC cultures. (C) Western blot analysis of CPE levels in supernatants from SM101, CPR0195KO, CPR0195COM, or CPR1055KO MDS cultures incubated overnight at 37°C. (D) Western blot analysis of CPE levels in supernatants from SM101, CPR0195KO, or CPR1055KO MIC cultures incubated overnight at 37°C in MIC. (E) Phase-contrast photomicroscopy of wild-type SM101, CPR0195KO, and CPR1055KO cultured in MIC. The white scale bar represents 10 μm. Oxyrase (5%) was present in MIC cultures used in panels B, D and E. Results for panels A and B are presented as the mean ± SD of three independent experiments. Ordinary one-way analysis of variance (ANOVA; GraphPad Prism 8) was used for statistical analysis of vegetative cell or spore numbers in panels A and B. Asterisk represents *p* < 0.05 versus SM101. Western blots in panels C and D are representative of three independent experiments. Loading controls for these Western blot experiments are presented in S2 Fig.

Overnight MDS cultures of the isogenic CPR0195KO null mutant contained slightly more viable vegetative cells than SM101 MDS culture (Fig 2A), while overnight MIC cultures of CPR0195KO mutant contained wild-type levels of viable vegetative cells (Fig 2B). Confirming our previous report [17], the CPR0195KO null mutant produced 10^4^-fold fewer heat-resistant spores than the wild-type SM101 parent when cultured overnight in MDS (Fig 2A) and this effect was reversible by complementation (Fig 2A). Surprisingly however, the CPR0195KO mutant still produced wild-type levels of heat-resistant spores when cultured overnight in MIC (Fig 2B).

Consistent with our previous report [17], the CPR1055KO null mutant produced wild-type numbers of viable vegetative cells and heat-resistant spores in overnight MDS cultures. This mutant also formed wild-type levels of viable vegetative cells and heat-resistant spores when cultured in MIC (Fig 2A and 2B).

Western blot analysis demonstrated CPE production by wild-type SM101 cultured overnight in either MDS or MIC (Fig 2C and 2D). As expected from our previous study [17], no detectable CPE was produced when the CPR0195KO mutant was cultured overnight in MDS, while complementation of this mutant restored CPE production under these culture conditions (Fig 2C). However, this mutant still produced wild-type CPE levels when cultured overnight in MIC (Fig 2D), which is consistent with the ability of this mutant to sporulate well in MIC (Fig 2B). As we reported previously [17], the CPR1055KO mutant still produced CPE at wild-type levels when cultured overnight in MDS (Fig 2C). The current study determined that CPR1055KO also produces wild-type CPE levels when cultured overnight in MIC (Fig 2D), consistent with the MIC sporulation results for this mutant (Fig 2B).

Our previous study [17] also reported the presence of numerous phase-refractile spores in ~18 h MDS cultures of CPR1055KO, but not CPR0195KO. In the current study, phase-contrast photomicroscopy (Fig 2E) detected the presence of phase-refractile spores in 18 h MIC cultures of CPR0195KO and CPR1055KO, consistent with the ability of both mutants to sporulate in MIC.

### Construction of additional isogenic orphan histidine kinase null mutant and complementing strains

Since Fig 2 results indicated that neither CPR0195 nor CPR1055 are necessary for SM101 sporulation or CPE production in *ex vivo* MIC culture conditions, contributions of the five unstudied putative orphan histidine kinases of *C*. *perfringens* to vegetative cell viability, sporulation or CPE production were evaluated for SM101 cultured in MDS or MIC. For these analyses, the *cpr1728*, *cpr1316*, *cpr1493*, *cpr1953*, and *cpr1954* genes in SM101 were individually disrupted by insertional mutagenesis using the *Clostridium*-modified TargeTron knockout system [20]. To verify that a group II intron had inserted into each targeted gene, PCR was performed using primers specific for internal *cpr1728*, *cpr1316*, *cpr1493*, *cpr1953*, or *cpr1954* open reading frame (ORF) sequences (S3A, S4A, S5A, S6A, and S7A Figs). Those primers amplified a larger PCR product for each mutant versus wild-type SM101 due to insertion of a 900 bp intron into each mutated putative kinase gene ORF (S3A, S4A, S5A, S6A, and S7A Figs). For example, using specific primers corresponding to internal *cpr1728* ORF sequences, PCR amplified a 275 bp product from SM101 DNA but a 1175 bp product from isogenic *cpr1728* null mutant DNA. The resulting mutants were designated CPR1728KO, CPR1316KO, CPR1493KO, CPR1953KO, and CPR1954KO, where “KO” represents knockout.

**Fig 3:**
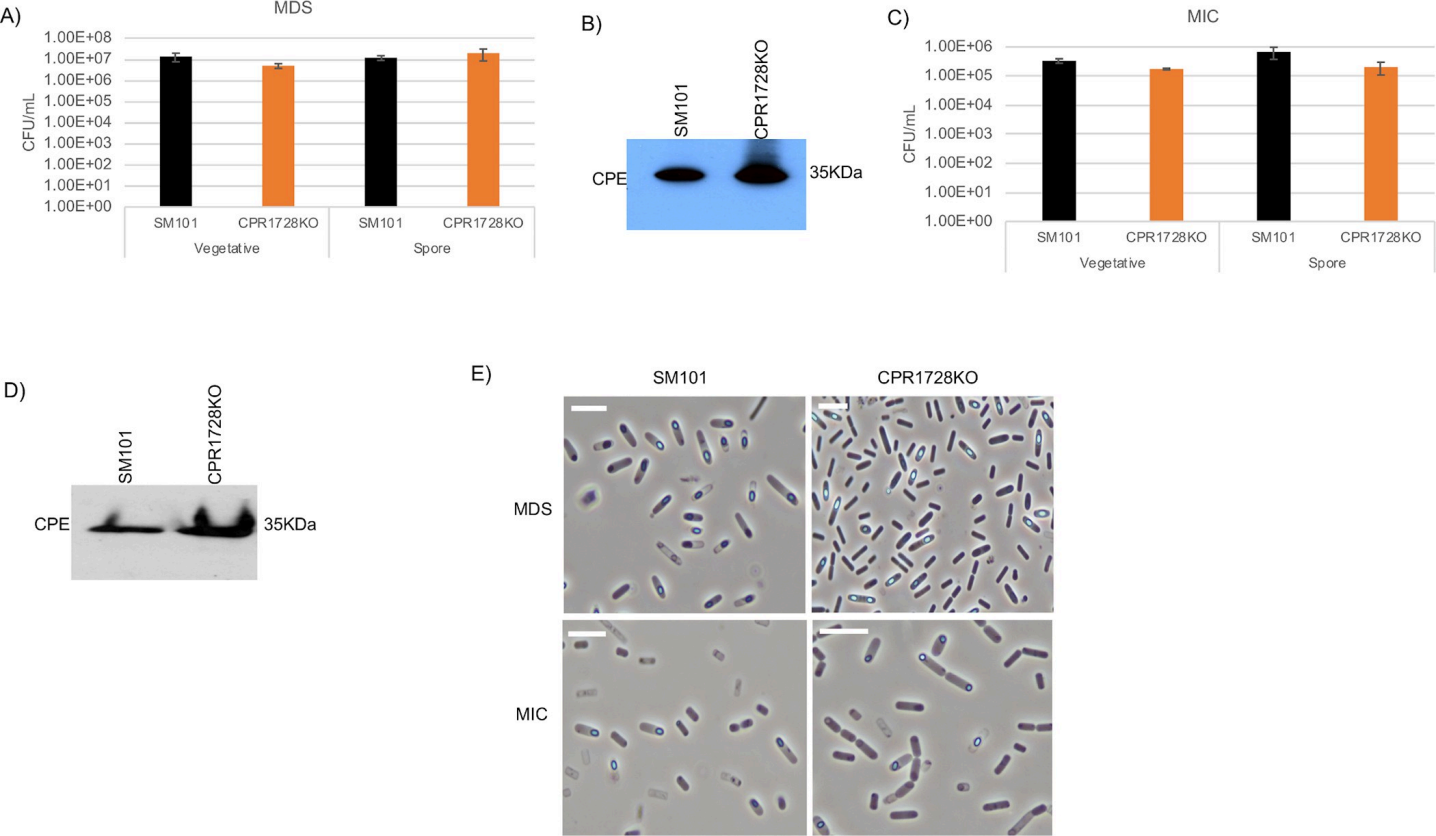
Evaluating the importance of CPR1728 for viable vegetative cell numbers, sporulation and CPE production when SM101 is cultured in MDS or MIC. (A) “Vegetative”, viable vegetative cells (CFU/mL) for SM101 and CPR1728KO, cultured overnight at 37°C in MDS. “Spores”, heat-resistant spores (CFU/mL) in aliquots of those same MDS cultures. (B) SM101 and CPR1728KO were cultured overnight at 37°C in MDS and supernatant of each culture was then subjected to Western blot analysis for CPE toxin production. (C) “Vegetative”, viable vegetative cells (CFU/mL) for SM101 and CPR1728KO cultured overnight at 37°C in MIC. “Spores”, heat-resistant spores (CFU/mL) for those same MIC cultures. (D) SM101 and CPR1728KO were cultured overnight at 37°C in MIC and supernatants of each culture was then subjected to Western blot analysis for CPE toxin production. (E) Phase-contrast photomicroscopy of SM101 and CPR1728KO grown in MDS or MIC to evaluate the presence of phase-refractile spores. The white scale bar represents 10 μm. Results for panels A and C are presented as the mean ± SD of three independent experiments. Student’s unpaired *t* test was used for statistical analysis of SM101 vs. CPR1728KO vegetative cell or spore numbers in panels A and C. *p* values were *<* 0.05 for all comparisons. Western blots shown in panels B and D are representative of three independent experiments. A loading control for this Western blot is presented in S2 Fig. Note that all MIC cultures contained 5% Oxyrase.

**Fig 4:**
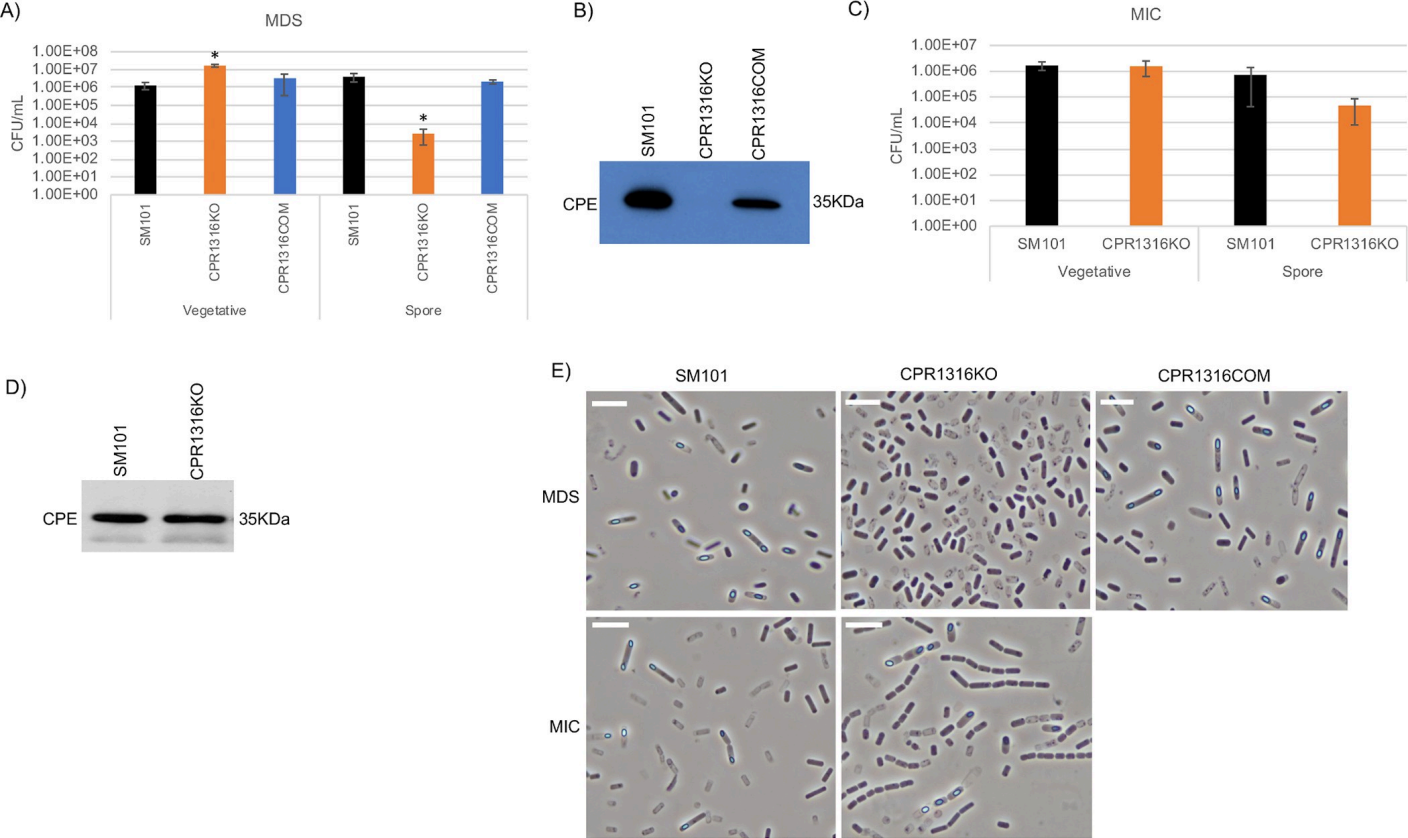
Evaluating the importance of CPR1316 for viable vegetative cell numbers, sporulation and CPE production when SM101 is cultured in MDS or MIC. (A) “Vegetative”, viable vegetative cells (CFU/mL) for SM101, CPR1316KO and CPR1316COM cultured overnight at 37°C in MDS. “Spores”, heat-resistant spores (CFU/mL) in aliquots of those same MDS cultures. (B) SM101, CPR1316KO and CPR1316COM were cultured overnight at 37°C in MDS and supernatant of each culture was then subjected to Western blot analysis for CPE toxin production. (C) “Vegetative”, viable vegetative cells (CFU/mL) for SM101 and CPR1316KO cultured overnight at 37°C in MIC. “Spores”, heat-resistant spores (CFU/mL) for those same MIC cultures. (D) SM101 and CPR1316KO were cultured overnight at 37°C in MIC and supernatant of each culture was then subjected to Western blot analysis for CPE toxin production. (E) Phase-contrast photomicroscopy of SM101, CPR1316KO, and CPR1316COM grown in MDS or MIC to evaluate the presence of phase-refractile spores. The white scale bar represents 10 μm. Results for panels A and C are presented as the mean ± SD of three independent experiments. Ordinary one-way analysis of variance (ANOVA; GraphPad Prism 8) was used for statistical analysis of vegetative cell or spore numbers for SM101 vs. its derivatives in panel A. Student’s unpaired *t* test was used for statistical analysis of vegetative or sporulating cultures of SM101 vs. CPR1316KO in panel C. Asterisk represents *p* < 0.05 versus SM101. Western blots shown in panels B and D are representative of three independent experiments. A loading control for this Western blot is presented in S2 Fig. Note that all MIC cultures contained 5% Oxyrase.

**Fig 5:**
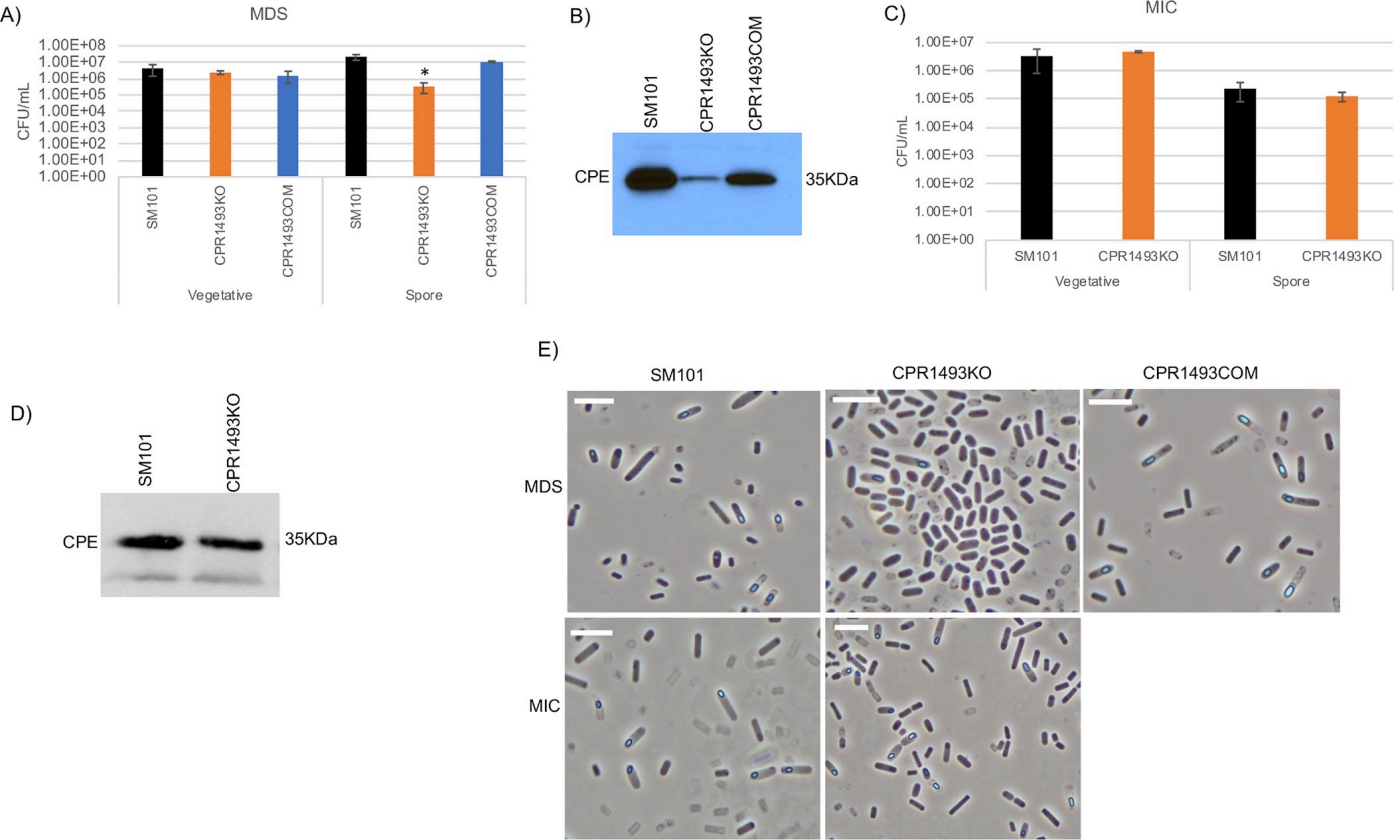
Evaluating the importance of CPR1493 for SM101 viable vegetative cell numbers, sporulation and CPE production when cultured in MDS or MIC. (A) “Vegetative”, viable vegetative cells (CFU/mL) for SM101, CPR1493KO and CPR1493COM cultured overnight at 37°C in MDS. “Spores”, heat-resistant spores (CFU/mL) in aliquots of those same MDS cultures. (B) SM101, CPR1493KO and CPR1493COM were cultured overnight at 37°C in MDS and supernatant of each culture was then subjected to Western blot analysis for CPE toxin production. (C) “Vegetative”, viable vegetative cells (CFU/mL) for SM101 and CPR1493KO cultured overnight at 37°C in MIC. “Spores”, heat-resistant spores (CFU/mL) for those same MIC cultures. (D) SM101 and CPR1493KO were cultured overnight at 37°C in MIC and supernatant of each culture was then subjected to Western blot analysis for CPE toxin production. (E) Phase-contrast photomicroscopy of SM101, CPR1493KO and CPR1493COM grown in MDS or MIC to evaluate the presence of phase-refractile spores. The white scale bar represents 10 μm. Results for panels A and C are presented as the mean ± SD of three independent experiments. Ordinary one-way analysis of variance (ANOVA; GraphPad Prism 8) was used for statistical analysis of vegetative cell or spore numbers for SM101 and its derivatives in panel A. Student’s unpaired *t* test was used for statistical analysis of vegetative cell or spore numbers for SM101 vs. CPR1493KO in panel C. Asterisk represents *p* < 0.05 versus SM101. Western blots shown in panels B and D are representative of three independent experiments. A loading control for this Western blot is presented in S2 Fig. Note that all MIC cultures contained 5% Oxyrase.

**Fig 6:**
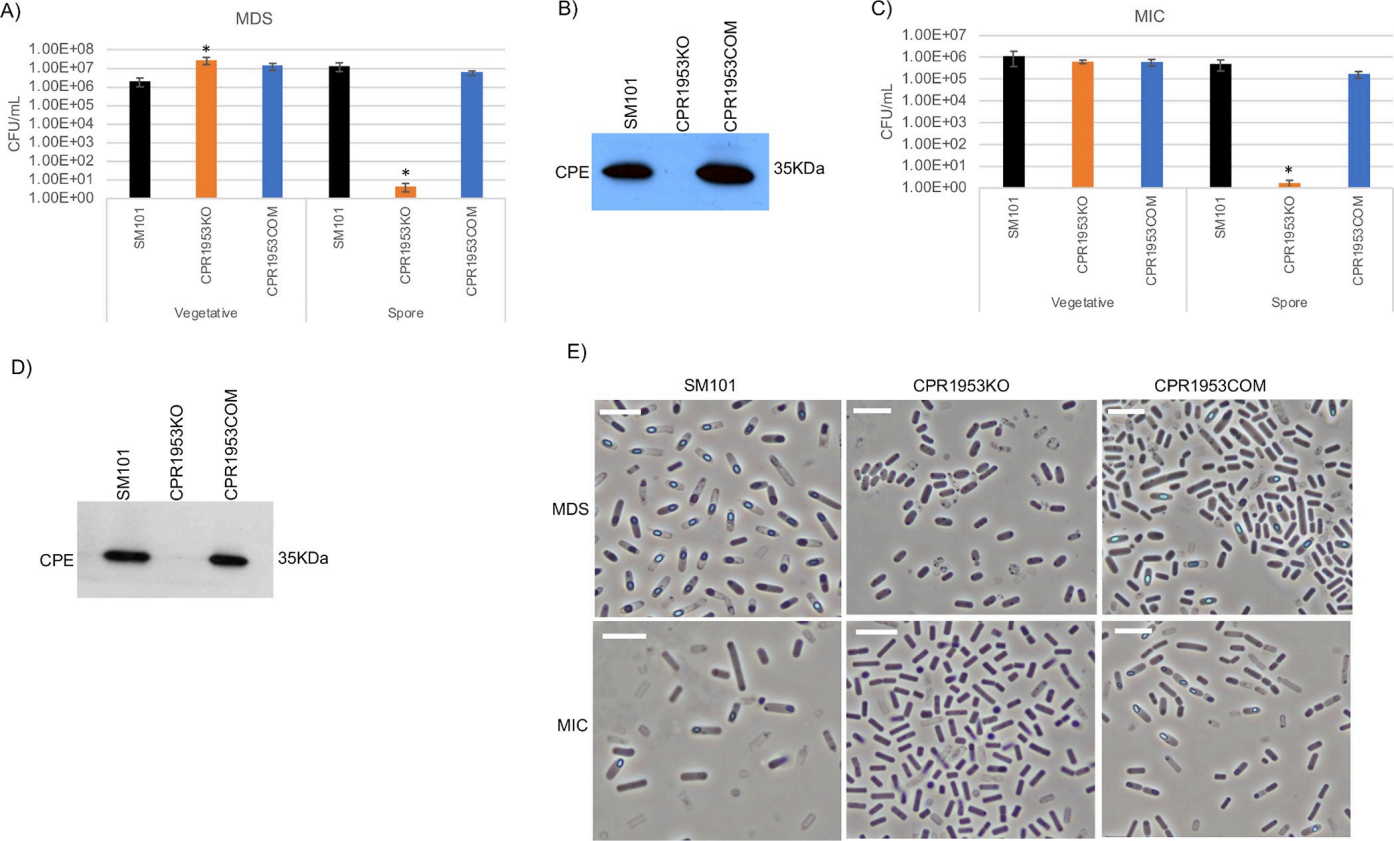
Evaluating the importance of CPR1953 for viable vegetative cell numbers, sporulation and CPE production when SM101 is cultured in MDS or MIC. (A) “Vegetative”, viable vegetative cells (CFU/mL) for SM101, CPR1953KO, and CPR1953COM cultured overnight at 37°C in MDS. “Spores”, heat-resistant spores (CFU/mL) in aliquots of those same MDS cultures. (B) SM101, CPR1953KO and CPR1953COM were cultured overnight at 37°C in MDS and supernatant of each culture was then subjected to Western blot analysis for CPE toxin production. (C) “Vegetative”, viable vegetative cells (CFU/mL) for SM101, CPR1953KO and CPR1953COM cultured overnight at 37°C in MIC. “Spores”, heat-resistant spores (CFU/mL) for those same MIC cultures. (D) SM101, CPR1953KO and CPR1953COM were cultured overnight at 37°C in MIC and supernatant of each culture was then subjected to Western blot analysis for CPE toxin production. (E) Phase-contrast photomicroscopy of SM101, CPR1953KO, and CPR1953COM grown in MDS or MIC to evaluate the presence of phase-refractile spores. The white scale bar represents 10 μm. Results for panels A and C are presented as the mean ± SD of three independent experiments. Ordinary one-way analysis of variance (ANOVA; GraphPad Prism 8) was used for statistical analysis of vegetative cells or spores of SM101 and its derivatives in panels A and C. Asterisk represents *p* < 0.05 versus SM101. Western blots shown in panels B and D are representative of three independent experiments. A loading control for this Western blot is presented in S10 Fig. Note that all MIC cultures contained 5% Oxyrase.

**Fig 7:**
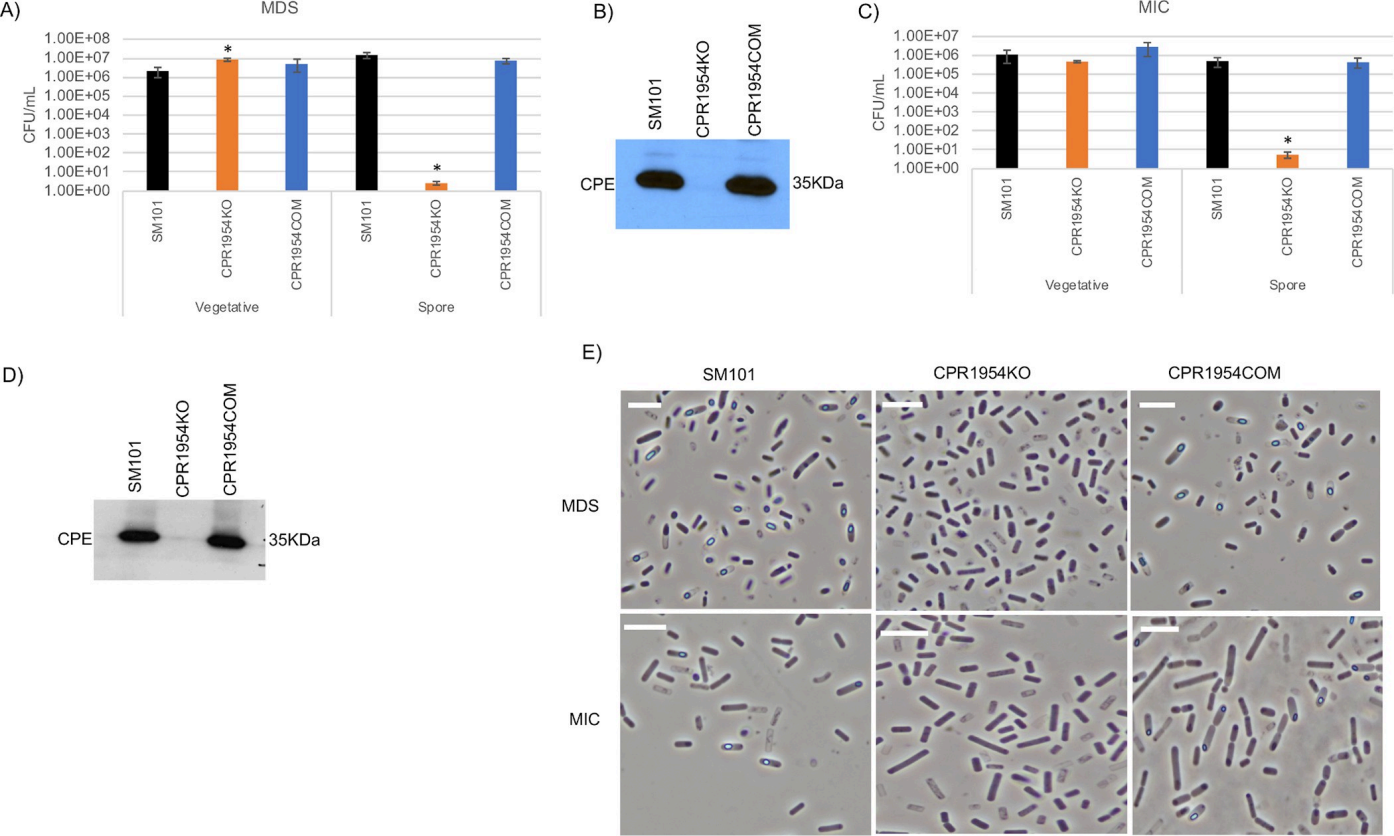
Evaluating the importance of CPR1954 for SM101 viable vegetative cell numbers, sporulation and CPE production when cultured in MDS or MIC. (A) “Vegetative”, viable vegetative cells (CFU/mL) for SM101, CPR1954KO, and CPR1954COM cultured overnight at 37°C in MDS. “Spores”, heat-resistant spores (CFU/mL) in aliquots of those same MDS cultures. (B) SM101, CPR1954KO and CPR1954COM were cultured overnight at 37°C in MDS and supernatants of those cultures were then subjected to Western blot analysis for CPE toxin production. (C) “Vegetative”, viable vegetative cells (CFU/mL) for SM101, CPR1954KO and CPR1954COM cultured overnight at 37°C in MIC. “Spores”, heat-resistant spores (CFU/mL) for those same MIC cultures. (D) SM101, CPR1954KO and CPR1954COM were cultured overnight at 37°C in MIC and supernatants of those cultures were then subjected to Western blot analysis for CPE toxin production. (E) Phase-contrast photomicroscopy of SM101, CPR1954KO, and CPR1954COM grown in MDS or MIC to evaluate the presence of phase-refractile spores. The white scale bar represents 10 μm. Results for panels A and C are presented as the mean ± SD of three independent experiments. Ordinary one-way analysis of variance (ANOVA; GraphPad Prism 8) was used for statistical analysis of vegetative cell or spore numbers of SM101 and derivatives in panels A and C. Asterisk represents *p* < 0.05 versus SM101. Western blots shown in panels B and D are representative of three independent experiments. A loading control for this Western blot is presented in S10 Fig. Note that all MIC cultures contained 5% Oxyrase.

The presence of a single intron insertion in each null mutant strain was confirmed by Southern blot analysis using a probe specific for the group II intron (S3B, S4B, S5B, S6B, and S7B Figs). To confirm loss of wild-type mRNA expression for each mutated kinase gene, RT-PCR was performed. As controls, all mutant strains were shown by RT-PCR to express the *16S* RNA gene, verifying the quality of purified RNAs. In addition, no amplification of purified *polC* RNA was observed without the addition of reverse transcriptase, confirming the purity of each prepared RNA (S3C, S4C, S5C, S6C, and S7C Figs). Using those same RNA preparations, RT-PCR analyses indicated that expression of wild-type mRNA for the *cpr1728*, *cpr1316*, *cpr1493*, *cpr1953*, or *cpr1954* genes was eliminated in CPR1728KO, CPR1316KO, CPR1493KO, CPR1953KO, and CPR1954KO null mutants. For most tested cultures of these mutants, no detectable mRNA for the inactivated gene was present (S3C, S4C, S5C, S6C, and S7C Figs). However, occasionally a large mRNA band was present (see S8 Fig as an example) in some cultures; this band corresponds to the presence of some intact intron-disrupted mRNA as has been sometimes observed previously using Targetron mutagenesis [21]. Notably, the phenotypes of all cultures for each mutant were stable, as shown in Figs 3–7, and wild-type phenotypes could be restored by complementation (see below), confirming the identity of these intron-disrupted strains as null mutants. Last, growth curve analyses showed that the growth rate of each isogenic orphan histidine kinase null mutant in MDS was similar to that of the SM101 parent strain (S3D, S4D, S5D, S6D, and S7D Figs).

When a mutant exhibited decreased sporulation (see Results below), it was complemented to address possible secondary mutations or polar effects. To create a complementing strain for these putative kinase null mutants, the wild-type *cpr1316*, *cpr1493*, *cpr1953*, or *cpr1954* ORFs, plus approximately 800 bp of their upstream sequence, were each cloned separately into the *C*. *perfringens*-*E*. *coli* shuttle plasmid pJIR750 [22]. The resultant plasmids were then electroporated into their relevant mutant strain to prepare the complementing strains, which were named CPR1316COM, CPR1493COM, CPR1953COM, and CPR1954COM (where “COM” represents complementing). The complementing strains were then verified by PCR assay using the same internal primers that were employed for screening orphan histidine kinase genes, which amplified a product matching the product size amplified from wild-type SM101 (S4A, S5A, S6A, and S7A Figs). To confirm complementation of the mutants, RT-PCR assays were performed to assess expression of each target gene. The results detected expression of wild-type mRNA (no intron disruption) by the *cpr1316*, *cpr1493*, *cpr1953*, and *cpr1954* genes in CPR1316COM, CPR1493COM, CPR1953COM, and CPR1954COM strains, respectively (S4C, S5C, S6C, and S7C Figs). When the growth of each complementing strain in MDS culture was evaluated by measuring the change in the optical density at OD_600_ at different incubation time points, each complementing strain displayed similar growth as wild-type SM101 or the corresponding isogenic null mutant strain (S4D, S5D, S6D, and S7D Figs).

It was also confirmed that SM101 carrying pJIR750 alone (empty vector control) in the presence of chloramphenicol (cm) had wild-type levels of viable vegetative cell numbers, spores numbers and CPE production when this transformant was cultured in MDS or MIC (S9 Fig).

### Characterization of vegetative cell viability, sporulation and CPE production by the *cpr1728* null mutant when cultured in MDS vs. MIC

The phenotype of the CPR1728KO mutant was assessed by enumerating the presence of viable vegetative cells or heat-resistant spores in overnight MDS or MIC cultures. As shown in Fig 3A, wild-type levels of viable CPR1728KO vegetative cells and heat-resistant spores were present in overnight MDS cultures. Under this culture condition, CPR1728KO also produced wild-type CPE levels (Fig 3B). Similarly, no significant differences were detected between the number of viable vegetative cells or heat-resistant spores present in overnight MIC cultures of the CPR1728KO mutant vs. wild-type SM101 (Fig 3C). Furthermore, the CPR1728KO mutant also produced wild-type levels of CPE in those overnight MIC cultures (Fig 3D). Confirming the heat-resistant spore plating results for CPR1728KO, phase-contrast photomicroscopy detected the presence of phase-refractile spores when either CPR1728KO or SM101 were cultured for 12 h in MDS or 18 h in MIC (Fig 3E).

Collectively, these experiments indicated that expression of the *cpr1728* gene, which encodes a putative orphan histidine kinase, is not necessary for wild-type levels of sporulation or CPE production when SM101 is cultured in either MDS or MIC.

### CPR1316 and CPR1493 are important for sporulation and CPE production when SM101 is cultured in MDS but not MIC

The importance of the putative CPR1316 or CPR1493 orphan histidine kinases for vegetative cell viability, sporulation and CPE production was next evaluated for SM101 cultured overnight in MDS or MIC. Relative to overnight SM101 MDS cultures, there was a small, but significant, increase in the numbers of viable vegetative cells present in overnight CPR1316KO MDS cultures and this effect was reversible by complementation (Fig 4A). Similarly, approximately wild-type levels of viable vegetative cells were present in MDS cultures of CPR1493KO (Fig 5A).

In overnight MDS cultures (Figs 4A and 5A), both CPR1316KO and CPR1493KO formed significantly fewer heat-resistant spores than wild-type SM101. Since those mutants exhibited a significant phenotype, the complementing strains CPR1316COM and CPR1493COM were tested for their sporulation in overnight MDS cultures, which revealed formation of wild-type levels of heat-resistant spores by both strains (Figs 4A and 5A). CPE Western blotting of MDS cultures (Figs 4B and 5B) revealed no or reduced CPE production by CPR1316KO and CPR1493KO, respectively, while CPE production was apparent in overnight MDS cultures of wild-type SM101 or the complementing strains.

Wild-type levels of CPR1316KO or CPR1493KO viable vegetative cells were present in their overnight MIC cultures (Figs 4C and 5C). Both mutants also produced near wild-type levels of heat-resistant spores in MIC (Figs 4C and 5C). Furthermore, these mutants also produced similar CPE levels as wild-type SM101 under this culture condition (Figs 4D and 5D).

Phase-contrast photomicroscopy (Figs 4E and 5E) of 12 h cultures supported the heat-resistant spore plating results and addressed the possibility that, when cultured in MDS, CPR1316KO or CPR1493KO might form spores that are heat-sensitive. Specifically, negligible numbers of phase-refractile spores were present in 12 h MDS cultures of CPR1316KO or CPR1493KO compared to SM101, while complementation of either mutant restored the presence of phase-refractile spores in 12 h MDS cultures. However, in 18 h MIC cultures, both mutants produced readily detectable phase-refractive spores, similarly as SM101.

Collectively, these results show that, like the CPR0195 kinase, the CPR1316 and CPR1493 kinases are important for sporulation in MDS but dispensable for sporulation in MIC.

### Evidence that CPR1953 and CPR1954 are important for SM101 sporulation and CPE production in both MDS and MIC cultures

To evaluate if CPR1953 or CPR1954 are important for vegetative cell viability, sporulation and CPE production in MDS or MIC cultures of SM101, the phenotypes of CPR1953KO and CPR1954KO were assessed. Overnight MDS cultures of CPR1953KO and CPR1954KO contained slightly more viable vegetative cells than SM101 MDS cultures (Figs 6A and 7A), while overnight MIC cultures of either mutant contained wild-type levels of viable vegetative cells (Figs 6C and 7C).

CPR1953KO and CPR1954KO both formed negligible (<10/ml) numbers of heat-resistant spores in overnight MDS cultures, in contrast to the strong (~10^6^ heat-resistant spores/mL) formation of heat-resistant spores by SM101 in this same culture condition (Figs 6A and 7A). Furthermore, in overnight MDS cultures, no CPE production was observed for either kinase null mutant, although CPE production was readily detectable in overnight MDS cultures of SM101 (Figs 6B and 7B). Complementing either mutant with its relevant kinase ORF and ~800 bp of corresponding upstream region reversed their sporulation deficiency and restored wild-type levels of CPE production for overnight MDS cultures (Figs 6A,6B, 7A and 7B).

Importantly, insertional disruption of either the *cpr1953* or *cpr1954* genes also caused a >10,000-fold decrease in the number of heat-resistant spores, as well as loss of detectable CPE production, when those mutants were cultured overnight in MIC (Figs 6C, 6D, 7C and 7D). Furthermore, complementation of those mutants restored their production of wild-type levels of heat-resistant spores and CPE (Figs 6C, 6D, 7C and 7D). The virtual absence of spore formation by CPR1953KO and CPR1954KO when cultured in either MDS or MIC was supported by phase-contrast microscopy analysis, which did not detect the presence of phase-refractile spores in either 12 h MDS or 18 h in MIC cultures (Figs 6E and 7E).

Collectively, these results strongly suggest that the *cpr1953* and *cpr1954* orphan histidine kinase genes play a critical role in regulating sporulation and CPE production when SM101 is cultured in either MDS or MIC.

### Analysis of CPR1953 and CPR1954 expression by SM101

**Fig 8:**
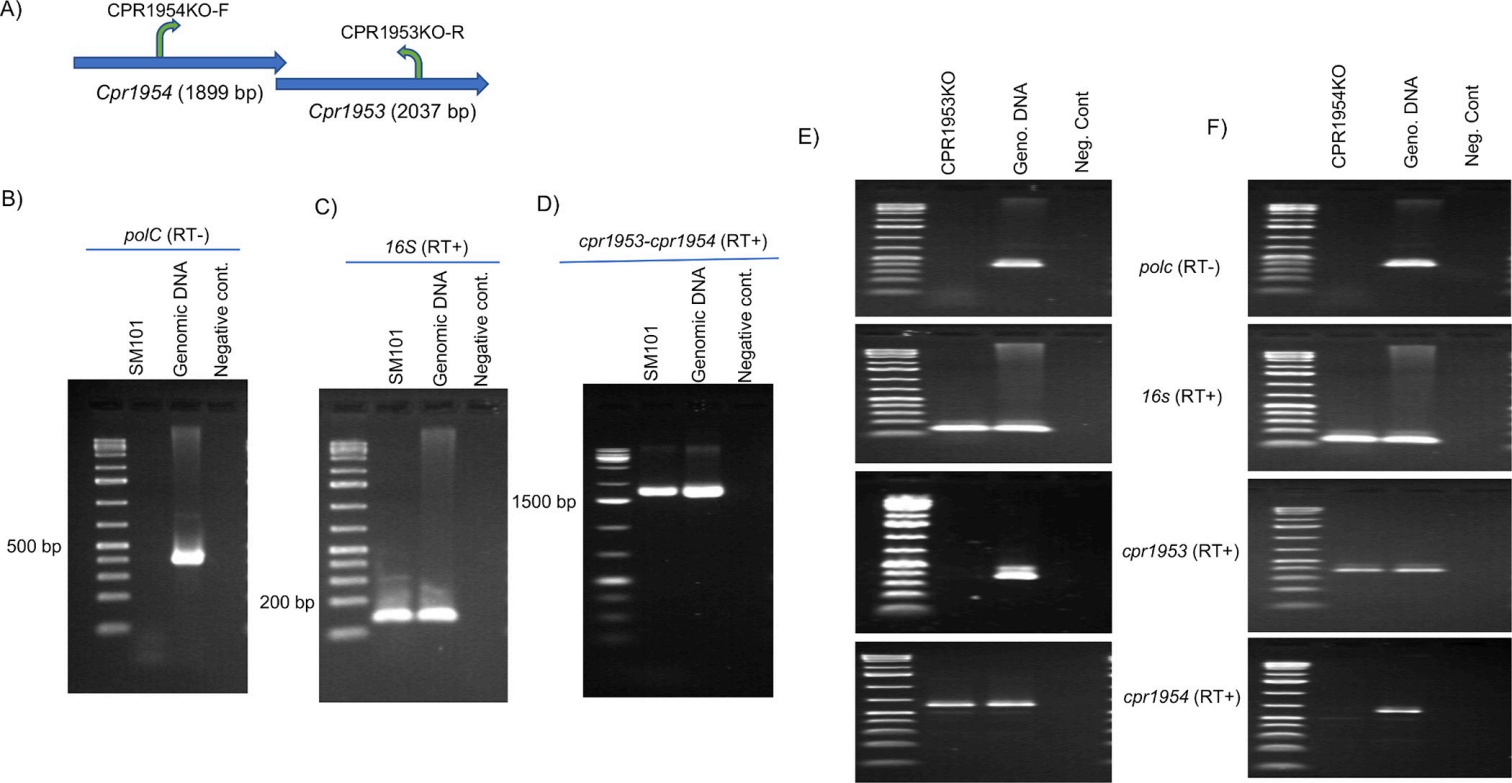
Evidence that the genes encoding CPR1953 and CPR1954 can be co-expressed in an operon. (A) Arrangement and orientation of the *cpr1954* and *cpr1953* genes on the SM101 chromosome. (B) RNA was isolated from wild-type SM101 grown in MDS for 2 h at 37°C. To demonstrate the purity of each isolated RNA, samples were subjected to PCR for the *polC* housekeeping gene without the use of reverse transcriptase. Genomic DNA or a sample lacking DNA template were used as positive and negative controls, respectively. (C) RT-PCR analysis for *16S* housekeeping gene as a control for the quality of each prepared RNA. (D) RT-PCR analysis for expression of *cpr1953* and *cpr1954* genes using CPR1954KO-F and CPR1953KO-R primers. The experiment was performed three times and the picture presented is representative of three independent repetitions. (E) and (F) RT-PCR analyses of *cpr1953* and *cpr1954* expression by CPR1953KO and CPR1954KO. To demonstrate the purity of RNA isolated from 2h MDS cultures of CPR1953KO (E) or CPR1954KO (F), samples were subjected to PCR for the *polC* housekeeping gene without the use of reverse transcriptase (top panels of (E) and (F)). Genomic DNA or a sample lacking DNA template were used as positive and negative controls, respectively. RT-PCR analysis for *16S* housekeeping gene as a control for the quality of each prepared RNA (second panel from top in (E) and (F)). Bottom two panels in (E) and (F) show RT-PCR analysis for expression of *cpr1953* (CPR1953KO-F and CPR1953KO-R primers) and *cpr1954* (CPR1954KO-F and CPR1954KO-R primers).

Bioinformatic analysis of the SM101 genome [23] indicated that the *cpr1953* and *cpr1954* orphan histidine kinase genes are arranged in the same orientation on the chromosome, where they share a 20 nucleotide overlap (Fig 8A). Therefore, RT-PCR analysis was performed using RNA isolated from SM101 grown in MDS medium for 2 h at 37°C to assess whether these two genes can be co-expressed as an operon. Purity of the extracted RNA was confirmed by the requirement for reverse transcriptase to amplify a *polC* RNA product (Fig 8B). Quality of the isolated RNA was verified by demonstration of *16S* RNA expression (Fig 8C). When the primer pair 1954KO-F and 1953KO-R (Fig 8A, and see Materials and Methods) were employed, a large product was amplified that corresponds to a co-transcript of both genes on a single mRNA (Fig 8D).

This RT-PCR result suggested that *cpr1953* and *cpr1954* can be co-expressed as an operon, which might indicate that CPR1954KO or CPR1953KO are double *cpr1954-cpr1953* null mutants since those mutants were created by insertional disruption. If that situation were true, CPR1954KO should not express *cpr1953* and CPR1953KO should nor express *cpr1954*. However, when this possibility was assessed by RT-PCR, CPR1953KO still expressed *cpr1954* (Fig 8E) and CPR1954KO still expressed *cpr1953* (Fig 8F). Same primer sets as described earlier were used for assessing the expression of *cpr1953* and *cpr1954* genes (See Materials and Methods as well as S6C and S7C Figs).

### Distinguishing whether elimination of *cpr1316*, *cpr1493*, *cpr1953*, or *cpr1954* gene expression delays vs. permanently impairs sporulation in SM101

**Fig 9:**
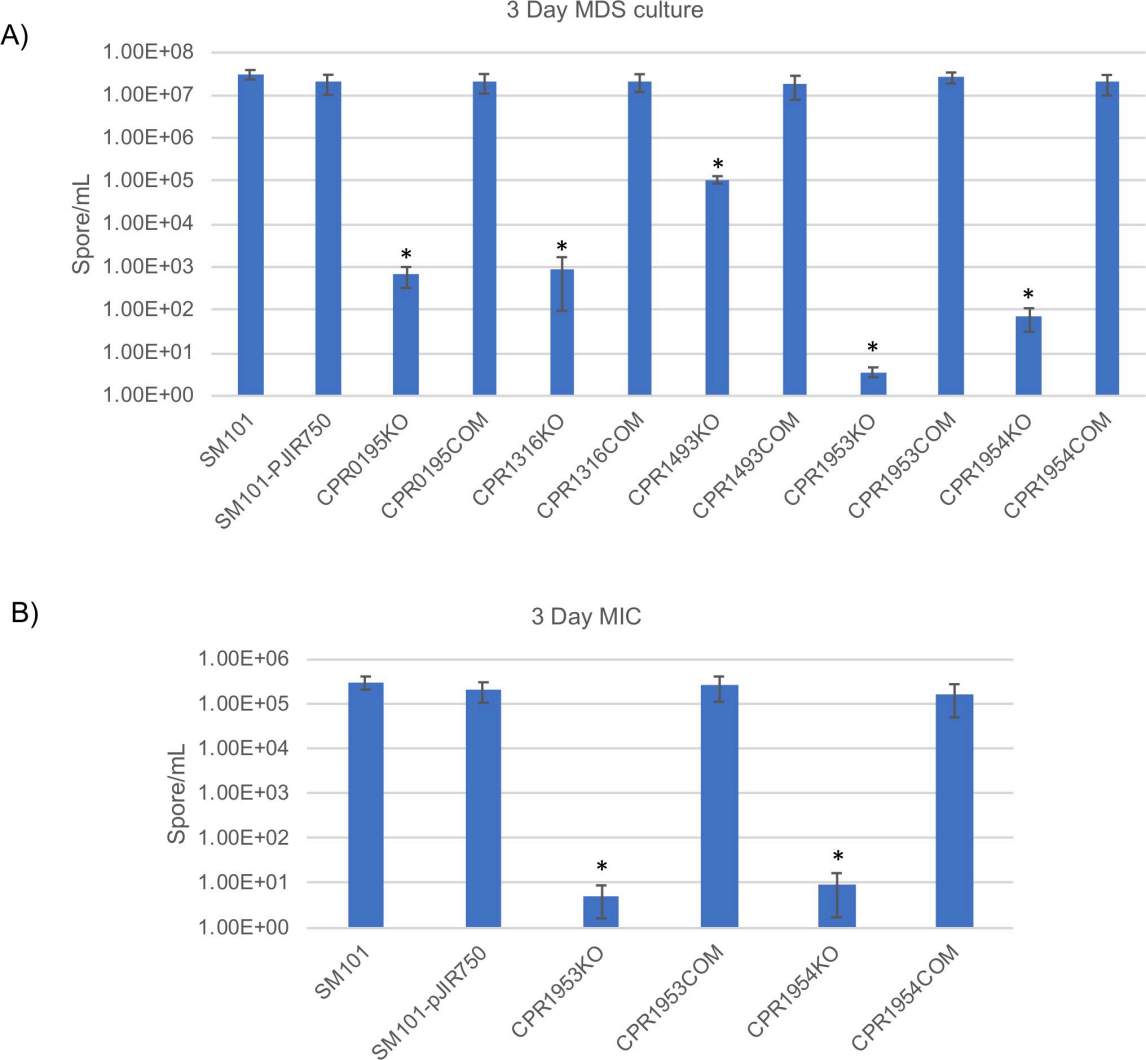
Disruption of the *cpr1316*, *cpr1493*, *cpr1953*, or *cpr1954* genes causes long-term sporulation impairment. To assess whether the sporulation defects observed in CPR1316KO, CPR1493KO, CPR1953KO, and CPR1954KO are permanent or merely delayed, these mutants and their complementing strains were grown in MDS (A) or MIC (B) for 72 h at 37°C; aliquots were then heat-shocked at 70°C and plated onto BHI agar. All results are presented as the mean ± SD of three independent experiments. Ordinary one-way analysis of variance (ANOVA; GraphPad Prism 8) was used for statistical analysis in panels A and B. Asterisk represents *p* < 0.05 versus SM101.

The overnight MDS culture sporulation defects observed in Figs 2 and 4–7 for CPR0195KO, CPR1316KO, CPR1493KO, CPR1953KO, and CPR1954KO could be indicative of either delayed or permanently impaired sporulation. To discriminate between those possibilities, each mutant was cultured in MDS and, for CPR1953KO and CPR1954KO, MIC for 72 h at 37°C. When aliquots of those cultures were heat-shocked, plated onto BHI agar and incubated anaerobically, results showed that (even with this extended incubation time) all mutants still had significantly reduced sporulation relative to wild-type SM101 (Fig 9A and 9B). Furthermore, these phenotypes were reversible by complementation. These results support long-term impairment of sporulation for CPR1953KO and CPR1954KO.

### Evidence that CPR1316, CPR1493, CPR1953, and CPR1954 are involved early during sporulation of SM101 in MDS cultures

SigF (encoded by the *spoIIA* operon) is a critical early sigma factor during *C*. *perfringens* sporulation [11,12]. Our previous study used a reporter plasmid [24] consisting of the *C*. *perfringens-E*. *coli* pJIR750 shuttle plasmid carrying the beta-D-glucuronidase ORF of the *E*. *coli gusA* gene fused to the *sigF* promoter region to demonstrate that CPR0195 acts early during sporulation, i.e., before *sigF* expression occurs [17].

**Fig 10:**
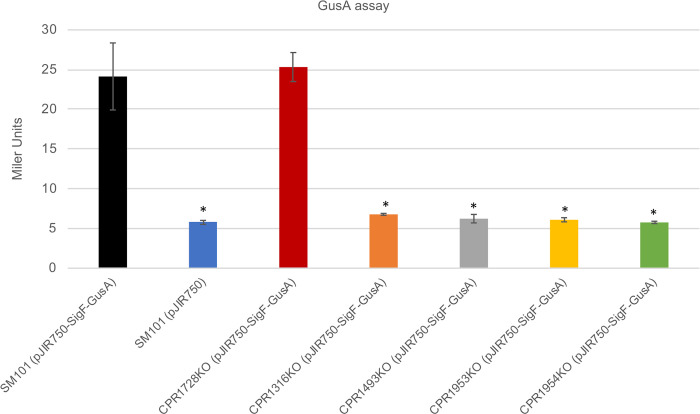
Evidence that CPR1316, CPR1493, CPR1953, and CPR1954 function early during sporulation in SM101 MDS cultures. A reporter plasmid [24] containing the *E*. *coli* beta-D-glucuronidase gene (*gusA*) ORF fused to the *C*. *perfringens sigF* promoter region, which drives expression of the early sporulation-produced SigF, was transferred into wild-type SM101 and the putative kinase null mutants. After overnight growth in MDS at 37°C, the levels of GusA activity for each transformant were measured. An empty pJIR750 plasmid separately transferred into wild-type SM101 served as a negative control. The results are presented as the mean ± SD of three independent experiments. Ordinary one-way analysis of variance (ANOVA; GraphPad Prism 8) was used for statistical analysis. Asterisk represents *p* < 0.05 versus SM101 (pJIR750-SigF-GusA).

The current study transformed that same reporter plasmid into wild-type SM101 or each of the five putative kinase mutants created in this study. Empty (no insert) pJIR750 plasmid was also transformed into SM101 to serve as a negative control [17]. When sonicated lysates of overnight MDS cultures were mixed with a 6 mM 4-nitrophenyl-β-d-glucuronide substrate and GusA activity levels measured (Fig 10), GUS activity was significantly higher for SM101 transformed with the reporter plasmid vs the negative control empty plasmid. For these reporter plasmid transformants, SM101 GUS activity was also significantly greater than the GUS activity measured for any putative kinase null mutant with the exception of CPR1728KO. This result for CPR1728KO is consistent with the Fig 3 observation that CPR1728KO does not show a sporulation defect when cultured in MDS, indicating that it must still express from the *sigF* promoter when cultured in MDS.

Collectively, these results demonstrated that CPR1316, CPR1493, CPR1953, and CPR1954 all contribute early, i.e., prior to initiation of *sigF* transcription, to SM101 sporulation. This experiment was only performed in MDS cultures because MIC interfered with GusA activity measurements, i.e., MIC itself had too high of a background for accurate measurements.

### Evaluation of *cpr1728*, *cpr1316*, *cpr1493*, *cpr1953* and *cpr1954* expression during vegetative growth

Since *C*. *perfringens* vegetative cells develop into sporulating cells, orphan histidine kinase genes involved in initiating sporulation should be expressed under vegetative conditions so these kinases can be available when (still unidentified) environmental and/or cellular signals are received to start sporulation. Since this was shown previously for *cpr0195* and *cpr1055* [17], expression of the *cpr1728*, *cpr1316*, *cpr1493*, *cpr1953* and *cpr1954* genes during late-logarithmic-phase vegetative growth conditions, i.e., 6 h TGY broth cultures, were evaluated by RT-PCR assay.

**Fig 11:**
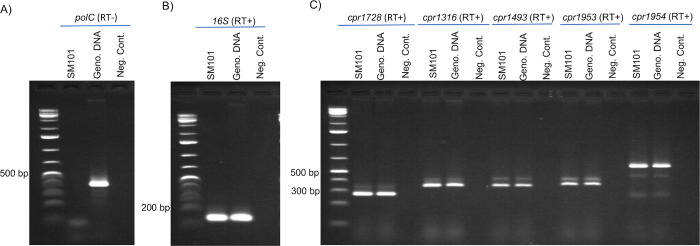
Expression of the *cpr1728*, *cpr1316*, *cpr1493*, *cpr1953*, and *cpr1954* genes in SM101 vegetative cultures. RNA was isolated from wild-type SM101 grown in TGY broth for 6 h at 37°C. (A) To show the purity of this isolated RNA, the sample was subjected to PCR, without reverse transcriptase, for the *polC* housekeeping gene. Genomic DNA and a sample lacking DNA template were used as positive and negative controls, respectively. (B) RT-PCR analysis for the *16S* housekeeping gene as a control to demonstrate the quality of prepared RNA. (C) RT-PCR analysis for expression of *cpr1728*, *cpr1316*, *cpr1493*, *cpr1953*, and *cpr1954* genes by SM101 cultured in TGY vegetative growth medium. The experiment was performed three times and the results shown are representative of three independent repetitions.

The purity and quality of RNA extracted from 6 h TGY vegetative cultures of wild-type SM101 was verified as described earlier (Fig 11A and 11B). When those highly-purified RNA samples were subjected to RT-PCR using specific primer sets (see Materials and Methods) to assess the expression of each kinase gene, the results showed that the *cpr1728*, *cpr1316*, *cpr1493*, *cpr1953* and *cpr1954* genes were each expressed under this vegetative growth condition (Fig 11C). These results support the availability of these orphan histidine kinase proteins during the transition when vegetative cells enter sporulation.

### Effects of CPR1316, CPR1493, CPR1953, and CPR1954 on Spo0A production (for MDS cultures) or *spo0A* expression (for MIC cultures)

To explore whether CPR1316, CPR1493, CPR1953, or CPR1954 affect Spo0A production, wild-type SM101 or its derivative putative orphan histidine kinase mutants or complemented strains were cultured in MDS medium for 3 h or 5 h at 37°C; equal numbers of cells were then lysed and equal amount of total proteins were subjected to Western blot analysis using rabbit polyclonal antiserum raised against *Clostridioides difficile* Spo0A. In this experiment, a SM101 *spo0A* null mutant named IH101 [10] was used as a negative control.

**Fig 12:**
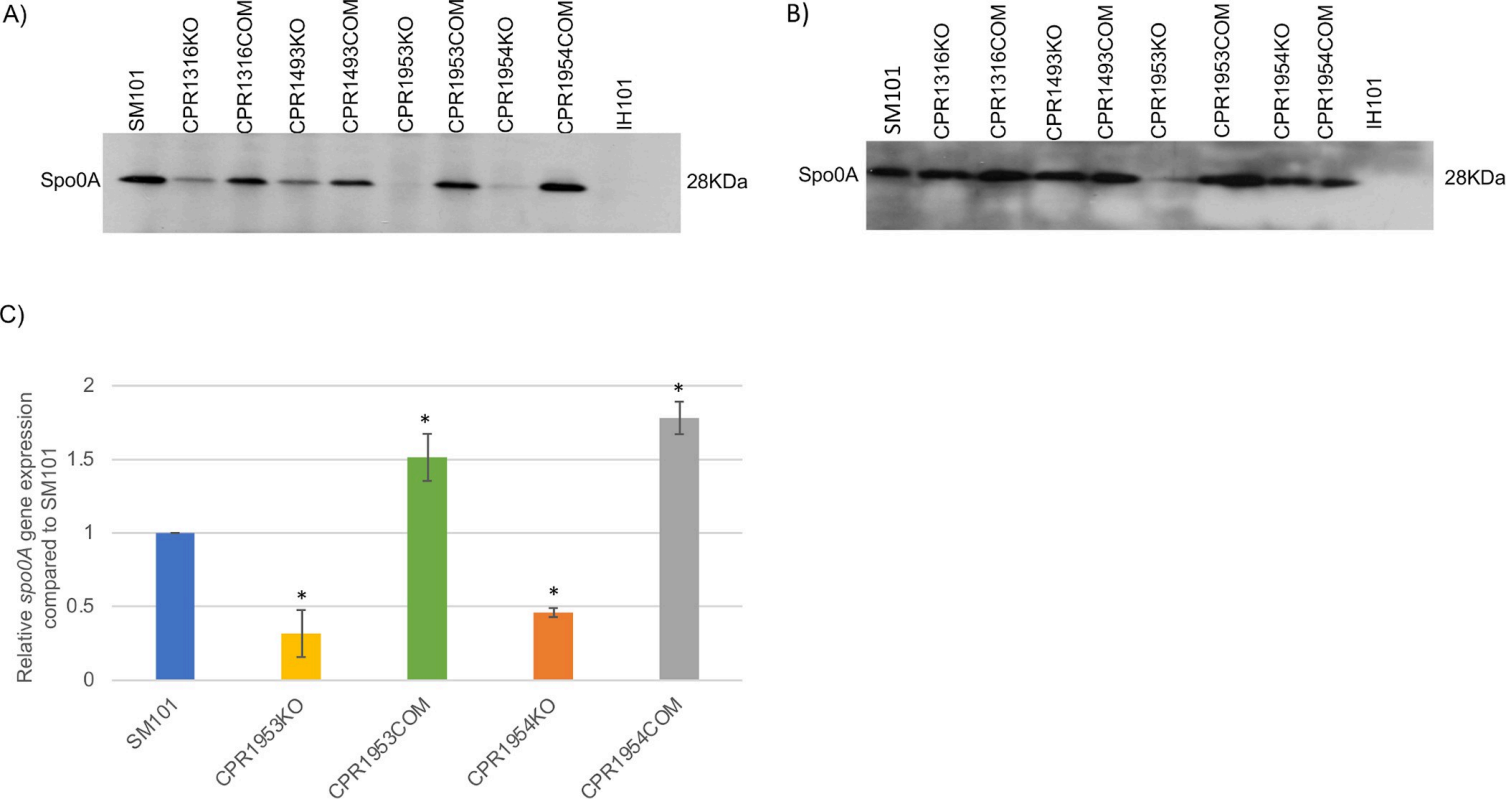
Evaluation of CPR1316, CPR1493, CPR1953, and CPR1954 effects on Spo0A production in MDS or MIC cultures. The indicated strains were grown in MDS for three (A) or five (B) hours at 37°C. Equal numbers of cells for each strain were lysed and an equal amount of total proteins was subjected to Western blot analysis for Spo0A production using rabbit polyclonal antiserum raised against *C*. *difficile* Spo0A. The experiment was performed three times and the result shown is representative of three independent repetitions. (C) To examine the effect of indicated mutations on *spo0A* expression levels in MIC cultures, each strain was cultured in MIC containing 5% Oxyrase for 3 h. RNAs were extracted from indicated samples and subjected to qRT-PCR to quantify expression of *spo0A*. The result is presented as the mean ± SD of three independent experiments. Ordinary one-way analysis of variance (ANOVA; GraphPad Prism 8) was used for statistical analysis in panel C. Asterisk represents *p* < 0.05 versus SM101. Loading controls for panels A and B are shown in S10 Fig.

These Western blot analyses detected Spo0A production by SM101, but not by its *spo0A* null mutant IH101, in 3 h or 5 h MDS cultures (Fig 12A and 12B). They also showed that, similar to a previous report [17] for CPR0195KO, CPR1316KO and CPR1493KO produce less Spo0A than wild-type SM101 (Fig 12A) when cultured for 3 h in MDS. In similar 3 h MDS cultures, CPR1953KO did not produce detectable levels of Spo0A while, relative to SM101, CPR1954KO made reduced levels of Spo0A (Fig 12A). When the study was extended to 5 h of culture in MDS, the CPR1953KO null mutant strain still produced very little Spo0A although, with that longer incubation, the CPR1316KO, CPR1493KO, and CPR1954KO mutants all made Spo0A at approximately wild-type levels (Fig 12B).

Western blot analysis of Spo0A production in MIC cultures would have required use of prohibitively large numbers of mice to obtain sufficient MIC volumes to culture enough *C*. *perfringens* cells as needed for Spo0A Western blot sensitivity. Instead, *spo0A* expression levels in 3 h MIC cultures of CPR1953KO or CPR1954KO were assessed using the more-sensitive quantitative reverse transcription PCR (qRT-PCR). This approach detected significantly lower *spo0A* expression levels in MIC cultures of CPR1953KO and CPR1954KO compared to SM101. Furthermore, complementation restored wild-type *spo0A* expression (Fig 12C).

### Evaluating the involvement of putative orphan histidine kinase proteins in Spo0A phosphorylation

The current study next addressed whether the five putative orphan histidine kinase genes mutated for the first time during this study are important for Spo0A phosphorylation, which is considered a necessary step for initiating *C*. *perfringens* sporulation. For this purpose, equal amounts of total protein from 3 h MDS culture lysates of SM101, its derivative putative kinase mutants, or complemented strains were subjected to Phos-Tag SDS-PAGE, which separates the phosphorylated form of Spo0A (Spo0A~P) from its unphosphorylated form (Spo0A), followed by Spo0A Western blotting. Lysates from 3 h MDS cultures of the SM101 *spo0A* null mutant (IH101) or heated MDS lysate from wild-type SM101 strain were used as controls for detection of phosphorylated Spo0A (note that heating dephosphorylates Spo0A [25]).

**Fig 13:**
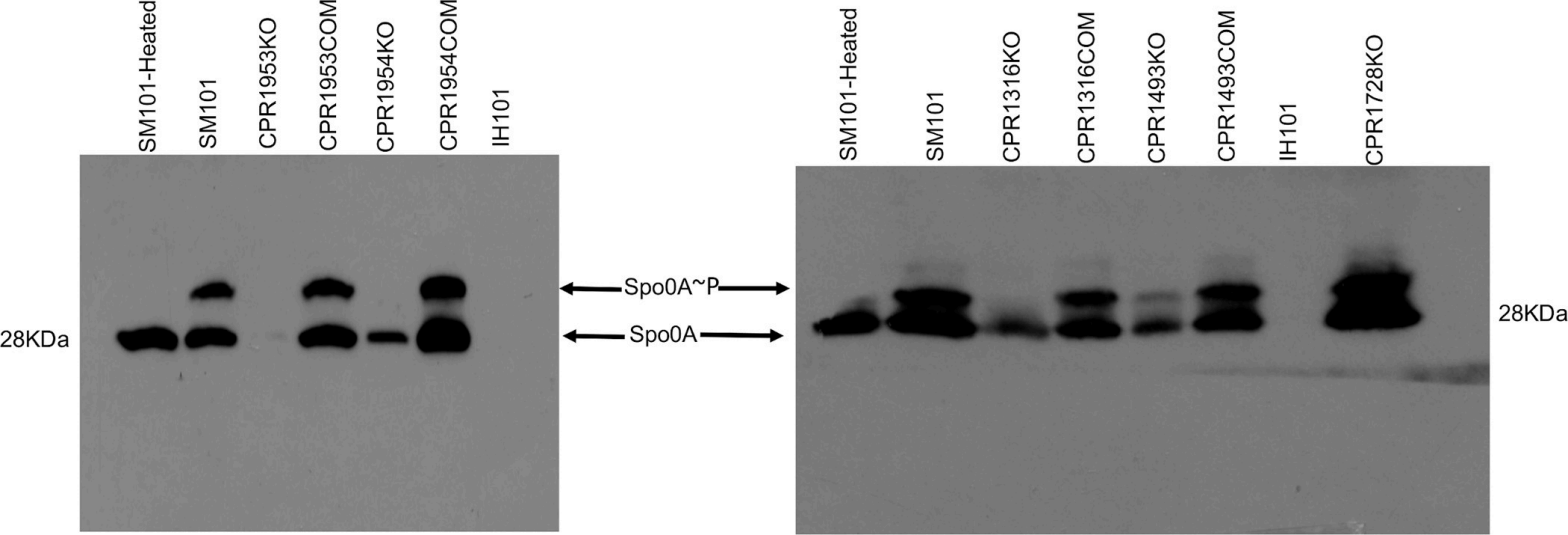
Evaluating the involvement of CPR1728, CPR1316, CPR1493, CPR1953, and CPR1954 in Spo0A phosphorylation in SM101 MDS cultures. The indicated strains were grown for 3 h at 37°C in MDS and an equal amount of cell protein extract for each culture was subjected to Phos-Tag gel for separation of unphosphorylated and phosphorylated Spo0A (Spo0A~P) species. After blotting, a Spo0A Western blot was performed using rabbit polyclonal antiserum raised against *C*. *difficile* Spo0A. The experiment was performed three times and the results shown are representative of three independent repetitions. Loading controls for left and right Western blots are shown in S11 Fig.

Consistent with the Fig 12 results from Western blot analysis of Spo0A production, no Spo0A signal was detected in extracts of 3 h MDS cultures of IH101 or CPR1953KO (Fig 13). Although readily detectable levels of Spo0A protein were present in 3 h MDS CPR1954KO culture lysates, no upper band (representing phosphorylated Spo0A) was visible for extracts of this culture on Phos-Tag Western blots (Fig 13). However, complementation of either CPR1953KO or CPR1954KO restored Spo0A phosphorylation, as detected by these blots (Fig 13). In addition, Phos-Tag Western blotting revealed that CPR1316 and CPR1493 also affect Spo0A phosphorylation in MDS cultures since these Western blots detected reduced levels of Spo0A phosphorylation in extracts of 3 h MDS cultures of CPR1316KO or CPR1493KO mutant strains, while complementation restored normal Spo0A phosphorylation (Fig 13). In contrast, Spo0A phosphorylation was clearly detected for CPR1728KO (Fig 13). These results strongly suggest that CPR1316, CPR1493 and CPR1954 play an important role in Spo0A phosphorylation, as well as Spo0A production, in MDS cultures.

### Evidence that the kinase activity of CPR1316, CPR1493, CPR1953, and CPR1954 is important for their role in sporulation

**Fig 14:**
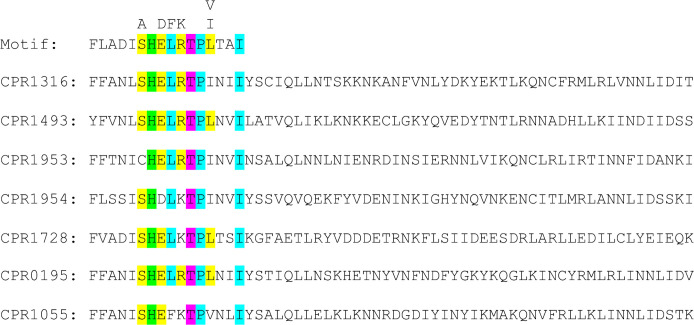
Conservation of the histidine phosphotransfer sequence motif in CPR0195, CPR1055, CPR1316, CPR1493, CPR1728, CPR1953, and CPR1954. The CPR0195, CPR1055, CPR1316, CPR1493, CPR1728, CPR1953, and CPR1954 sequences [23] were aligned and the putative histidine residue involved in phosphotransfer was identified based by sequence alignment analysis of the 100 most diverse HisKA DHp domains [26].

Histidine kinases contain two domains; a dimerization and histidine kinase phosphotransfer domain (DHp) and a catalytic domain [26]. The catalytic domain transfers a phosphoryl group from ATP onto a conserved His residue in the DHp domain, followed by subsequent transfer of that phosphoryl group onto the receiver protein. To determine whether the histidine kinase activity of CPR1316, CPR1493, CPR1953, and CPR1954 is necessary for their role in sporulation, we initially identified the key functional His residue present in the DHp of these four putative histidine kinases by performing a sequence alignment using the conserved histidine phosphorylation site sequence motif [26]. This analysis showed that all four putative kinases contained a classical DHp histidine phosphotransfer site motif, including a conserved His residue (Fig 14).

Using this information, DNA synthesis was performed to create complementing plasmids carrying CPR1316KO, CPR1493KO, CPR1953KO, and CPR1954KO with an alanine substitution for the conserved key functional histidine in the phosphotransfer site. CPR0195KO was excluded from this experiment because it was previously [17] shown that a fragment of CPR0195 can directly phosphorylate Spo0A *in vitro*. Each plasmid was then transformed into its corresponding mutant, creating complementing strains designated as CPR1316COM-H538A, CPR1493COM-H863A, CPR1953COM-H430A, and CPR1954COM-H410A. Creation of the complementing strains was confirmed by PCR using the same internal primers employed earlier for screening orphan histidine kinase genes or their relevant mutant strains, which amplified from these complementing strains a product matching the wild-type product amplified from SM101 DNA (S12A, S13A, S14A, and S15A Figs). To verify complementation of these strains, RT-PCR assays were conducted to evaluate expression of each target gene. Initially, the purity and quality of RNAs were verified as described earlier (S12B, S13B, S14B, and S15B Figs). The RT-PCR assays demonstrated amplification of a product matching the size of wild-type mRNA (no intron insertion) expressed from the *cpr1316*, *cpr1493*, *cpr1953*, or *cpr1954* genes in CPR1316COM-H538A, CPR1493COM-H863A, CPR1953COM-H430A, and CPR1954COM-H410A, respectively (S12B, S13B, S14B, and S15B Figs).

**Fig 15:**
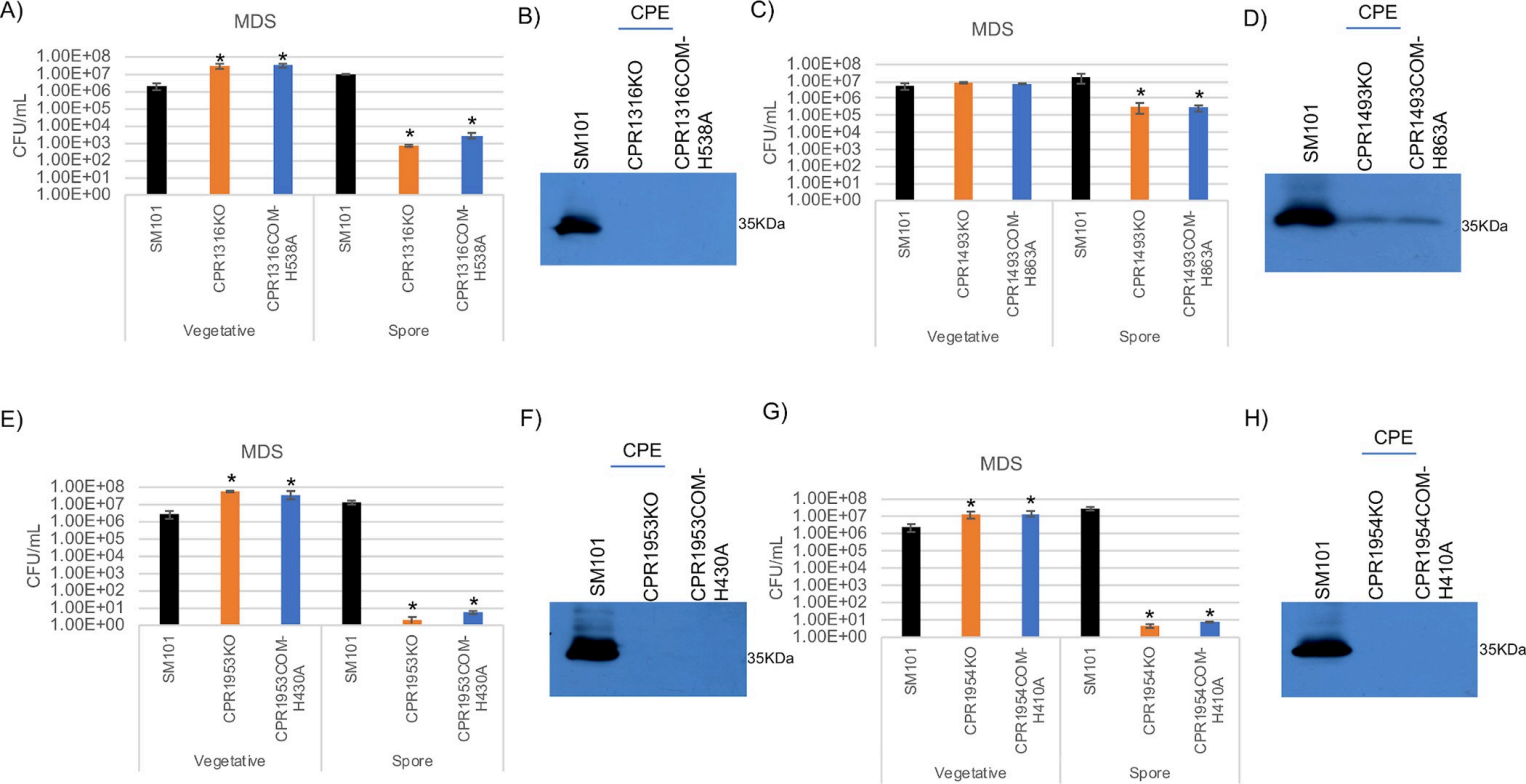
Evaluating the importance of CPR1316, CPR1493, CPR1953, and CPR1954 phosphotransfer activity for vegetative cell viability, sporulation and CPE production when SM101 is cultured in MDS. (A) “Vegetative”, viable vegetative cells (CFU/mL) when SM101, CPR1316KO, and CPR1316COM-H538A were cultured overnight at 37°C in MDS. “Spores”, heat-resistant spores (CFU/mL) in aliquots of those same MDS cultures. (B) SM101, CPR1316KO and CPR1316COM-H538A were cultured overnight at 37°C in MDS and supernatants of those cultures were then subjected to Western blot analysis for CPE toxin production. (C) “Vegetative”, viable vegetative cells (CFU/mL) when SM101, CPR1493KO and CPR1493COM-H863A were cultured overnight at 37°C in MDS. “Spores”, heat-resistant spores (CFU/mL) for those same MDS cultures. (D) SM101, CPR1493KO and CPR1493COM-H863A were cultured overnight at 37°C in MDS and supernatants of those cultures were then subjected to Western blot analysis for CPE toxin production. (E) “Vegetative”, viable vegetative cells (CFU/mL) when SM101, CPR1953KO, and CPR1953COM-H430A were cultured overnight at 37°C in MDS. “Spores”, heat-resistant spores (CFU/mL) in aliquots of those same MDS cultures. (F) SM101, CPR1953KO and CPR1953COM-H430A were cultured overnight at 37°C in MDS and supernatants of those cultures were then subjected to Western blot analysis for CPE toxin production. (G) “Vegetative”, viable vegetative cells (CFU/mL) when SM101, CPR1954KO and CPR1954COM-H410A were cultured overnight at 37°C in MDS. “Spores”, heat-resistant spores (CFU/mL) for those same MDS cultures. (H) SM101, CPR1954KO and CPR1954COM-H410A were cultured overnight at 37°C in MDS and supernatants of those cultures were then subjected to Western blot analysis for CPE toxin production. Results for panels A, C, E, and G are presented as the mean ± SD of three independent experiments. Ordinary one-way analysis of variance (ANOVA; GraphPad Prism 8) was used for statistical analysis of vegetative cell or spore numbers of SM101 and derivatives in panels A, C, E, and G. Asterisk represents *p* < 0.05 versus SM101. Western blots shown in panels B, D, F, and H are representative of three independent experiments. A loading control for this Western blot is presented in S11 Fig.

Wild-type SM101, the putative orphan histidine kinase mutants or the complementing strains expressing the alanine-substituted CPR1316, CPR1493, CPR1953 or CPR1954 variant were cultured overnight in MDS to evaluate the importance of the key functional histidine residue involved in kinase activity for each putative orphan histidine kinase with respect to vegetative cell viability, sporulation and CPE production. As shown in Fig 15, the numbers of viable vegetative cells and heat-resistant spores for CPR1316COM-H538A, CPR1493COM-H863A, CPR1953COM-H430A, and CPR1954COM-H410A were similar to their relevant mutant strains (Fig 15A, 15C, 15E, and 15G). Furthermore, the CPE Western blot analysis indicated that complementation with the alanine-substituted kinases did not restore wild-type production levels for overnight MDS cultures (Fig 15B, 15D, 15F, and 15H). Collectively, these results strongly suggested that phosphotransfer activity is necessary for CPR1316, CPR1493, CPR1953 and CPR1954 to affect sporulation in MDS, supporting the identity of these proteins as histidine kinases.

## Discussion

As mentioned in the Introduction, sporulation is a major contributor to type F diseases, being important for type F strain transmission and required for intestinal CPE production [6]. In the absence of a small animal model that efficiently induces intestinal sporulation and CPE production by type F strains, those processes have been studied to date using several different *C*. *perfringens* laboratory sporulation media, most commonly Duncan-Strong sporulation medium (DS) or MDS [19]. Despite their use for decades, there is only limited understanding of how laboratory sporulation media induce *C*. *perfringens* sporulation or CPE production. Most *C*. *perfringens* laboratory sporulation media contain oligosaccharides, such as starch or raffinose, that are usually considered necessary for inducing sporulation [27], perhaps along with high phosphate levels [28]. Although mechanistic understanding is limited, starch and raffinose are thought to promote sporulation because they are slowly degraded and fermented, which presumably results in limited nutrient availability over time [27,29].

However, *C*. *perfringens* laboratory sporulation media do not closely correspond to the conditions encountered by type F strains when they sporulate in the intestinal lumen during gastrointestinal diseases. For example, DS and MDS sporulation media lack bile salts or intestinal enzymes. Furthermore, these laboratory media induce sporulation using concentrations of oligosaccharides that were arbitrarily chosen without regard to their intestinal relevance. Consequently, these laboratory sporulation media might not faithfully replicate the intestinal signals that induce sporulation during type F disease or reproduce the early sporulation events triggered by those signals.

In the absence of a small animal model for *C*. *perfringens* sporulation and CPE production (see Introduction), this study sought to identify a sporulation-promoting culture condition that better mimics the intestinal conditions present during type F diseases. For this purpose, the ability of diluted mouse small intestinal luminal contents (MIC) to induce sporulation and CPE production by type F strains was evaluated. Results from this evaluation produced the first major finding of this study, i.e., in the presence of Oxyrase supplementation to enhance *C*. *perfringens* survival, MIC can induce significant levels of type F strain sporulation *ex vivo*. When cultured overnight in MIC, type F strains SM101 and F4969 produced 10^5^ to 10^6^ heat-resistant spores/mL. For comparison, a previous survey of fecal spore counts for nine *C*. *perfringens* type F food poisoning outbreaks identified a range from 10^4^ to 10^8^ *C*. *perfringens* spores per gram of feces [30], supporting pathophysiologic relevance for the current MIC sporulation results. Besides inducing sporulation, MIC cultures also exhibited strong CPE production, which is consistent with the sporulation-dependent nature of CPE production [6]. The small volume of intestinal contents in mice represents an acknowledged drawback to their *ex vivo* use for conducting numerous experiments. However, this limitation was partially obviated in the current study by dilution and could be further addressed if future studies demonstrate that intestinal contents from larger animals also induce sporulation and CPE production *ex vivo*.

In *Bacillus subtilis*, sporulation initiates when signals cause multiple kinases to activate a phosphorelay that ultimately phosphorylates Spo0A [31]. Since this phosphorelay is partially or totally missing in clostridia, it is generally accepted that orphan histidine kinases directly phosphorylate Spo0A to initiate clostridial sporulation [17,25,32–36]. Support for this concept, which will be revisited later, has been obtained for two nonpathogenic clostridial species and for *C*. *perfringen*s [17,34,35]. For *C*. *perfringens*, our previous work [17] determined that, relative to wild-type SM101, a SM101 mutant unable to produce the CPR0195 orphan histidine kinase made ~10^4^-fold fewer heat-resistant spores and produced no detectable CPE when cultured in MDS. In contrast, MDS cultures of a SM101 null mutant unable to produce the putative orphan histidine kinase CPR1055 still made wild-type levels of spores and CPE. Those previous findings were confirmed in the current study.

Surprisingly, the current study determined that both the CPR0195 and CPR1055 mutants still produce wild-type levels of heat-resistant spores and CPE when cultured in MIC. This finding provides the second major contribution of the current study, i.e., demonstrating that the importance of an orphan histidine kinase for type F strain sporulation and CPE production can vary using laboratory sporulation media vs. more pathophysiologically-relevant MIC. In *Bacillus* spp., sporulation initiates when several different kinases are activated by specific signals, most notably starvation or bacterial density [16,31]. Similar factors likely also contribute to *C*. *perfringens* sporulation given that, i) limited nutrients provided by poorly-degraded oligosaccharides are thought to contribute to sporulation of type F strains (see above) and ii) previous studies suggest a role for Agr-like quorum sensing in type F strain sporulation [37]. Therefore, the sporulation differences observed for the CPR0195 null mutant in MDS vs MIC may reflect signaling diversity, e.g., a sporulation-promoting signal(s) recognized by CPR0195 may be present when SM101 is cultured in MDS but absent when this strain is cultured in MIC. This additional signal(s) may engage more kinases to enhance sporulation in MDS, as discussed further below. The availability of the new *ex vivo* MIC model should facilitate future studies aimed at identifying disease-related sporulation signals that activate orphan histidine kinases to trigger *C*. *perfringens* sporulation and CPE production under intestinal-like conditions.

Determining that the CPR0195 and CPR1055 null mutants of SM101 still sporulate at wild-type levels in MIC suggested that one or more of the other five annotated orphan histidine kinase of SM101 may be important for initiating sporulation by this strain in MIC. The involvement of other kinases in SM101 sporulation was further suggested by, i) our previous observation [17], confirmed in the current study, that the CPR0195 mutant exhibits only a partial reduction in sporulation when cultured in MDS and ii) BLAST analyses detecting carriage of the genes encoding each of the seven annotated orphan histidine kinases in nearly all other genome-sequenced *C*. *perfringens* strains.

**Fig 16:**
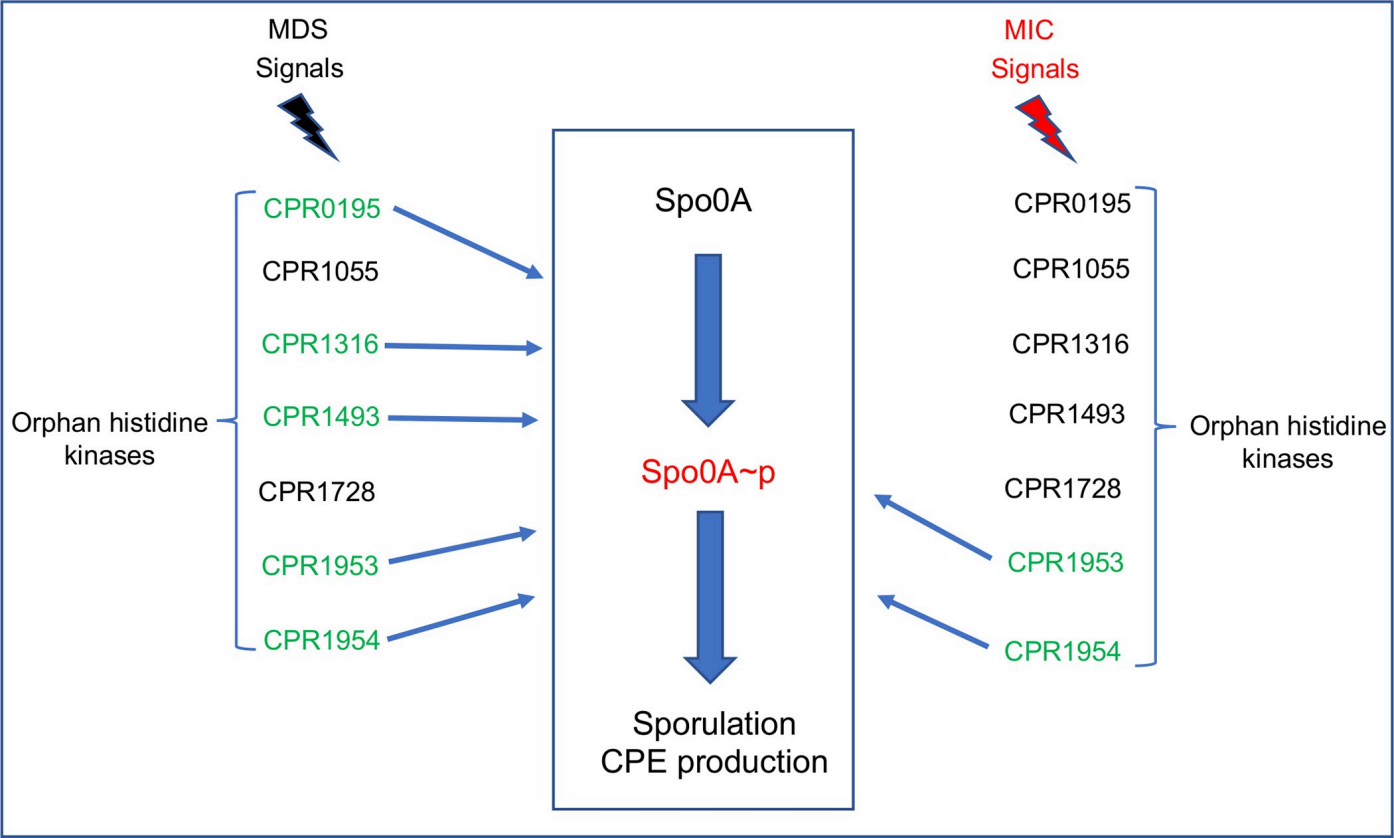
Schematic model for the regulatory pathway that controls sporulation initiation and CPE production in *C*. *perfringens* under two different growth conditions. This figure is drawn based upon the results of this study. The orphan histidine kinases which affect sporulation and CPE production under *in vitro* (MDS) or *ex vivo* (MIC) cultures conditions are colored in green.

Therefore, a series of single SM101 null mutants was constructed that cannot produce one of those remaining putative kinases and each mutant was then tested for sporulation and CPE production when cultured in MDS vs. MIC. Combining the MDS and MIC results for these five newly-constructed mutants with the results for CPR0195 and CPR1055 reveals three phenotypic patterns (Fig 16). CPR1055 and CPR1728 are unnecessary for wild-type levels of sporulation or CPE production when SM101 is cultured in either MDS or MIC. Mutants unable to produce CPR1316, CPR0195 and, to a lesser extent, CPR1493 showed a partial decrease in sporulation and CPE production in MDS, supporting their involvement in sporulation and CPE production in SM101 MDS cultures. However, those mutants still produced wild-type levels of spores and CPE when cultured in MIC, indicating that CPR0195, CPR1316 and CPR1493 play redundant roles, or are unnecessary, for sporulation and CPE production when this strain is cultured in MIC. Further study of this phenotypic group is warranted but the partial reduction in sporulation caused by inactivation of CPR0195, CPR1316, or CPR1493 production in MDS, but not MIC, is consistent with these kinases responding to an additional signal(s) present in MDS, but not MIC, cultures. This extra signaling may boost sporulation to higher levels than achieved using MIC, helping to explain why SM101 forms 1–2 logs more spores in MDS vs. MIC.

Representing the third major finding of the current study, CPR1953 and CPR1954 were shown to be extremely important for sporulation and CPE production whether SM101 was cultured in MDS or MIC. Specifically, inactivating production of either CPR1953 or CPR1954 virtually eliminated sporulation and CPE production in both culture conditions, strongly suggesting a central role for these proteins in both *C*. *perfringens* sporulation and CPE production. RT-PCR indicated that the genes encoding CPR1953 and CPR1954 are present in an operon, so inactivating *cpr1954* conceivably might have also eliminated CPR1953 production. However, both CPR1953 and CPR1954 appear to be important for sporulation and CPE production based upon the following, i) the CPR1953 and CPR1954 null mutants have different phenotypes regarding Spo0A production (further discussion below), ii) complementation of either CPR1953KO or CPR1954KO with a wild-type copy of the inactivated gene (the ORF plus 800 bp of upstream sequence) restores wild-type sporulation and CPE production, and iii) substituting alanine for the key functional His residue in the DHp motif of either kinase virtually eliminates sporulation and CPE production. Since the CPR1954KO mutant still expressed the *cpr1953* gene, this gene appears to have an independent promoter, as well as being expressed in an operon, but regulation of expression of these kinase genes requires further study.

The current results establish that multiple orphan histidine kinases can impact *C*. *perfringens* sporulation but not all putative orphan histidine kinases are necessary for *C*. *perfringens* sporulation (at least under the tested incubation conditions), which is reminiscent of results reported for *Clostridium acetobutylicum* and *Clostridium thermocellum*, both nonpathogenic species [34,35]. Specifically, three of the five orphan histidine kinases of *C*. *acetobutylicum* were shown to be important for sporulation, while three of six orphan histidine kinases of *C*. *thermocellum* were found to impact sporulation. In *C*. *acetobutylicum*, single kinase deletions produced a relatively modest decrease in sporulation, while inactivation of genes encoding either of two kinases in *C*. *thermocellum* essentially abolished sporulation, which is similar to the effects of inactivating the genes encoding CPR1953 or CPR1954 in SM101. BLAST analyses to date have not identified extensive similarity between the kinases impacting sporulation in those nonpathogenic clostridial species vs *C*. *perfringens*, which is consistent with the expectation of different signals initiating sporulation for *C*. *perfringens*, which commonly sporulates in the host, vs nonpathogenic clostridia like *C*. *acetobutylicum* or *C*. *thermocellum* that are not typically present inside hosts. Interestingly, those BLAST analyses indicated that *Clostridium novyi*, another human and animal pathogen, does carry kinases with significant similarity to the kinases capable of impacting *C*. *perfringens* sporulation in MDS, i.e., CPR0195, CPR1316, CPR1493, CPR1953 and CPR1954. It is possible that those kinases also contribute to *C*. *novyi* sporulation, which should be examined in the future. BLAST searches did not identify any kinases with obvious similarity to the orphan histidine kinases of *Clostridioides difficile*, where kinase contributions to initiating sporulation remain unclear [25].

A fourth contribution of the current study is to begin identifying mechanisms by which CPR1316, CPR1493, CPR1953 and CPR1954 contribute to sporulation in MDS (CPR1316 and CPR1493) or in both MDS and MIC (CPR1953 and CPR1954). Using a *sigF*-driven reporter plasmid, it was previously shown [17] that the CPR0195 kinase acts early during sporulation in MDS, i.e., before *sigF* expression, which is consistent with this orphan histidine kinase functioning at the initiation of sporulation. By the same approach, the current study determined that the orphan histidine kinase genes encoding CPR1316, CPR1493, CPR1953 and CPR1954 also act prior to *sigF* expression in MDS cultures of SM101, i.e., they also function early during sporulation.

In *Bacillus subtilis*, Spo0A phosphorylation causes activation of a feedback loop that increases Spo0A production [38]. Similarly, a previous study showed that inactivating CPR0195 production modestly decreases Spo0A production when SM101 is cultured in MDS [17]. The current study determined that inactivating CPR1316 or CPR1493 production also modestly decreases Spo0A production levels for 3 h MDS cultures of SM101. Under the same culture conditions, the *cpr1954* null mutant made substantially less Spo0A than wild-type SM101, while the *cpr1953* null mutant produced no detectable Spo0A. Notably, complementation restored wild-type Spo0A production levels to all mutants. When the incubation in MDS was extended to 5 h, all mutants produced approximately wild-type levels of Spo0A, with the exception of the *cpr1953* mutant which still produced little or no Spo0A. These results suggest that one contribution of CPR1953 to sporulation is a long-term influence on Spo0A production, at least in MDS. Spo0A Western blots could not be performed on MIC cultures because they would require using prohibitive numbers of mice, so qRT-PCR was utilized instead. Results of that experiment also detected decreased amounts of *cpr1953* and *cpr1954* expression, in agreement with the Western blot results for MDS cultures.

As mentioned in the Introduction, it is generally believed that sporulation in the clostridia, including *C*. *perfringens*, begins when Spo0A is phosphorylated by orphan histidine kinases. The current study used Phos-Tag Spo0A Western blots to evaluate the involvement of each putative or proven orphan histidine kinase in Spo0A phosphorylation in MDS cultures. Those blots provided clear evidence for Spo0A phosphorylation in MDS culture lysates of wild-type SM101, i.e., two bands were visible on these blots and the upper band disappeared with heating, consistent with the expected heat-induced removal of phosphate from Spo0A. MDS lysates of CPR1728KO also showed strong Spo0A phosphorylation, consistent with this kinase being unnecessary for SM101 sporulation under this incubation condition, as discussed earlier. As expected from the classical Spo0A Western blot results, no Spo0A signal was detected in MDS lysates of the CPR1953KO mutant on Phos-Tag Spo0A Western blots. Phos-Tag blots of lysates from MDS cultures of CPR1493KO or CPR1316KO mutants detected a strong Spo0A signal but the amount of phosphorylated Spo0A was reduced, consistent with the plate count results showing a partial reduction in sporulation for these mutants when grown in MDS. The most interesting finding from the Phos-Tag experiments concerned the results for extracts of CPR1954KO MDS cultures. While the Spo0A signal for these samples was reduced, consistent with the classical Spo0A Western blot results, there was no evidence of Spo0A phosphorylation for the Spo0A that was produced. Collectively these results suggest that CPR1953 and CPR1954 are so impactful for sporulation because, i) CPR1953 is important for Spo0A production and ii) CPR1954 is important for both Spo0A production and Spo0A phosphorylation.

In a previous study [17] we showed that a fragment of CPR0195 can directly phosphorylate Spo0A *in vitro*. Using portions of kinases for *in vitro* Spo0A phosphorylation assays is often necessary [35] because many of these proteins, probably including all *C*. *perfringens* orphan histidine kinases except CPR1055, are membrane proteins that are insoluble when expressed recombinantly. The current study attempted to perform a similar study with a CPR1954 kinase domain to show that portions of this kinase can phosphorylate Spo0A *in vitro* but those efforts have been unsuccessful to date. While efforts to perform this experiment will continue, it is also noteworthy that results of such *in vitro* phosphorylation experiments can be complicated to interpret regarding the role of a kinase in sporulation, e.g., a *C*. *difficile* kinase was shown to phosphorylate Spo0A *in vitro* but a mutant unable to produce that kinase still sporulated at wild-type levels [36,39].

The current study did show that alanine substitution for the key functional His residue in the DHp domain resulted in the loss of sporulation and CPE production by CPR1316, CPR1493, CPR1953 and CPR1954, which supports the identity of these four proteins as histidine kinases. Those results also support the importance of phosphotransfer activity for the effects of these proteins on sporulation when SM101 is cultured in MDS.

Last, it was observed that the effects of all tested *C*. *perfringens* orphan histidine kinases on CPE production correlate closely with their effects on sporulation. For example, CPR1493 caused a weak reduction in both sporulation and CPE production. Therefore, the current study obtained no evidence for an orphan histidine kinase playing a specific role in CPE production rather than having a general impact on sporulation.

In summary, the current study provides several seminal insights into the involvement of orphan histidine kinases in type F strain sporulation and CPE production. However, many questions remain for future studies. For example, what is the signal(s) triggering sporulation in MIC? Is this signal also present in MDS? How do kinases recognize this signal(s)? What are the interrelationships between the different kinases during sporulation in MDS? In MIC?

## Materials and methods

### Bacterial strains, plasmids, media, and growth condition

Bacterial strains and plasmids used in this study are listed and described in Table 1. Stock cultures of both wild-type and complemented *C*. *perfringens* isolates were prepared in cooked meat medium (CMM; Oxoid) stored at -20°C. *C*. *perfringens* mutant strains and *E*. *coli* TOP10 cells harboring pUC57-Kan plasmid were maintained in 35% glycerol stock cultures stored at -80°C. All *C*. *perfringens* isolates were cultured at 37°C under anaerobic condition using a GasPak EZ anaerobe container system. The following broth media were used for vegetative growth of *C*. *perfringens*: Fluid thioglycollate medium (FTG; Difco Laboratories), and TGY broth (3% tryptic soy broth [Becton, Dickinson], 2% Glucose [Fisher Scientific], 1% yeast extract [Becton, Dickinson], and 0.1% sodium thioglycollate [Sigma-Aldrich]). Modified Duncan-Strong medium (MDS) [19] was used as a sporulation medium for broth cultures and consisted of 1.5% proteose peptone [Becton, Dickinson], 0.4% yeast extract [Becton, Dickinson], 1% disodium phosphate heptahydrate [Fisher Scientific], 0.1% sodium thioglycollate [Sigma-Aldrich], 0.4% raffinose [Sigma-Aldrich], and 1mM caffeine [Sigma-Aldrich]). Brain heart infusion (BHI) agar (Research Products International) with or without supplementation with chloramphenicol (Cm; 15 μg/mL) (Sigma-Aldrich) was utilized for mutant selection and counting spores and vegetative cells. Luria-Bertani (LB) medium (Fisher Scientific) supplemented with kanamycin (Kan; 100 μg/mL) (Sigma-Aldrich) was utilized for growth of *E*. *coli* TOP10 cells harboring pUC57-Kan plasmid.

**Table 1:** Strains and plasmids used in this study.

Strains or plasmids	Description	Reference
**Strains**		
TOP10	*E*. *Coli-*Competent cells	Invitrogen
SM101	Transformable derivative of a type F food poisoning strain	[13]
Strain 13	Soil isolate	[40]
F4969	Sporadic diarrhea type F isolate from Europe	[19]
CPR0195KO	SM101 *cpr0195* null mutant	[17]
CPR1055KO	SM101 *cpr1055* null mutant	[17]
CPR1728KO	SM101 *cpr1728* null mutant	This study
CPR1316KO	SM101 *cpr1316* null mutant	This study
CPR1493KO	SM101 *cpr1493* null mutant	This study
CPR1953KO	SM101 *cpr1953* null mutant	This study
CPR1954KO	SM101 *cpr1954* null mutant	This study
CPR1316COM	SM101 *cpr1316* null mutant complemented with *cpr1316*	This study
CPR1493COM	SM101 *cpr1493* null mutant complemented with *cpr1493*	This study
CPR1953COM	SM101 *cpr1953* null mutant complemented with *cpr1953*	This study
CPR1954COM	SM101 *cpr1954* null mutant complemented with *cpr1954*	This study
CPR1316COM-H538A	SM101 *cpr1316* null mutant complemented with *cpr1316-*H538A	This study
CPR1493COM-H863A	SM101 *cpr1493* null mutant complemented with *cpr1493*-H863A	This study
CPR1953COM-H430A	SM101 *cpr1953* null mutant complemented with *cpr1953*-H430A	This study
CPR1954COM-H410A	SM101 *cpr1954* null mutant complemented with *cpr1954*-H410A	This study
SM101 (pJIR750)	Wild-type SM101 with a empty vector as a negative control	[24]
SM101 (pJIR750-P*sigF*-*gusA*)	Wild-type SM101 with a SigF promoter and GusA vector	[24]
CPR1728KO (pJIR750-P*sigF*-*gusA*)	CPR1728KO with a SigF promoter and GusA vector	This study
CPR1316KO (pJIR750-P*sigF*-*gusA*)	CPR1316KO with a SigF promoter and GusA vector	This study
CPR1493KO (pJIR750-P*sigF*-*gusA*)	CPR1493KO with a SigF promoter and GusA vector	This study
CPR1953KO (pJIR750-P*sigF*-*gusA*)	CPR1953KO with a SigF promoter and GusA vector	This study
CPR1954KO (pJIR750-P*sigF*-*gusA*)	CPR1954KO with a SigF promoter and GusA vector	This study
**Plasmids**		
pJIR750ai	*C*. *perfringens*-*E*. *coli* shuttle plasmid for TargeTron gene knockout system	[20]
pJIR750*cpr1728i*	Construction of *cpr1728* null mutant	This study
pJIR750*cpr1316*i	Construction of *cpr1316* null mutant	This study
pJIR750*cpr1493*i	Construction of *cpr1493* null mutant	This study
pJIR750*cpr1953*i	Construction of *cpr1953* null mutant	This study
pJIR750*cpr1954*i	Construction of *cpr1954* null mutant	This study
pJIR750	*C*. *perfringens*-*E*. *coli* shuttle plasmid	[22]
pJIR750-P*sigF*-*gusA*	Construction of GusA vector with SigF promoter	[24]
pJIR750-*cpr1316COM*	Used for construction of a CPR1316COM plasmid	This study
pJIR750-*cpr1493COM*	Used for construction of a CPR1493COM plasmid	This study
pJIR750-*cpr1953COM*	Used for construction of a CPR1953COM plasmid	This study
pJIR750-*cpr1954COM*	Used for construction of a CPR1954COM plasmid	This study
pJIR750-*cpr1316COM*-H538A	Used for construction of a CPR1316COM-H538A plasmid	This study
pJIR750-*cpr1493COM*-H863A	Used for construction of a CPR1493COM-H863A plasmid	This study
pJIR750-*cpr1953COM-*H430A	Used for construction of a CPR1953COM-H430A plasmid	This study
pJIR750-*cpr1954COM*-H410A	Used for construction of a CPR1954COM-H410A plasmid	This study
pUC57	*E*. *coli* cloning vector	GenScript
pUC57-*cpr1316COM*-H538A	Used for construction of a CPR1316COM-H538A plasmid	This study
pUC57-*cpr1493COM*-H863A	Used for construction of a CPR1493COM-H863A plasmid	This study
pUC57-*cpr1953COM-*H430A	Used for construction of a CPR1953COM-H430A plasmid	This study
pUC57-*cpr1954COM*-H410A	Used for construction of a CPR1954COM-H410A plasmid	This study

### Construction of SM101 null mutants and complementing strains

Five annotated orphan histidine kinase genes (*cpr1728*, *cpr1316*, *cpr1493*, *cpr1953* and *cpr1954*) in SM101 [13] were individually inactivated by insertion of a targeted group II intron using the *Clostridium*-modified TargeTron knockout system [20]. For this purpose, a 350-bp intron specifically targeting one of the above-mentioned genes were prepared by PCR using the pACD4K-C plasmid (Sigma-Aldrich) as the template and primers listed in Table 2. The resultant PCR product for each gene was then ligated into pJIR750ai plasmid between the *BsrGI* and *HindIII* restriction sites to construct the following vectors: pJIR750*cpr1728*i, pJIR750*cpr1316*i, pJIR750*cpr1493*i, pJIR750*cpr1953*i, and pJIR750*cpr1954*i. To create *cpr1728*, *cpr1316*, *cpr1493*, *cpr1953*, and *cpr1954* single null mutant strains, the resultant intron-carrying vectors were electroporated into SM101 and transformants were selected by plating onto BHI agar plates containing 15 μg/mL of chloramphenicol. Each putative single mutant strain was selected and its mutation confirmed by colony PCR using screening primer sets listed in Table 2. The intron-carrying plasmid was then cured from each single null mutant, as described previously [20]. These mutant strains were named CPR1728KO, CPR1316KO, CPR1493KO, CPR1953KO, and CPR1954KO. Each mutant was then confirmed by PCR, RT-PCR, and Southern blotting analyses as described later.

**Table 2:** Primers used in this study.

Primer name	Purpose	Sequence^a^	PCR product size (bp)
1728–891|892s-IBS	pJIR750cpr1728i construction	AAAAAAGCTTATAATTATCCTTAGTATTCAAGGCCGTGCGCCCAGATAGGGTG	350
1728–891|892s-EBS1d		CAGATTGTACAAATGTGGTGATAACAGATAAGTCAAGGCCTTTAACTTACCTTTCTTTGT	
1728–891|892s-EBS2		TGAACGCAAGTTTCTAATTTCGATTAATACTCGATAGAGGAAAGTGTCT	
EBS universal		CGAAATTAGAAACTT GCG TTCAGTAAAC	
Target site		TATATAACTGATAAAGAGGTATTAAAGGC**CT**TTTTTGATGAAAAA	
1728KO-F	Screen for intron insertion in *cpr1728*; *cpr1728* RT-PCR	TAGGAAAAAGCTTTAATTATATGGC	Wild-type:(275); Mutant:(1175)
1728KO-R		AACTTCAATTTCAACCCTATC	
1316–870|871s-IBS	pJIR750cpr1316i construction	AAAAAAGCTTATAATTATCCTTAAGTGACGTTGTAGTGCGCCCAGATAGGGTG	350
1316–870|871s-EBS1d		CAGATTGTACAAATGTGGTGATAACAGATAAGTCGTTGTAGTTAACTTACCTTTCTTTGT	
1316–870|871s-EBS2		TGAACGCAAGTTTCTAATTTCGATTTCACTTCGATAGAGGAAAGTGTCT	
EBS universal		CGAAATTAGAAACTT GCG TTCAGTAAAC	
Target site		ATAGAATTTGATTGCAATAGTGAAGTTGT**AG**TACTTAGTAACAAT	
1316KO-F	Screen for intron insertion in *cpr1316*; *cpr1316* RT-PCR; Screen for CPR1316COM and CPR1316COM-H538A	GTATTTGGTATGTTTTTTGATTTAC	Wild-type:(349); Mutant:(1249)
1316KO-R		TCTTTTGAAGATTAACCTTTACAAC	
1493–415|416s-IBS	pJIR750cpr1493i construction	AAAAAAGCTTATAATTATCCTTAAGCTACCAAATAGTGCGCCCAGATAGGGTG	350
1493–415|416s-EBS1d		CAGATTGTACAAATGTGGTGATAACAGATAAGTCCAAATAGTTAACTTACCTTTCTTTGT	
1493–415|416s-EBS2		TGAACGCAAGTTTCTAATTTCGATTTAGCTTCGATAGAGGAAAGTGTCT	
EBS universal		CGAAATTAGAAACTT GCG TTCAGTAAAC	
Target site		ATGAAGATGGAAAAAGCAAGCTATCAAAT**AG**TAATATATGGGATA	
1493KO-F	Screen for intron insertion in *cpr1493*; *cpr1493* RT-PCR; Screen for CPR1493COM and CPR1493COM-H863A	TATCAGCTTTAATAAAATATGACAA	Wild-type:(350); Mutant:(1250)
1493KO-R		ATTAATTACACTTAACCCCTCACTT	
1953–62|63a-IBS	pJIR750cpr1953i construction	AAAAAAGCTTATAATTATCCTTAATTATCGATATGGTGCGCCCAGATAGGGTG	350
1953–62|63a-EBS1d		CAGATTGTACAAATGTGGTGATAACAGATAAGTCGATATGTATAACTTACCTTTCTTTGT	
1953–62|63a-EBS2		TGAACGCAAGTTTCTAATTTCGGTTATAATCCGATAGAGGAAAGTGTCT	
EBS universal		CGAAATTAGAAACTT GCG TTCAGTAAAC	
Target site		ATTAAGAATAATAT**AC**ATATCAATAATATTATCATCTATAATATT	
1953KO-F	Screen for intron insertion in *cpr1953*; *cpr1953* RT-PCR; Screen for CPR1953COM and CPR1953COM-H430A	GTGGAATACAAAGTGGAATATGA	Wild-type:(365); Mutant:(1265)
1953KO-R		CCTAATGCTATAACAAATACAATAA	
1954–583|584a-IBS	pJIR750cpr1954i construction	AAAAAAGCTTATAATTATCCTTATATATCCTAGAGGTGCGCCCAGATAGGGTG	350
1954–583|584a-EBS1d		CAGATTGTACAAATGTGGTGATAACAGATAAGTCCTAGAGCATAACTTACCTTTCTTTGT	
1954–583|584a -EBS2		TGAACGCAAGTTTCTAATTTCGGTTATATATCGATAGAGGAAAGTGTCT	
EBS universal		CGAAATTAGAAACTT GCG TTCAGTAAAC	
Target site		TAGTATTAATATAT**GC**TCTAGTATATATTTTTAATTTAGAAGCTT	
1954KO-F	Screen for intron insertion in *cpr1954*; *cpr1954* RT-PCR; Screen for CPR1954COM and CPR1954COM-H410A	GATGTAATTGTGTTTGGGGTGAT	Wild-type:(596); Mutant:(1496)
1954KO-R		CCTGAAGAATATGAAGCTTCTCC	
1316COM-F	pJIR750cpr1316COM construction	CCGCgtcgacCAAATTGAAATAACATAATAGG (*SalI*)^b^	3464
1316COM-R		CCGCctgcagTAGTACTAATGATTCTAGTAAAC (*PstI*)^b^	
1493COM-F	pJIR750cpr1493COM construction	CGCgtcgacGCAAGGTTACTACTATGCTAGT (*SalI*)^b^	4367
1493COM-R		CCGCctgcagTCCTCTTAATATTAAATCATTC (*PstI*)^b^	
1953COM-F	pJIR750cpr1953COM construction	CCCGggatccTGGATAAAGATTTTATAGAAAAAGC (*BamHI*)^b^	2354
1953COM-R		CGTActgcagCCTCCACCTAAAGTTATTAACAAAC (*PstI*)^b^	
1954COM-F	pJIR750cpr1954COM construction	CCGCgtcgacTAGCAAATGCATTAGATAATG (*SalI*)^b^	2893
1954COM-R		CCGCctgcagCCCTATTATTTTATAAACTATC (*PstI*)^b^	
polC-F	PCR analysis of *polC*	ACTTCCCTGCAAGCCTCTTCTCCT	300
polC-R		TGGTTCAGCTTGTGAAGCAGGGC	
16S-F	RT-PCR analysis of *16S*	CCTTACCTACACTTGACATCCC	118
16S-R		GGACTTAACCCAACATCTCACG	
Spo0A-F	qRT-PCR analysis of *spo0A*	TGAGGATAACAAAGAGCCTATGG	73
Spo0A-R		ATGTGAGCTGGTACTCCTATTTC	
PJIR750-F2	pJIR750cpr1953COM screening	GTTGGCCGATTCATTAATGCA	642
cpr1953-R2		GAATAAGAAGCCAAAATATCCCTA	
PJIR750-F	pJIR750cpr1954COM screening	CACAGGAAACAGCTATGACC	1796
1954KO-R		CCTGAAGAATATGAAGCTTCTCC	

^a^ Bold nucleotides with underline indicate the position of intron insertion into each orphan histidine kinase to generate each mutant.

^b^ Restriction site sequences are shown in lower case.

When a mutant showed reduced sporulation (see Results), it was complemented to address the possibility of secondary mutations or polar effects. Complementing strains named CPR1316COM, CPR1493COM, CPR1953COM, and CPR1954COM were constructed using two specific primers (see Table 2) to amplify, from SM101 DNA, the entire ORF (open reading frame) and approximately 800 bp of upstream sequences for *cpr1316*, *cpr1493*, *cpr1953*, and *cpr1954*. Each PCR product was then ligated into the *C*. *perfringens/E*. *coli* shuttle plasmid pJIR750 [22] between appropriate restriction sites (see Table 2). Finally, each mutant was complemented by electroporation of the corresponding plasmid. The complemented strains were selected by growth on BHI agar plates supplemented with 15 μg/mL of chloramphenicol, and then confirmed by PCR, RT-PCR, and Western blot analyses.

### Identification of the key functional his residue in the DHp domain and construction of plasmids for alanine substitutions of that his residue

The DHp domain of CPR0195, CPR1055, CPR1316, CPR1493, CPR1728, CPR1953, and CPR1954 were predicted using the InterPro software (http://www.ebi.ac.uk/interpro/). Sequences in those predicted DHp domains were then aligned to identify the putative key functional His residue involved in phosphotransfer [26]. Thereafter, DNA synthesis was performed by GenScript to create constructs containing the entire ORF, and approximately 800 bp of upstream sequences, for *cpr1316*, *cpr1493*, *cpr1953*, and *cpr1954* except that the identified putative key functional His residue involved in phosphotransfer was substituted to alanine in these constructs. The synthesized sequences were then cloned into pUC57-Kan vector between the *BamHI* and *Sall* restriction sites. The resultant plasmids were transformed into *E*. *coli* TOP10 competent cells (Invitrogen) and sequenced to confirm their identity. Next, the constructs were purified and digested with appropriate restriction enzymes for cloning into the pJIR750 *C*. *perfringens*-*E*. *coli* shuttle plasmid [22]. The resulting pJIR750 plasmids were then electroporated into CPR1316KO, CPR1493KO, CPR1953KO, or CPR1954KO, e.g., a pJIR750 plasmid carrying the gene encoding an alanine-substituted CPR1316 was electroporated into the CPR1316KO mutant. The complemented strains were selected by growth on BHI agar plates supplemented with 15 μg/mL of chloramphenicol, and then confirmed by PCR using appropriate primer sets (Table 2). The resultant complementing strains named CPR1316COM-H538A, CPR1493COM-H863A, CPR1953COM-H430A, and CPR1954COM-H410A. Each complementing strain was then confirmed by PCR and RT-PCR analyses as described later.

### Use of mouse small intestinal contents in an *ex-vivo* model

Small intestinal contents were removed from healthy ~4 months old male or female BALB/c mice that had been euthanized by CO_2_ asphyxiation for colony management (IACUC protocol 21850). These small intestine luminal contents were immediately harvested and stored at -80°C until use.

Mouse intestine luminal contents were thawed and pooled on the day of experiment. The pooled contents were then diluted (1:1) with sterile PBS (phosphate buffer saline, Corning). After mixing the intestinal contents with PBS, solid material was removed by centrifugation at 15,000 x g for 10 min. The resultant supernatant was further diluted with PBS (1:5) and then filtered through a 0.2 μm syringe filter for sterilization. To ensure that the filtered diluted intestinal contents were sterile, those supernatants were spotted onto a BHI agar plate and incubated aerobically or anaerobically at 37°C overnight.

For experiments, a 40 μl aliquot of overnight FTG culture of a *C*. *perfringens* isolate was inoculated into 1 mL of sterile diluted mice intestinal contents (MIC) containing 5% Oxyrase (to obtain low redox conditions) (Oxyrase, Inc) and incubated overnight (18 h) or other specific time points anaerobically at 37°C.

### Western blot analyses for CPE production

To evaluate CPE production by wild-type SM101 and its derivative mutants or complemented strains, these strains were cultured in FTG broth for 16 h at 37°C. A 0.2 mL or 40 μl aliquots of each overnight FTG culture was then added to 10 mL of fresh MDS broth or 1 mL of MIC containing 5% Oxyrase, respectively. After incubation overnight at 37°C to allow sporulation and mother cell lysis, each MDS or MIC culture was adjusted to equal optical density at 600 nm (OD_600_) using a Bio-Rad SmartSpec spectrophotometer. Subsequently, equal volumes of those adjusted cultures were centrifuged and equal volumes of the resultant supernatants were mixed with 5x sodium dodecyl sulfate (SDS) loading buffer. Those samples were then electrophoresed on an SDS-containing 12% polyacrylamide gel. Separated proteins were transferred onto a polyvinylidene difluoride (PVDF) membrane (Bio-Rad) and the blots were blocked for 1 h with Tris-buffered saline with Tween 20 (0.05% vol/vol) containing 5% nonfat dry milk. The blots were then incubated with a 1:5,000 dilution of rabbit polyclonal CPE antibody [41] overnight at 4°C. This was followed by incubation of the blots with a 1:10,000 dilution of horseradish peroxidase-conjugated secondary anti-rabbit antibody (Sigma-Aldrich) for 1 h at room temperature. After washing, the blots were developed with SuperSignal West Pico Chemiluminescent substrate (Fisher Scientific) and exposure to X-ray film (Life Science Products).

To demonstrate loading of equal levels of total proteins for all samples in Western blotting analyses, the same PVDF membranes were stained with either Coomassie Brilliant blue G250 (Sigma-Aldrich) or Swift membrane stain kit after Western blot experiments, according to the manufacturer’s instructions.

### Evaluation of Spo0A production by Western blotting or qRT-PCR analysis

To evaluate Spo0A production by wild-type SM101 and its mutant or complemented strains cultured in MDS, the strains were first cultured in FTG broth overnight at 37°C. A 0.2 mL aliquot of each FTG culture was then transferred into 10 mL of MDS medium for 3 h or 5 h at 37°C. Subsequently, each MDS culture was adjusted to equal optical density at 600 nm (OD_600_) using a Bio-Rad SmartSpec spectrophotometer, followed by centrifugation of equal volumes of those adjusted cultures. The pelleted cells were then lysed with B-PER reagent (Thermo Scientific) containing protease inhibitor cocktail II (Research Products International). After boiling for 5 min at 95°C, lysates were subjected to Western blot analyses using a 1:2,000 dilution of rabbit polyclonal antiserum raised against *Clostridioides difficile* Spo0A, which was kindly provided by Dr. Aimee Shen, Tufts University. The Western blots were processed as described above for CPE Western blots. A previously constructed [10] *spo0A* null mutant (IH101) of SM101 was used as a negative control in this experiment.

To compare *spo0A* expression levels in MIC, SM101, CPR1953KO, CPR1954KO, CPR1953COM and CPR1954COM were cultured in MIC with 5% Oxyrase for 3 h at 37°C. RNAs were then purified from these samples as described below (see RNA isolation, RT-PCR, and qRT-PCR analyses section) and subjected to qRT-PCR using specific primers pairs for *spo0A* gene (see Table 2).

### Phos-Tag gel electrophoresis and Western blot analyses of Spo0A

For this experiment, a Spo0A phosphorylation assay was performed as previously described, with slight modification [25]. Briefly, 0.2 mL aliquots of overnight FTG culture of wild-type SM101 and its derivative mutant or complemented strains were inoculated into 10 mL of fresh MDS. After a 3 h incubation at 37°C, cells were harvested by centrifugation and pelleted cells were lysed at 4°C with B-PER reagent (Thermo Scientific) containing protease inhibitor cocktail II and phosphatase inhibitor cocktail II (Sigma-Aldrich). Total protein in those lysates was quantified using the Pierce BCA protein assay kit (Thermo Scientific). Approximately 15 μg total protein of those lysates were mixed with 5x SDS loading buffer without EDTA and then electrophoresed on a 12% Phos-Tag SDS-PAGE gel (Fujifilm Wako Chemicals) at 110 V for 4.5 h at 4°C. Separated proteins were transferred onto a nitrocellulose membrane (Bio-Rad) overnight at 30 V at 4°C in transfer buffer containing 10% methanol and 0.04% SDS. The remainder of the experiment was processed as described earlier for Spo0A Western blot analyses. In this experiment, lysate from a *spo0A* mutant strain (IH101) or heated lysate from wild-type SM101, without any heat-labile phosphoryl groups of Spo0A, were used as controls.

### DNA extraction, PCR, and Southern blot analyses

Genomic DNA was isolated from SM101 and its isogenic mutant or complemented strains using the MasterPure Gram-positive bacterial DNA purification kit (Epicentre). All constructed plasmid DNAs were extracted from *C*. *perfringens* Strain 13 (cloning host strain) using a QIAprep Spin Miniprep Kit (Qiagen), as previously described [42] before electroporation to appropriate SM101 mutant strains.

PCR reactions for confirming mutant strains or transformants were performed using appropriate primer sets listed in Table 2 and 2x Dream*Taq* Green PCR Master Mix (ThermoFisher Scientific). The following parameters were used for PCR amplification: (1) 95°C for 5 min; (2) 35 cycles of 95°C for 30 sec, 55°C for 30 sec, 72°C for 90 sec, and (3) a final extension for 5 min at 72°C.

LongAmp *Taq* polymerase (New England Biolabs) was used to prepare PCR products for making complementing strains as well as *cpr1954*-*cpr1953* PCR (operon PCR assay), and the following parameters was used for those PCR amplifications: (1) 95°C for 5 min; (2) 35 cycles of 95°C for 30 sec, 55°C for 30 sec, 65°C for 50 sec per Kb, and (3) a final extension for 10 min at 65°C. Finally, 20 μl of each PCR reaction was electrophoresed on a 2% agarose gel and visualized using ethidium bromide stain.

To demonstrate the presence of a single intron insertion in each kinase null mutant, Southern blot analyses were performed as previously described [43]. Briefly, 3 μg of highly-purified genomic DNA from wild-type SM101 and each derivative mutant was digested using *EcoRI* restriction enzyme (New England Biolabs) overnight at 37°C, electrophoresed on a 1% agarose gel, and then alkali transferred onto a positively charged nylon membrane (Roche). Following hybridization of membranes with an intron-specific probe [43], the digoxigenin-labeled hybridized intron probe was detected using CSPD substrate (Roche) according to the manufacturer’s instruction.

### RNA isolation, RT-PCR, and qRT-PCR analyses

For RNA isolation, *C*. *perfringens* cells were grown overnight in FTG broth medium at 37°C. Aliquots (0.2 mL) of each FTG culture were then inoculated into 10 mL of freshly prepared MDS broth medium. After a 2 h or 3 h incubation at 37°C, cells were collected and RNA was extracted using the saturated phenol (Fisher Scientific) method, as previously described [44]. Extracted RNA was then stored at -80°C. The concentration of extracted RNA was determined by A_260_ measurements using a NanoDrop 100 spectrophotometer (Thermo Scientific). The quality and purity of each isolated RNA was also assessed by performing PCR for *16S* and *polC* genes in the presence or absence of reverse transcriptase enzyme.

RT-PCR analyses of gene expression were conducted using one-step RT-PCR containing 200 ng of purified RNA, 2x Dream*Taq* Green PCR Master Mix (ThermoFisher Scientific), avian myeloblastosis virus (AMV) reverse transcriptase (4 U; Promega), milliQ water, and primers specific for the *cpr1728*, *cpr1316*, *cpr1493*, *cpr1953*, *cpr1954*, or *16S* genes. All primers used in this experiment are listed in Table 2. The following parameters were used for RT-PCR: (1) 95°C for 4 min; (2) 42°C for 45 min (cDNA synthesis); (3) 95°C for 2 min; (4) 30 cycles of 95°C for 30 sec, 55°C for 30 sec, 72°C for 30 sec, and (5) a final extension for 5 min at 72°C.

For quantitative real-time reverse transcription PCR (qRT-PCR) analysis of *spo0A* gene expression, *C*. *perfringens* strains were cultured overnight in FTG broth at 37°C. Next day, a 40 μl aliquot of each FTG culture was added into 1 mL of MIC containing 5% Oxyrase (Sigma-Aldrich) and those mixtures were incubated at 37°C for 3 h. RNA was extracted from those samples as described earlier. cDNA was synthesized using a Maxima first-strand cDNA synthesis kit (Thermo Fisher Scientific) from 500 ng aliquot of each purified RNA. qRT-PCR analysis was performed in triplicate using 10 ng of cDNA, Power SYBR green PCR master mix (Thermo Fisher Scientific) and a Step One Plus qRT-PCR instrument (Applied Biosystems), as previously described [44]. Primers used for qRT-PCR were listed in Table 2. The results were calculated by the comparative threshold cycle (*C*_*T*_; 2^−ΔΔCT^) method, with normalization to the *16S* transcript [44].

### *C*. *perfringens* growth curve analyses and quantitative counts of vegetative or heat-resistant spores

The growth characteristics of SM101 and its isogenic orphan histidine kinase knockout mutant or complemented strains were assessed as previously described [17]. Briefly, a 0.2 mL aliquot of an overnight FTG culture of each isolate was transferred into freshly prepared MDS medium. After incubation at 37°C for 0, 1, 3, 5, 8, or 24 h, optical density at 600 nm was measured using a Bio-Rad SmartSpec spectrophotometer.

To enumerate vegetative cells, 0.2 mL and 40 μl of overnight FTG growth of *C*. *perfringens* were inoculated into 10 mL of MDS medium or 1 mL of MIC containing 5% Oxyrase, respectively. After incubation for 18 h at 37°C, vegetative cell numbers were determined by serially diluting those cultures with sterile PBS, followed by plating aliquots of suspended cells onto BHI agar plates. Colony forming units (CFU) were enumerated after overnight anaerobic incubation at 37°C.

To compare vegetative cell numbers in MIC vs. PBS, 40 μl of overnight FTG growth of SM101 were inoculated into1 mL of either MIC or PBS, each containing 5% Oxyrase. After incubation at 37°C for 0, 4, 6, 8, or 24 h, vegetative cell numbers were determined as described above.

Sample preparation for analysis of heat-resistant spore formation in both MDS and mouse intestinal contents was similar to that used for determining vegetative cell counts. However, before plating the suspended cells onto BHI agar plates, aliquots of the cultures were heated at 70°C for 20 min to eliminate vegetative cells and enhance spore germination.

### Phase-contrast photomicroscopy

Phase-contrast photomicroscopy was performed using 12 h MDS cultures or 18 h MIC plus 5% Oxyrase cultures for wild-type SM101 and its derivative knockout mutants or complemented strains. This microscopy used a 100x phase-contrast objective on a Nikon Eclipse Ci-U microscope coupled with a mounted Nikon DS-Fi3 microscope camera to capture images of *C*. *perfringens* cells. Photographs were taken in at least three different fields of view of a culture and representative images are shown. At least two cultures for each strain were examined by microscopy.

### GusA reporter assay

pJIR750-PsigF-GusA, prepared previously [24], is a reporter plasmid where expression of the *gusA* ORF is driven from the *sigF* promoter region, i.e., this plasmid monitors expression from the *sigF* promoter region as a reporter for early gene expression during sporulation. This plasmid was electroporated into wild-type SM101, CPR1728KO, CPR1316KO, CPR1493KO, CPR1953KO or CPR1954KO. The resultant transformants were grown on BHI agar plates containing 15 μg/mL of chloramphenicol and then subjected to PCR using specific primer sets (Table 2) for confirmation of constructs. Wild-type SM101 carrying empty (no insert) pJIR750 plasmid was used as a negative control [17].

To measure GusA activity, SM101 or its isogenic null mutant transformants harboring the pJIR750-PsigF-GusA plasmid were cultured overnight in MDS medium at 37°C. Subsequently, after brief sonication, supernatant from each culture was collected and 50 μl of 6 mM 4-nitrophenyl-β-d-glucuronide (in PBS) substrate was added to 250 μl of the collected supernatant. Following a 30 min incubation at 37°C, the absorbance at 405 nm was measured. Finally, the GusA activities of each sample was calculated and the results were provided as Miller specific activity units [45].

### Statistical analysis

All values are presented as mean ± standard errors of the means from at least three independent experiments. Either Student’s unpaired *t* test or ordinary one-way analysis of variance (ANOVA; GraphPad Prism 8) was used to evaluate for statistical significance (*P* value less than 0.05) differences among the results.

## Supporting information

S1 Fig Comparison of the viable vegetative cell numbers and sporulation when SM101 is cultured in PBS or MIC with or without Oxyrase. (A) **“**Vegetative”, viable vegetative cells (CFU/mL) when SM101 was cultured overnight at 37°C in PBS with or without Oxyrase as well as MIC with or without Oxyrase. (B) “Spores”, heat-resistant spores (CFU/mL) in aliquots of those same PBS or MIC cultures. Results for panels A and B are presented as the mean ± SD of three independent experiments. Student’s unpaired *t* test was used for statistical analysis in panels A and B. Asterisk indicates *p* < 0.05.
(TIF)

S2 Fig Loading control for Figs 1–5 CPE Western blot analyses. To demonstrate that equal levels of total proteins were loaded for all samples in Western blot experiments, the same polyvinylidene difluoride (PVDF) membranes were stained, after Western blot analyses, with either Coomassie Brilliant blue G250 (for MDS samples) or Swift membrane stain kit (for MIC samples).
(TIF)

S3 Fig Construction and characterization of a *cpr1728* null mutant. (A) PCR assay confirming construction of an isogenic *cpr1728* null mutant (CPR1728KO) using the *Clostridium*-modified TargeTron knockout system. Specific internal primers for *cpr1728* amplified a larger PCR product for CPR1728KO (1175 bp) versus SM101 (275 bp), consistent with insertion of the 900 bp intron into the *cpr1728* gene of the mutant strain. (B) Southern blot hybridization of an intron-specific probe with *EcoRI*-digested DNA from SM101 or the isogenic *cpr1728*-null mutant. (C) RNA was isolated from SM101 and CPR1728KO cultured in MDS for 3 h at 37°C. The purity of each isolated RNA was demonstrated by PCR, without reverse transcriptase, for the *polC* housekeeping gene (top panel). Genomic DNA and a control sample lacking DNA template were used as positive and negative controls, respectively. (middle panel) RT-PCR analysis for the *16S* housekeeping gene as a quality control for the prepared RNA. (lower panel) RT-PCR analysis for expression of the *cpr1728* gene. (D) Growth curve analysis (measurement of culture OD_600_) for SM101 versus CPR1728KO grown in MDS medium at 37°C.
(TIF)

S4 Fig Construction and characterization of a *cpr1316* null mutant and complemented strain. (A) PCR assay confirming construction of an isogenic *cpr1316* null mutant (CPR1316KO) and complemented strain (CPR1316COM). Specific internal primers for *cpr1316* amplified a larger PCR product in CPR1316KO (1249 bp) versus wild-type SM101 (349 bp), consistent with insertion of a 900 bp intron into the *cpr1316* gene of the mutant. The complemented strain also amplified a 349 bp product using the same primers, indicating the presence of a wild-type *cpr1316* gene. (B) Southern blot hybridization of an intron-specific probe with *EcoRI*-digested DNA from SM101 or the isogenic *cpr1316*-null mutant. (C) RNA was isolated from SM101, CPR1316KO, and CPR1316COM grown in MDS for 3 h at 37°C and the purity of each isolated RNA was shown by PCR, without reverse transcriptase, for the *polC* housekeeping gene (top panel). Genomic DNA or a sample lacking DNA template were used as positive and negative controls, respectively. (middle panel) RT-PCR analysis for the *16S* housekeeping gene to demonstrate the quality of each prepared RNA. (lower panel) RT-PCR analysis for expression of the *cpr1316* gene. (D) Growth curve analysis (measurement of culture OD_600_) for SM101 versus CPR1316KO and CPR1316COM when grown in MDS medium at 37°C.
(TIF)

S5 Fig Construction and characterization of a *cpr1493* null mutant and complemented strain. (A) PCR assay confirming construction of an isogenic *cpr1493* null mutant (CPR1493KO) and complemented strain (CPR1493COM). Specific internal primers for *cpr1493* amplified a larger PCR product in CPR1493KO (1250 bp) versus wild-type SM101 (350 bp), consistent with the insertion of a 900 bp intron into the *cpr1493* gene of the mutant. The complemented strain amplified a 350 bp product when the same primers were used, indicating the presence of a wild-type *cpr1493* gene. (B) Southern blot hybridization of an intron-specific probe with *EcoRI*-digested DNA from SM101 or the isogenic *cpr1493*-null mutant. (C) RNA was isolated from SM101, CPR1493KO, and CPR1493COM grown in MDS for 3 h at 37°C and purity of each isolated RNA was demonstrated by PCR, without reverse transcriptase, for the *polC* housekeeping gene (top panel). Genomic DNA or samples lacking DNA template were used as positive and negative controls, respectively. (middle panel) RT-PCR analysis for the *16S* RNA housekeeping gene was used as a control to confirm the quality of each prepared RNA. (lower panel) RT-PCR analysis for expression of the *cpr1493* gene. (D) Growth curve analysis (measurement of culture OD_600_) for SM101 versus CPR1493KO or CPR1493COM when grown in MDS medium at 37°C.
(TIF)

S6 Fig Construction and characterization of a *cpr1953* null mutant and complemented strain. (A) PCR assay confirming construction of an isogenic *cpr1953* null mutant (CPR1953KO) and complemented strain (CPR1953COM). Specific internal primers for *cpr1953* amplified a larger PCR product using DNA from CPR1953KO (1265 bp) versus DNA from SM101 (365 bp), consistent with the insertion of a 900 bp intron into the *cpr1953* gene of the mutant. The complemented strain amplified a 365 bp product when the same primers were used, indicating the presence of a wild-type *cpr1953* gene. (B) Southern blot hybridization of an intron-specific probe with *EcoRI*-digested DNA from SM101 strain or the isogenic *cpr1953* null mutant. (C) RNA was isolated from SM101, CPR1953KO, and CPR1953COM grown in MDS for 3 h at 37°C and purity of each isolated RNA was demonstrated by PCR, without reverse transcriptase, for the *polC* housekeeping gene (top panel). Genomic DNA or a sample lacking DNA template were used as positive and negative controls, respectively. (middle panel) RT-PCR analysis for *16S* RNA expression as a housekeeping gene control for the quality of prepared RNA. (lower panel) RT-PCR analysis for expression of the *cpr1953* gene. (D) Growth curve analysis (measurement of OD_600_) for SM101 versus CPR1953KO and CPR1953COM grown in MDS at 37°C.
(TIF)

S7 Fig Construction and characterization of a *cpr1954* null mutant and complemented strain. (A) PCR assay confirming construction of an isogenic *cpr1954* null mutant (CPR1954KO) and complemented strain (CPR1954COM). Specific internal primers for the *cpr1954* gene amplified a larger PCR product in CPR1954KO (1496 bp) versus SM101 (596 bp), consistent with the insertion of a 900 bp intron into the *cpr1954* gene of the mutant. The complemented strain amplified a 596 bp product when the same primers were used, indicating the presence of a wild-type *cpr1493* gene. (B) Southern blot hybridization of an intron-specific probe with *EcoRI*-digested DNA from SM101 or the isogenic *cpr1954* null mutant. (C) RNA was isolated from SM101, CPR1954KO, and CPR1954COM grown in MDS for 3 h at 37°C and purity of each isolated RNA was demonstrated by PCR, without reverse transcriptase, using primers for the *polC* housekeeping gene (top panel). Genomic DNA or samples lacking DNA template were used as positive and negative controls, respectively. (middle panel) RT-PCR analysis of *16S* RNA housekeeping gene expression as a quality control for the prepared RNA. (lower panel) RT-PCR analysis for expression of the *cpr1954* gene. (D) Growth curve analysis (measurement of culture OD_600_) for wild-type SM101 versus CPR1954KO mutant and CPR1954COM grown in MDS medium at 37°C.
(TIF)

S8 Fig RT-PCR analysis for expression of the *cpr1953* gene in SM101 vs. CPR1953KO. (A) and (B) RNA was isolated from SM101 and CPR1953KO grown in MDS for 3 h at 37°C. RT-PCR assay was employed for expression analysis of the *cpr1953* gene. (A) The presence of a large band was observed in some cultures of some TargeTron mutants, e.g. CPR1953KO as shown in this figure. (B) However, in most cultures, no band was observed for the putative kinase mutants, e.g. CPR1953KO as shown in this figure.
(TIF)

S9 Fig Comparison of the viable vegetative cell numbers, sporulation and CPE production when SM101 or SM101-pJIR750 strains are cultured in MDS or MIC. (A) **“**Vegetative”, viable vegetative cells (CFU/mL) when SM101 or SM101(pJIR750) were cultured overnight at 37°C in MDS. “Spores”, heat-resistant spores (CFU/mL) in aliquots of those same MDS cultures. (B) SM101 or SM101(pJIR750) were cultured overnight at 37°C in MDS and supernatant of each culture was then subjected to Western blot analysis for CPE toxin production. (C) “Vegetative”, viable vegetative cells (CFU/mL) for SM101 or SM101(pJIR750) cultured overnight at 37°C in MIC. “Spores”, heat-resistant spores (CFU/mL) for those same MIC cultures. (D) SM101 or SM101(pJIR750) were cultured overnight at 37°C in MIC and supernatant of each culture was then subjected to Western blot analysis for CPE toxin production. Results for panels A and C are presented as the mean ± SD of three independent experiments. Student’s unpaired *t* test was used for statistical analysis in panels A and C. *p* values were < 0.05 for all pairwise comparisons. A loading control for this Western blot is presented in S10 Fig.
(TIF)

S10 Fig Loading control for CPE Western blot analyses shown in Figs 6B, 6B, 7B, 7B, 12A, 12A, S9B and S9D. To demonstrate that equal levels of total proteins were loaded for all samples in Western blot experiments, the same polyvinylidene difluoride (PVDF) membranes were stained, after Western blot analyses, with either Coomassie Brilliant blue G250 (for MDS samples) or Swift membrane stain kit (for MIC samples).
(TIF)

S11 Fig Loading control for CPE Western blot analyses shown in Figs 13 or 15. To demonstrate that equal levels of total proteins were loaded for all samples in Western blot experiments, the same polyvinylidene difluoride (PVDF) membranes were stained, after Western blot analyses, with either Coomassie Brilliant blue G250 (for MDS samples) or Swift membrane stain kit (for MIC samples).
(TIF)

S12 Fig Construction and characterization of CPR1316COM-H538A complementing strain. (A) PCR assay confirming construction of CPR1316COM-H538A complementing strain. Specific internal primers for *cpr1316* amplified a larger PCR product in CPR1316KO (1249 bp) versus wild-type SM101 (349 bp), consistent with insertion of a 900 bp intron into the *cpr1316* gene of the mutant. The complemented strain also amplified a 349 bp product using the same primers, consistent with the introduction of the complementing *cpr1316* gene encoding an alanine substitution for the key functional His residue into CPR1316KO. (B) RNA was isolated from SM101, CPR1316KO, and CPR1316COM-H538A grown in MDS for 3 h at 37°C and the purity of each isolated RNA was shown by PCR, without reverse transcriptase, for the *polC* housekeeping gene (top panel). Genomic DNA or a sample lacking DNA template were used as positive and negative controls, respectively. (middle panel) RT-PCR analysis for the *16S* housekeeping gene to demonstrate the quality of each prepared RNA. (lower panel) RT-PCR analysis for expression of the *cpr1316* gene.
(TIF)

S13 Fig Construction and characterization of CPR1493COM-H863A complementing strain. (A) PCR assay confirming construction of CPR1493COM-H863A complementing strain. Specific internal primers for *cpr1493* amplified a larger PCR product in CPR1493KO (1250 bp) versus wild-type SM101 (350 bp), consistent with the insertion of a 900 bp intron into the *cpr1493* gene of the mutant. The complemented strain amplified a 350 bp product when the same primers were used, consistent with the introduction of the complementing *cpr1493* gene encoding an alanine substitution for the key functional His residue into CPR1493KO. (B) RNA was isolated from SM101, CPR1493KO, and CPR1493COM-H863A grown in MDS for 3 h at 37°C and the purity of each isolated RNA was shown by PCR, without reverse transcriptase, for the *polC* housekeeping gene (top panel). Genomic DNA or a sample lacking DNA template were used as positive and negative controls, respectively. (middle panel) RT-PCR analysis for the *16S* housekeeping gene to demonstrate the quality of each prepared RNA. (lower panel) RT-PCR analysis for expression of the *cpr1493* gene.
(TIF)

S14 Fig Construction and characterization of CPR1953COM-H430A complementing strain. (A) PCR assay confirming construction of CPR1953COM-H430A complementing strain. Specific internal primers for *cpr1953* amplified a larger PCR product using DNA from CPR1953KO (1265 bp) versus DNA from SM101 (365 bp), consistent with the insertion of a 900 bp intron into the *cpr1953* gene of the mutant. The complemented strain amplified a 365 bp product when the same primers were used, consistent with the introduction of the complementing *cpr1953* gene encoding an alanine substitution for the key functional His residue into CPR1953KO. (B) RNA was isolated from SM101, CPR1953KO, and CPR1953COM-H430A grown in MDS for 3 h at 37°C and the purity of each isolated RNA was shown by PCR, without reverse transcriptase, for the *polC* housekeeping gene (top panel). Genomic DNA or a sample lacking DNA template were used as positive and negative controls, respectively. (middle panel) RT-PCR analysis for the *16S* housekeeping gene to demonstrate the quality of each prepared RNA. (lower panel) RT-PCR analysis for expression of the *cpr1953* gene.
(TIF)

S15 Fig Construction and characterization of CPR1954COM-H410A complementing strain. (A) PCR assay confirming construction of CPR1954COM-H410A complementing strain. Specific internal primers for the *cpr1954* gene amplified a larger PCR product in CPR1954KO (1496 bp) versus SM101 (596 bp), consistent with the insertion of a 900 bp intron into the *cpr1954* gene of the mutant. The complemented strain amplified a 596 bp product when the same primers were used, consistent with the introduction of the complementing *cpr1954* gene encoding an alanine substitution for the key functional His residue into CPR1954KO. (B) RNA was isolated from SM101, CPR1954KO, and CPR1954COM-H410A grown in MDS for 3 h at 37°C and the purity of each isolated RNA was shown by PCR, without reverse transcriptase, for the *polC* housekeeping gene (top panel). Genomic DNA or a sample lacking DNA template were used as positive and negative controls, respectively. (middle panel) RT-PCR analysis for the *16S* housekeeping gene to demonstrate the quality of each prepared RNA. (lower panel) RT-PCR analysis for expression of the *cpr1954* gene.
(TIF)
