# Supplementary figures and images for: Identification of orphan histidine kinases that impact sporulation and enterotoxin production by Clostridium perfringens type F strain SM101 in a pathophysiologically-relevant ex vivo mouse intestinal contents model
Source: PLoS Pathog. 2023 Jun 1;19(6):e1011429. doi: 10.1371/journal.ppat.1011429 (PMC10263361; doi:10.1371/journal.ppat.1011429)

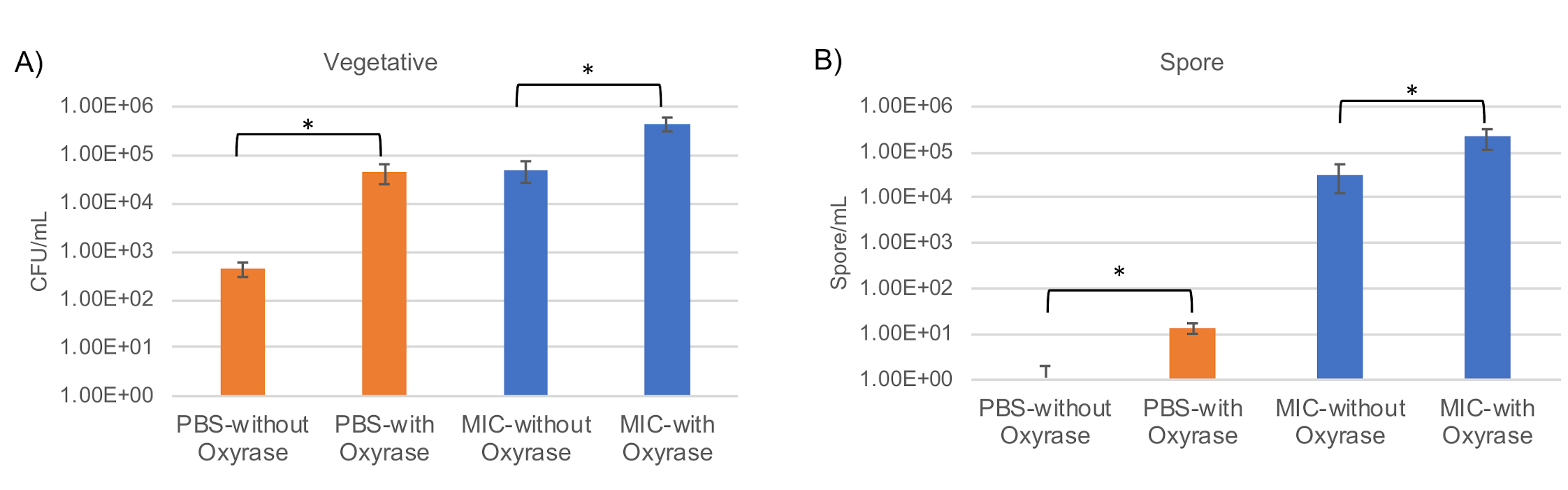

Supplement: S1 Fig — (A) “Vegetative”, viable vegetative cells (CFU/mL) when SM101 was cultured overnight at 37°C in PBS with or without Oxyrase as well as MIC with or without Oxyrase. (B) “Spores”, heat-resistant spores (CFU/mL) in aliquots of those same PBS or MIC cultures. Results for panels A and B are presented as the mean ± SD of three independent experiments. Student’s unpaired t test was used for statistical analysis in panels A and B. Asterisk indicates p < 0.05. (TIF) [file ppat.1011429.s001.tif]

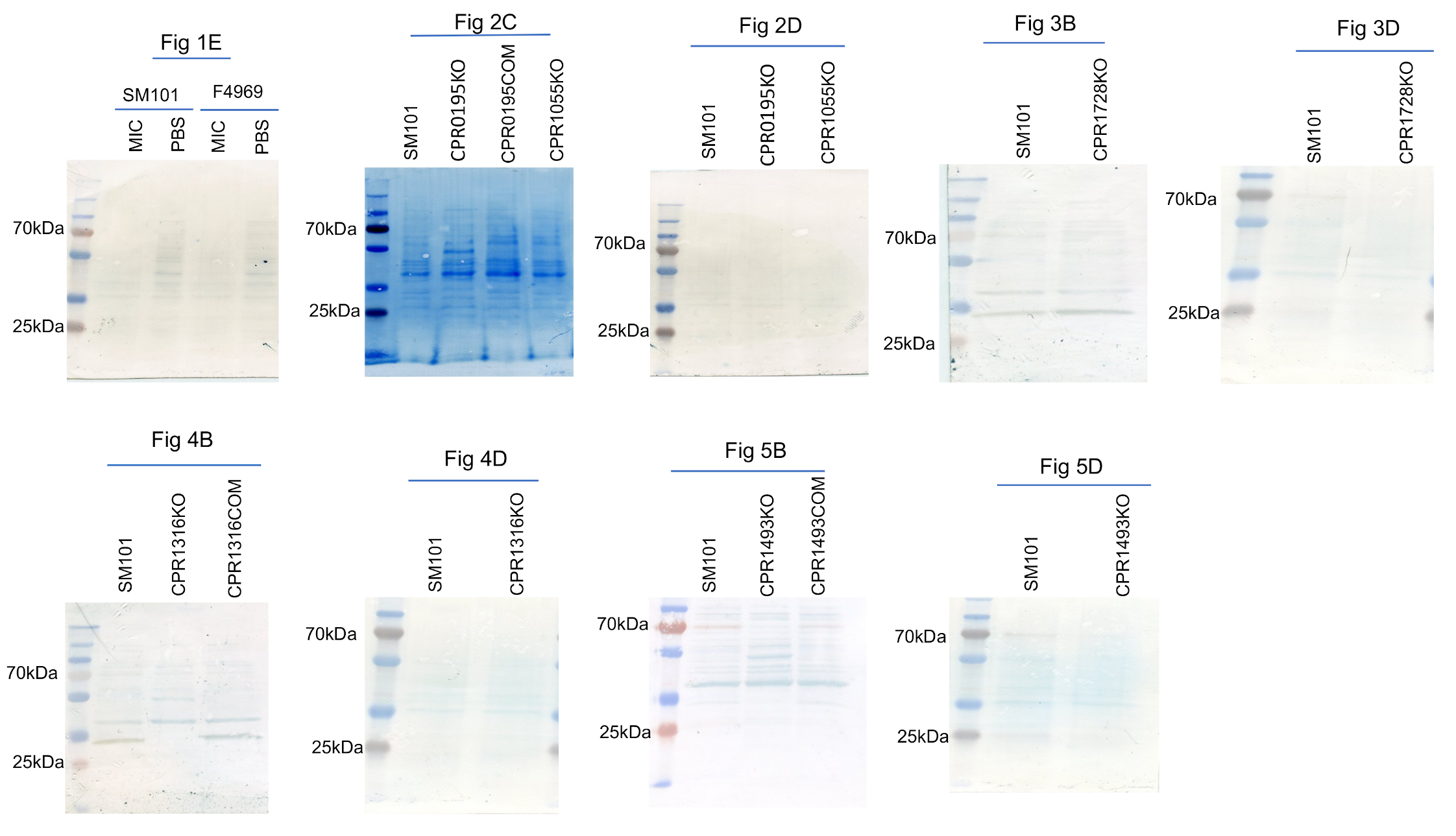

Supplement: S2 Fig — To demonstrate that equal levels of total proteins were loaded for all samples in Western blot experiments, the same polyvinylidene difluoride (PVDF) membranes were stained, after Western blot analyses, with either Coomassie Brilliant blue G250 (for MDS samples) or Swift membrane stain kit (for MIC samples). (TIF) [file ppat.1011429.s002.tif]

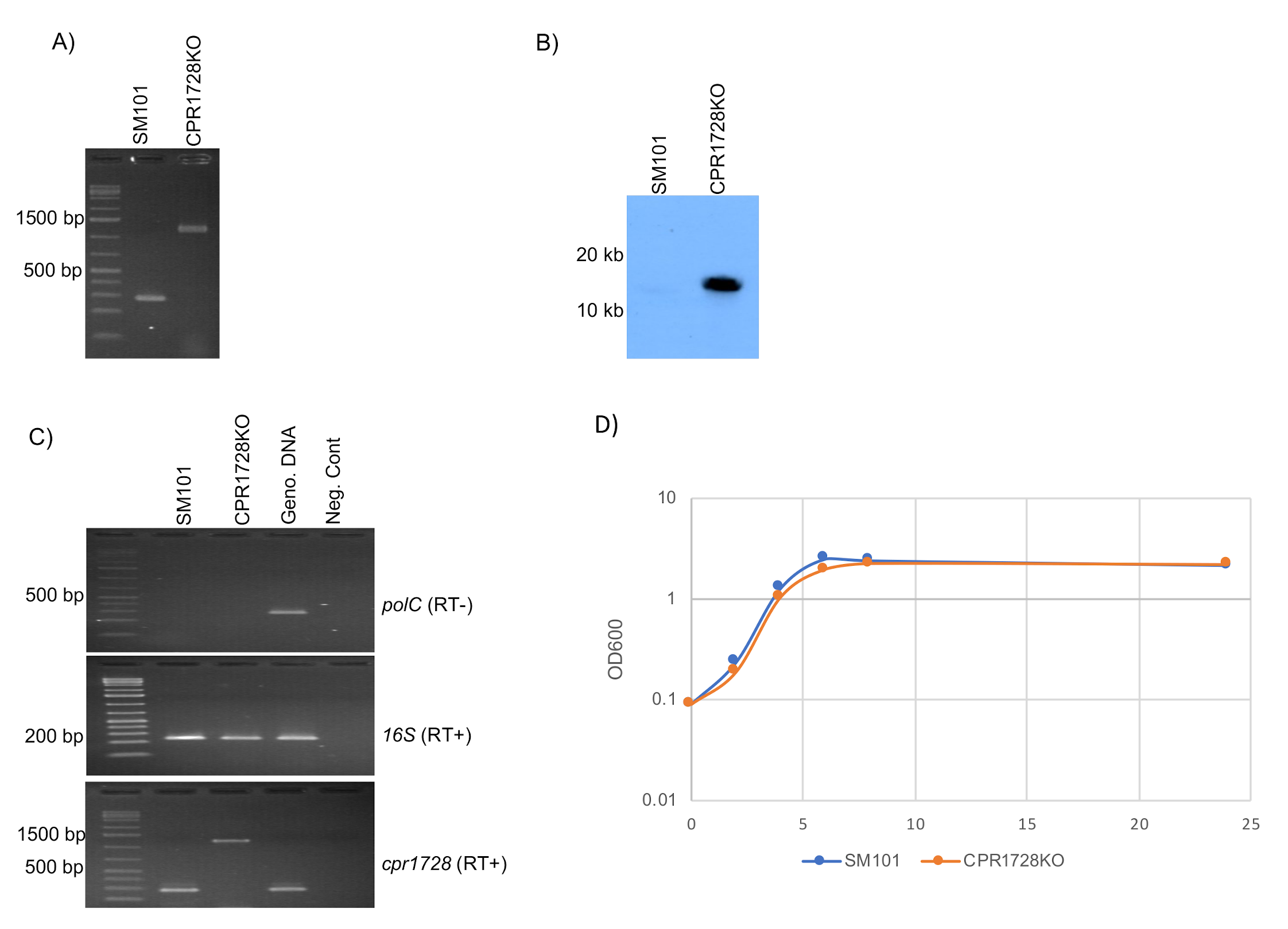

Supplement: S3 Fig — (A) PCR assay confirming construction of an isogenic cpr1728 null mutant (CPR1728KO) using the Clostridium-modified TargeTron knockout system. Specific internal primers for cpr1728 amplified a larger PCR product for CPR1728KO (1175 bp) versus SM101 (275 bp), consistent with insertion of the 900 bp intron into the cpr1728 gene of the mutant strain. (B) Southern blot hybridization of an intron-specific probe with EcoRI-digested DNA from SM101 or the isogenic cpr1728-null mutant. (C) RNA was isolated from SM101 and CPR1728KO cultured in MDS for 3 h at 37°C. The purity of each isolated RNA was demonstrated by PCR, without reverse transcriptase, for the polC housekeeping gene (top panel). Genomic DNA and a control sample lacking DNA template were used as positive and negative controls, respectively. (middle panel) RT-PCR analysis for the 16S housekeeping gene as a quality control for the prepared RNA. (lower panel) RT-PCR analysis for expression of the cpr1728 gene. (D) Growth curve analysis (measurement of culture OD600) for SM101 versus CPR1728KO grown in MDS medium at 37°C. (TIF) [file ppat.1011429.s003.tif]

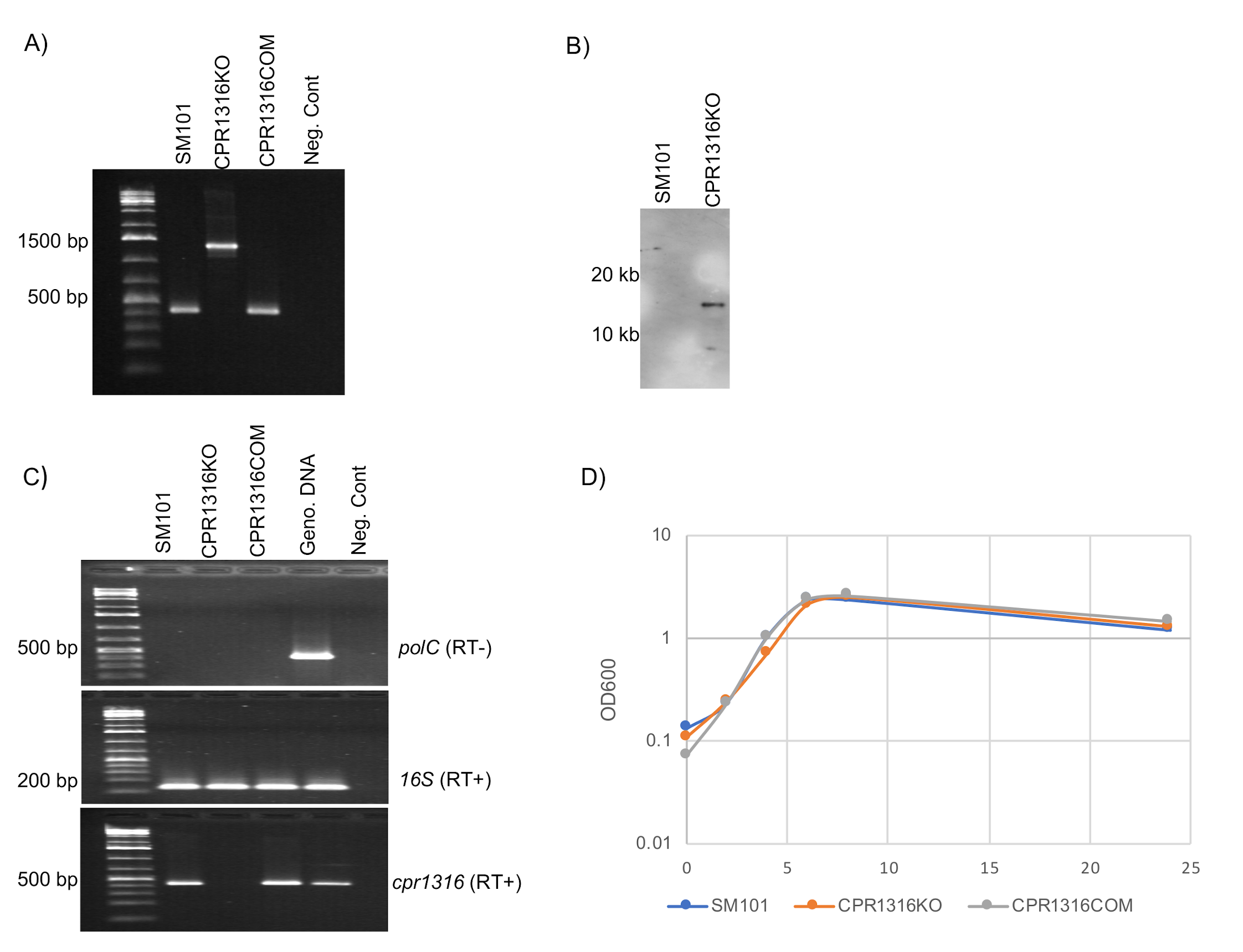

Supplement: S4 Fig — (A) PCR assay confirming construction of an isogenic cpr1316 null mutant (CPR1316KO) and complemented strain (CPR1316COM). Specific internal primers for cpr1316 amplified a larger PCR product in CPR1316KO (1249 bp) versus wild-type SM101 (349 bp), consistent with insertion of a 900 bp intron into the cpr1316 gene of the mutant. The complemented strain also amplified a 349 bp product using the same primers, indicating the presence of a wild-type cpr1316 gene. (B) Southern blot hybridization of an intron-specific probe with EcoRI-digested DNA from SM101 or the isogenic cpr1316-null mutant. (C) RNA was isolated from SM101, CPR1316KO, and CPR1316COM grown in MDS for 3 h at 37°C and the purity of each isolated RNA was shown by PCR, without reverse transcriptase, for the polC housekeeping gene (top panel). Genomic DNA or a sample lacking DNA template were used as positive and negative controls, respectively. (middle panel) RT-PCR analysis for the 16S housekeeping gene to demonstrate the quality of each prepared RNA. (lower panel) RT-PCR analysis for expression of the cpr1316 gene. (D) Growth curve analysis (measurement of culture OD600) for SM101 versus CPR1316KO and CPR1316COM when grown in MDS medium at 37°C. (TIF) [file ppat.1011429.s004.tif]

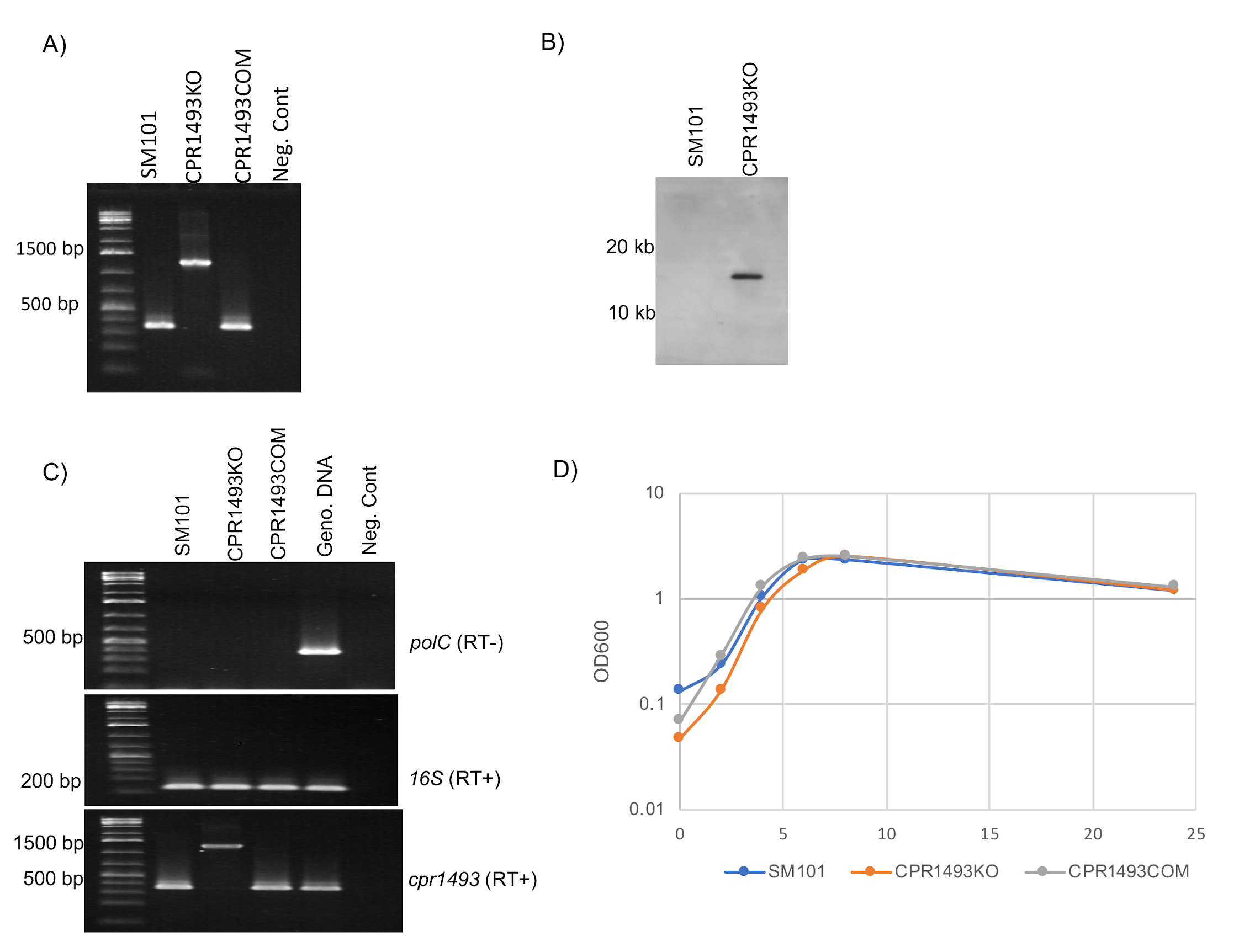

Supplement: S5 Fig — (A) PCR assay confirming construction of an isogenic cpr1493 null mutant (CPR1493KO) and complemented strain (CPR1493COM). Specific internal primers for cpr1493 amplified a larger PCR product in CPR1493KO (1250 bp) versus wild-type SM101 (350 bp), consistent with the insertion of a 900 bp intron into the cpr1493 gene of the mutant. The complemented strain amplified a 350 bp product when the same primers were used, indicating the presence of a wild-type cpr1493 gene. (B) Southern blot hybridization of an intron-specific probe with EcoRI-digested DNA from SM101 or the isogenic cpr1493-null mutant. (C) RNA was isolated from SM101, CPR1493KO, and CPR1493COM grown in MDS for 3 h at 37°C and purity of each isolated RNA was demonstrated by PCR, without reverse transcriptase, for the polC housekeeping gene (top panel). Genomic DNA or samples lacking DNA template were used as positive and negative controls, respectively. (middle panel) RT-PCR analysis for the 16S RNA housekeeping gene was used as a control to confirm the quality of each prepared RNA. (lower panel) RT-PCR analysis for expression of the cpr1493 gene. (D) Growth curve analysis (measurement of culture OD600) for SM101 versus CPR1493KO or CPR1493COM when grown in MDS medium at 37°C. (TIF) [file ppat.1011429.s005.tif]

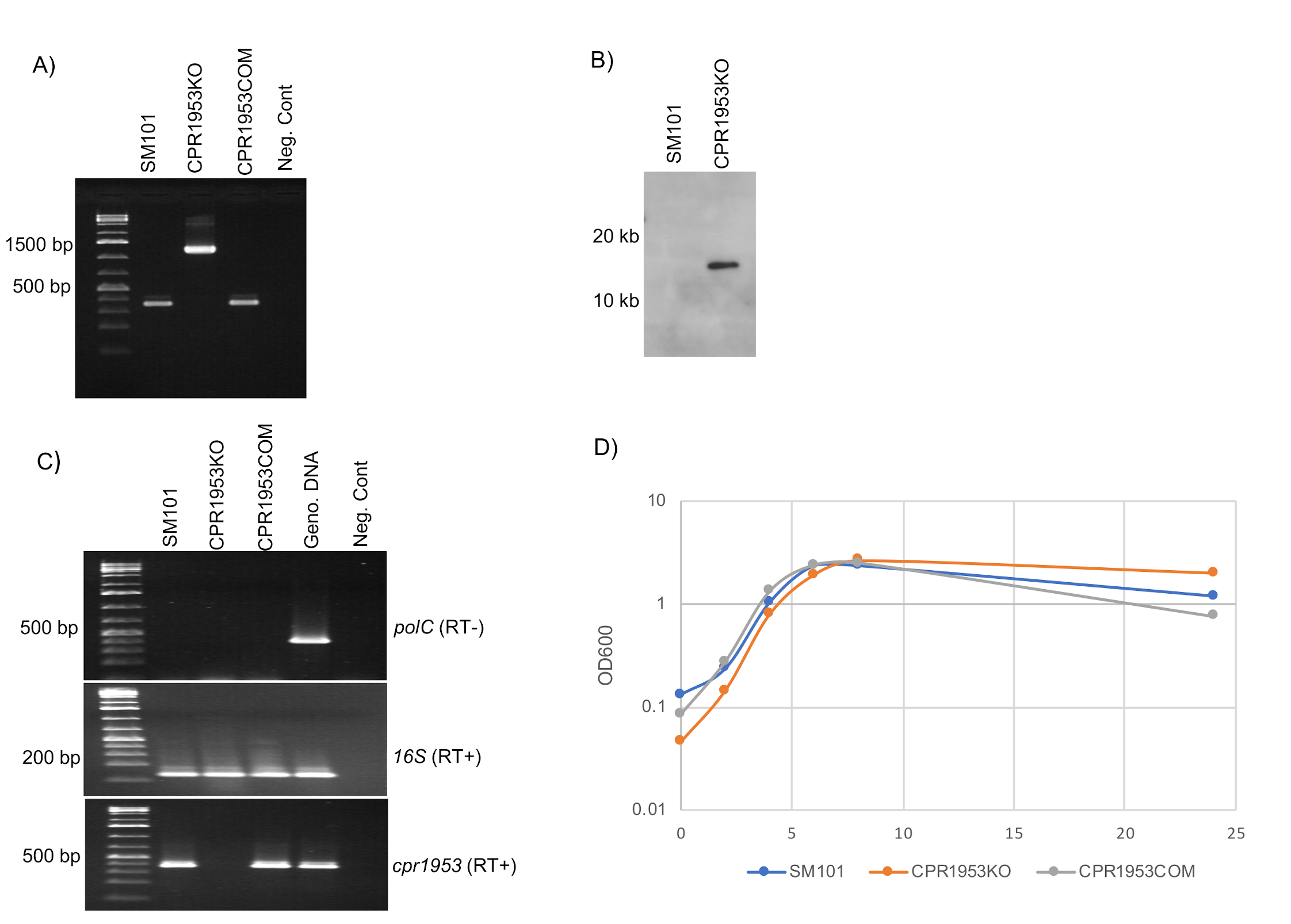

Supplement: S6 Fig — (A) PCR assay confirming construction of an isogenic cpr1953 null mutant (CPR1953KO) and complemented strain (CPR1953COM). Specific internal primers for cpr1953 amplified a larger PCR product using DNA from CPR1953KO (1265 bp) versus DNA from SM101 (365 bp), consistent with the insertion of a 900 bp intron into the cpr1953 gene of the mutant. The complemented strain amplified a 365 bp product when the same primers were used, indicating the presence of a wild-type cpr1953 gene. (B) Southern blot hybridization of an intron-specific probe with EcoRI-digested DNA from SM101 strain or the isogenic cpr1953 null mutant. (C) RNA was isolated from SM101, CPR1953KO, and CPR1953COM grown in MDS for 3 h at 37°C and purity of each isolated RNA was demonstrated by PCR, without reverse transcriptase, for the polC housekeeping gene (top panel). Genomic DNA or a sample lacking DNA template were used as positive and negative controls, respectively. (middle panel) RT-PCR analysis for 16S RNA expression as a housekeeping gene control for the quality of prepared RNA. (lower panel) RT-PCR analysis for expression of the cpr1953 gene. (D) Growth curve analysis (measurement of OD600) for SM101 versus CPR1953KO and CPR1953COM grown in MDS at 37°C. (TIF) [file ppat.1011429.s006.tif]

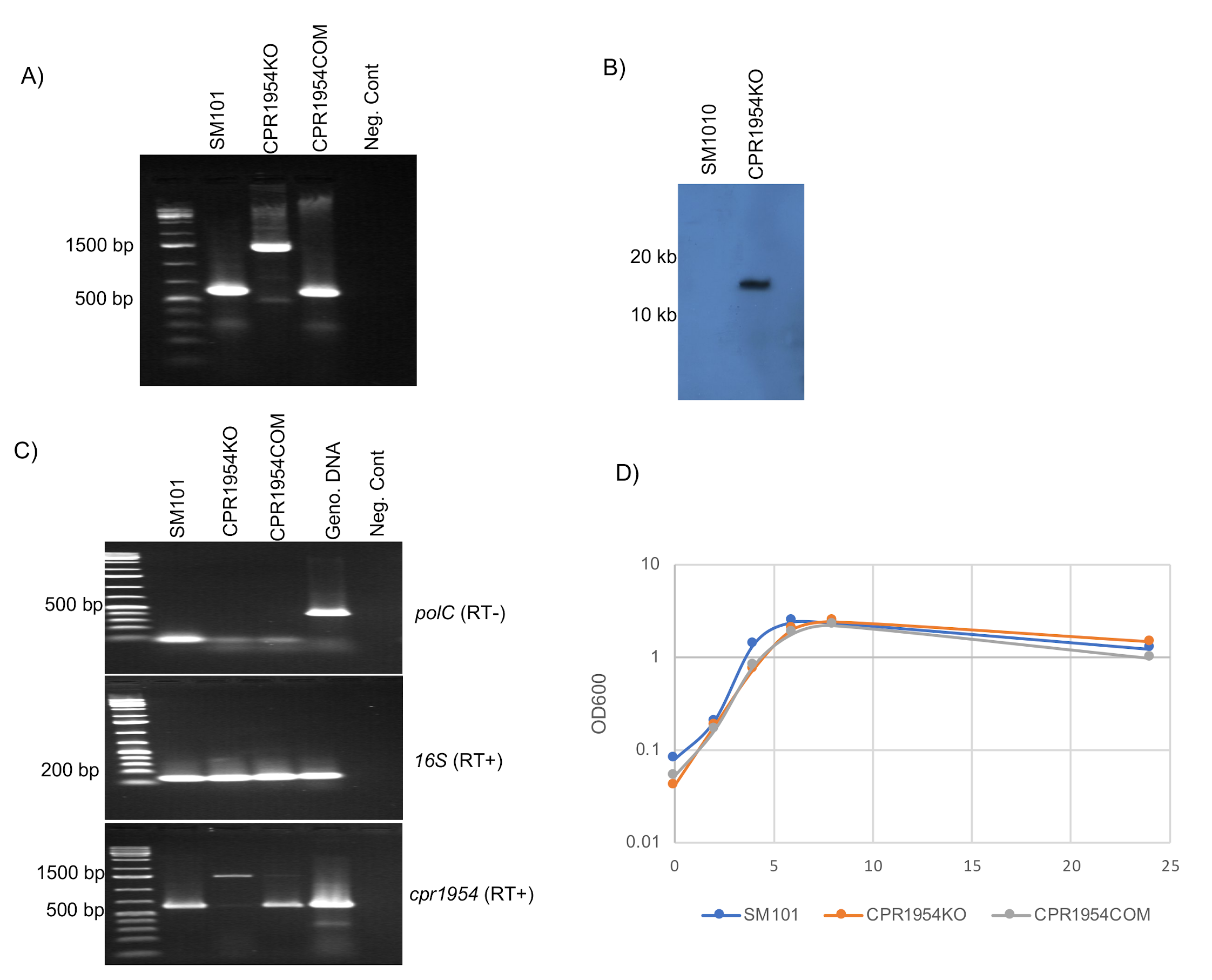

Supplement: S7 Fig — (A) PCR assay confirming construction of an isogenic cpr1954 null mutant (CPR1954KO) and complemented strain (CPR1954COM). Specific internal primers for the cpr1954 gene amplified a larger PCR product in CPR1954KO (1496 bp) versus SM101 (596 bp), consistent with the insertion of a 900 bp intron into the cpr1954 gene of the mutant. The complemented strain amplified a 596 bp product when the same primers were used, indicating the presence of a wild-type cpr1493 gene. (B) Southern blot hybridization of an intron-specific probe with EcoRI-digested DNA from SM101 or the isogenic cpr1954 null mutant. (C) RNA was isolated from SM101, CPR1954KO, and CPR1954COM grown in MDS for 3 h at 37°C and purity of each isolated RNA was demonstrated by PCR, without reverse transcriptase, using primers for the polC housekeeping gene (top panel). Genomic DNA or samples lacking DNA template were used as positive and negative controls, respectively. (middle panel) RT-PCR analysis of 16S RNA housekeeping gene expression as a quality control for the prepared RNA. (lower panel) RT-PCR analysis for expression of the cpr1954 gene. (D) Growth curve analysis (measurement of culture OD600) for wild-type SM101 versus CPR1954KO mutant and CPR1954COM grown in MDS medium at 37°C. (TIF) [file ppat.1011429.s007.tif]

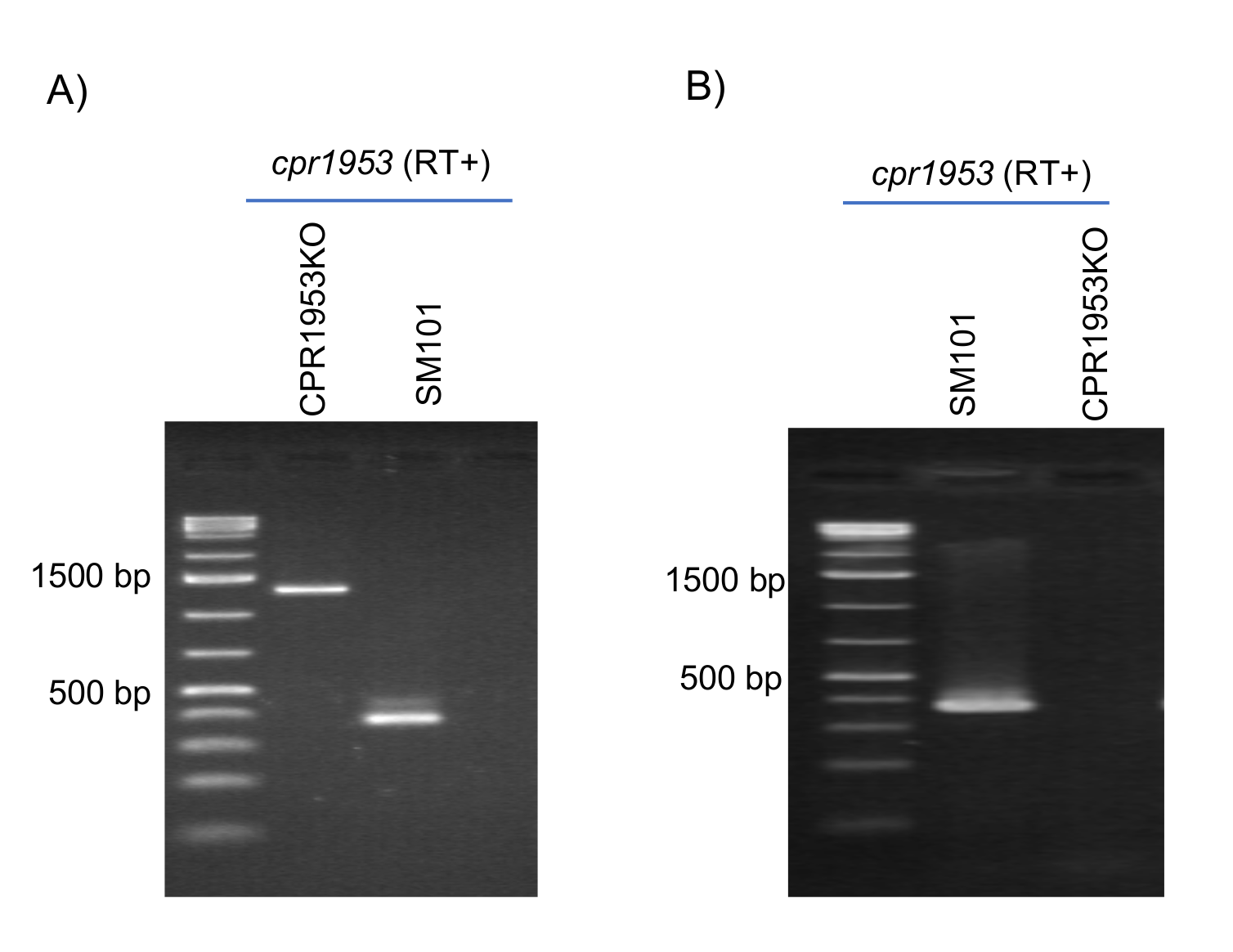

Supplement: S8 Fig — (A) and (B) RNA was isolated from SM101 and CPR1953KO grown in MDS for 3 h at 37°C. RT-PCR assay was employed for expression analysis of the cpr1953 gene. (A) The presence of a large band was observed in some cultures of some TargeTron mutants, e.g. CPR1953KO as shown in this figure. (B) However, in most cultures, no band was observed for the putative kinase mutants, e.g. CPR1953KO as shown in this figure. (TIF) [file ppat.1011429.s008.tif]

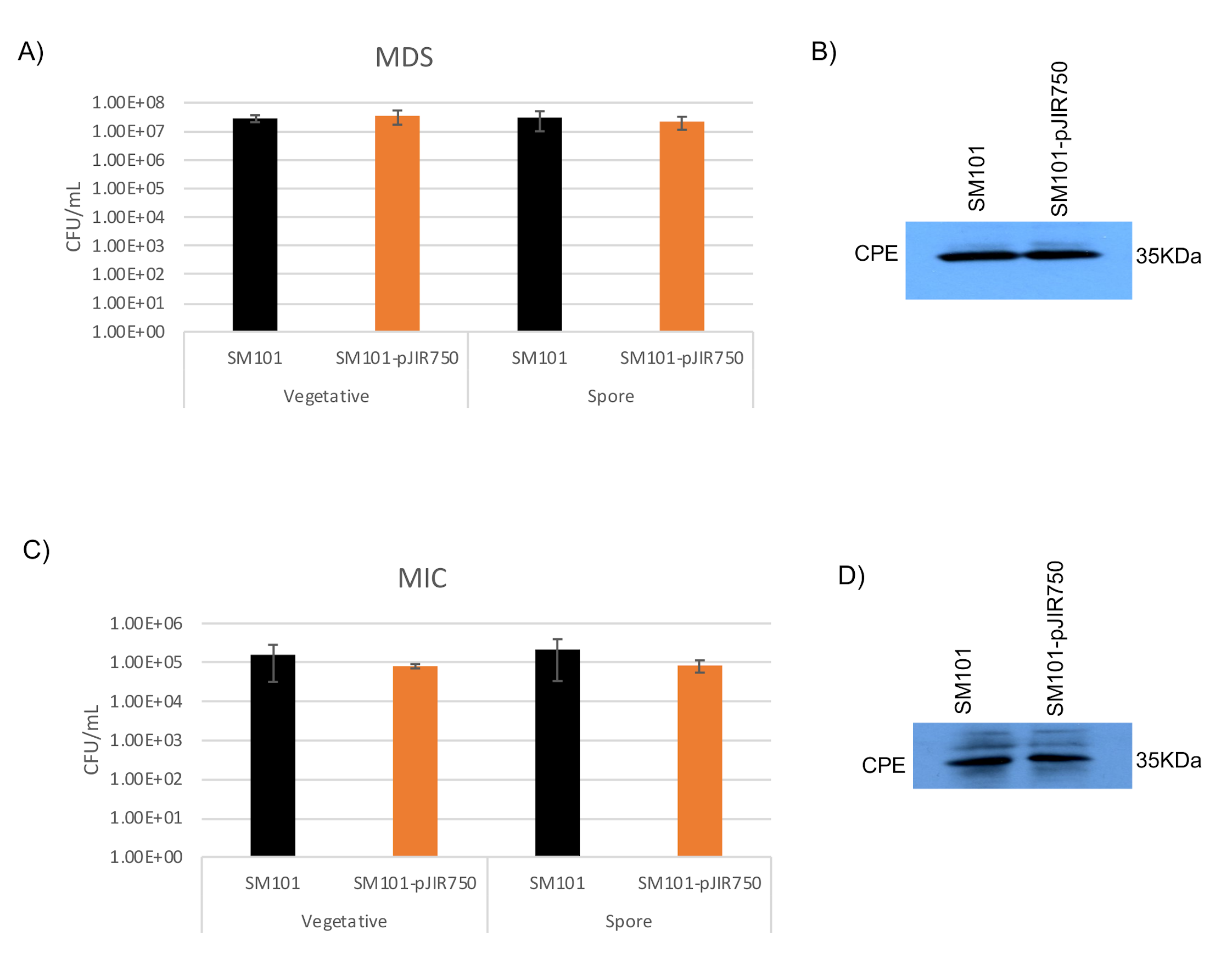

Supplement: S9 Fig — (A) “Vegetative”, viable vegetative cells (CFU/mL) when SM101 or SM101(pJIR750) were cultured overnight at 37°C in MDS. “Spores”, heat-resistant spores (CFU/mL) in aliquots of those same MDS cultures. (B) SM101 or SM101(pJIR750) were cultured overnight at 37°C in MDS and supernatant of each culture was then subjected to Western blot analysis for CPE toxin production. (C) “Vegetative”, viable vegetative cells (CFU/mL) for SM101 or SM101(pJIR750) cultured overnight at 37°C in MIC. “Spores”, heat-resistant spores (CFU/mL) for those same MIC cultures. (D) SM101 or SM101(pJIR750) were cultured overnight at 37°C in MIC and supernatant of each culture was then subjected to Western blot analysis for CPE toxin production. Results for panels A and C are presented as the mean ± SD of three independent experiments. Student’s unpaired t test was used for statistical analysis in panels A and C. p values were < 0.05 for all pairwise comparisons. A loading control for this Western blot is presented in S10 Fig. (TIF) [file ppat.1011429.s009.tif]

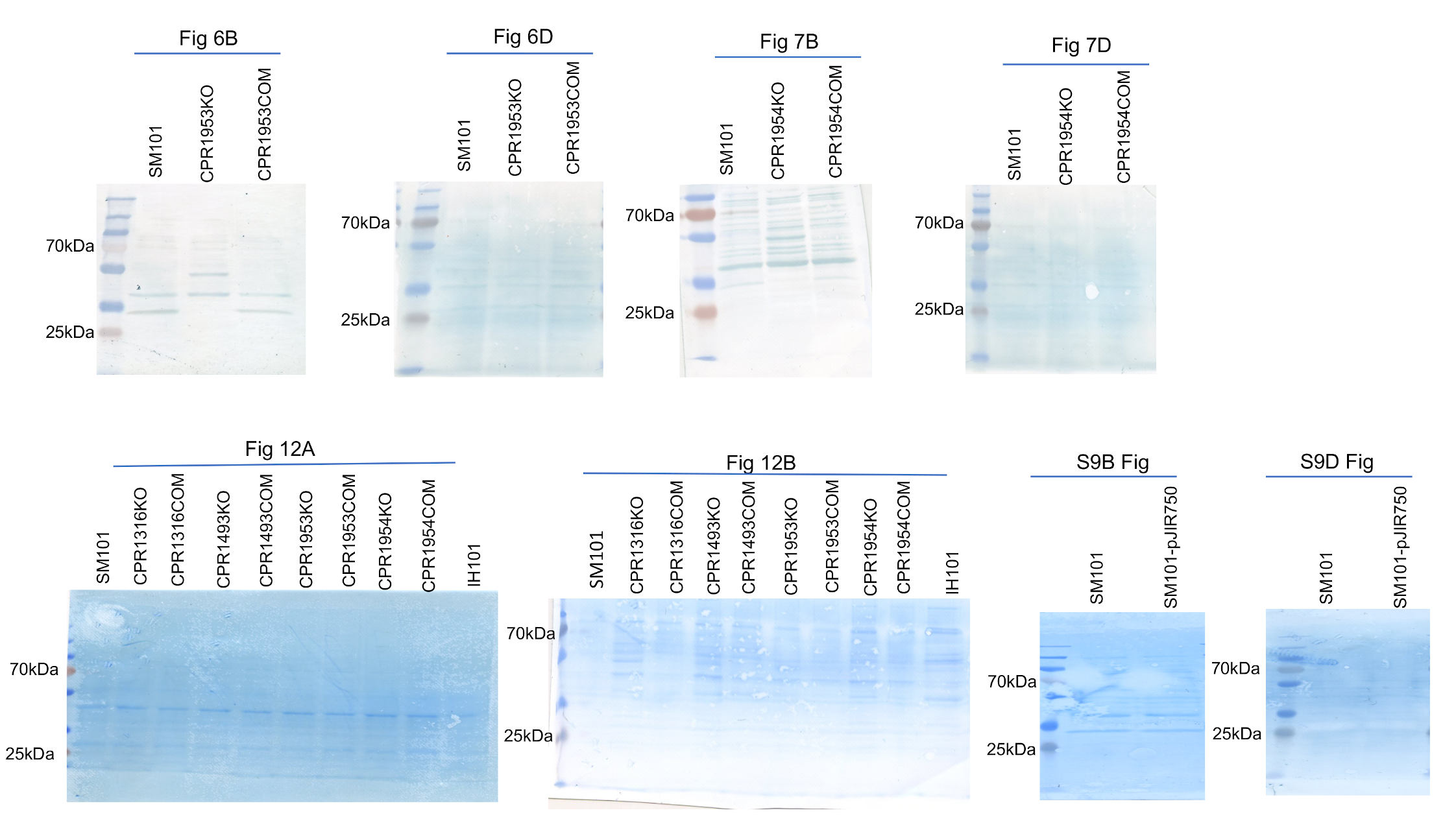

Supplement: S10 Fig — To demonstrate that equal levels of total proteins were loaded for all samples in Western blot experiments, the same polyvinylidene difluoride (PVDF) membranes were stained, after Western blot analyses, with either Coomassie Brilliant blue G250 (for MDS samples) or Swift membrane stain kit (for MIC samples). (TIF) [file ppat.1011429.s010.tif]

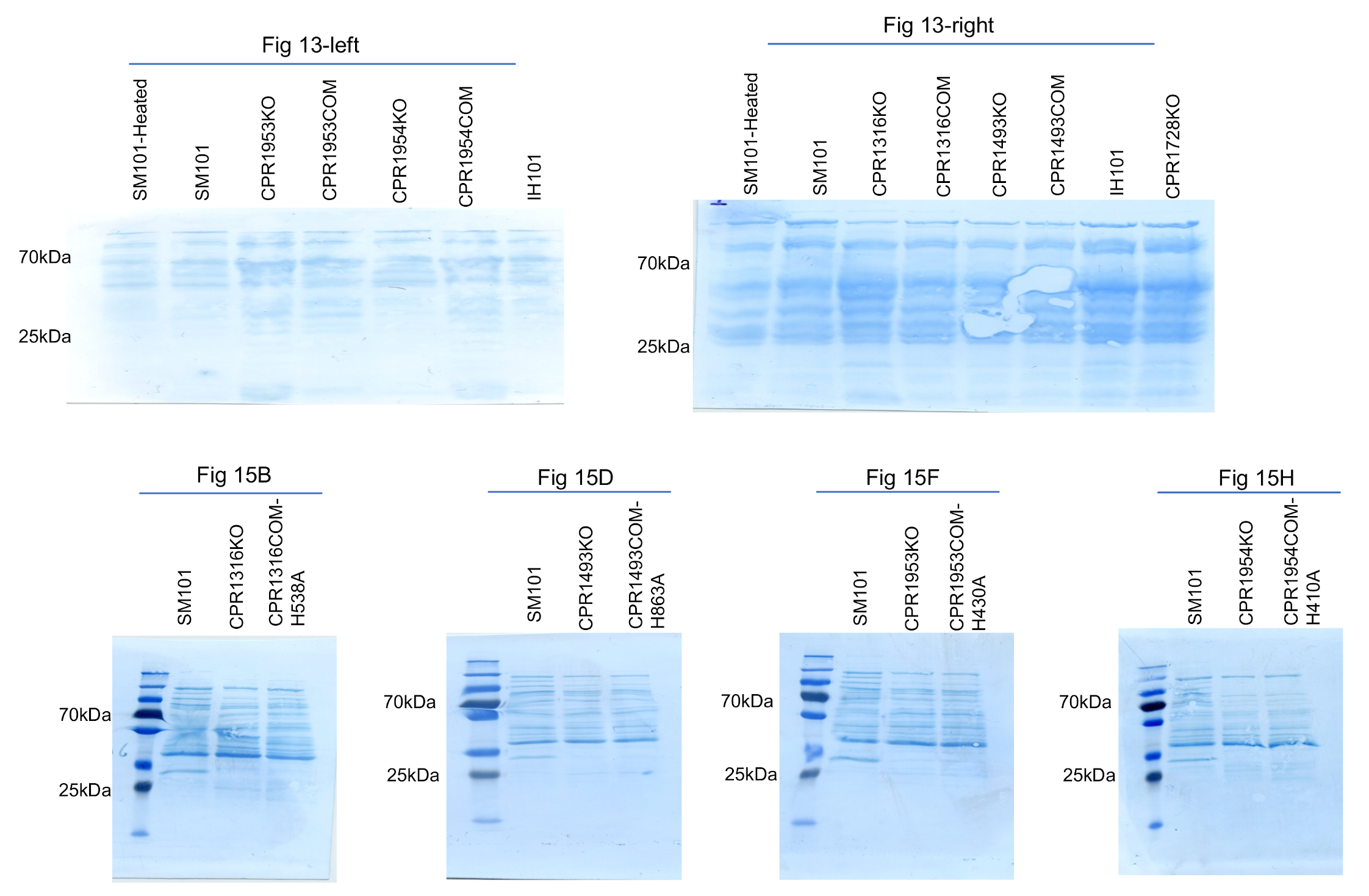

Supplement: S11 Fig — To demonstrate that equal levels of total proteins were loaded for all samples in Western blot experiments, the same polyvinylidene difluoride (PVDF) membranes were stained, after Western blot analyses, with either Coomassie Brilliant blue G250 (for MDS samples) or Swift membrane stain kit (for MIC samples). (TIF) [file ppat.1011429.s011.tif]

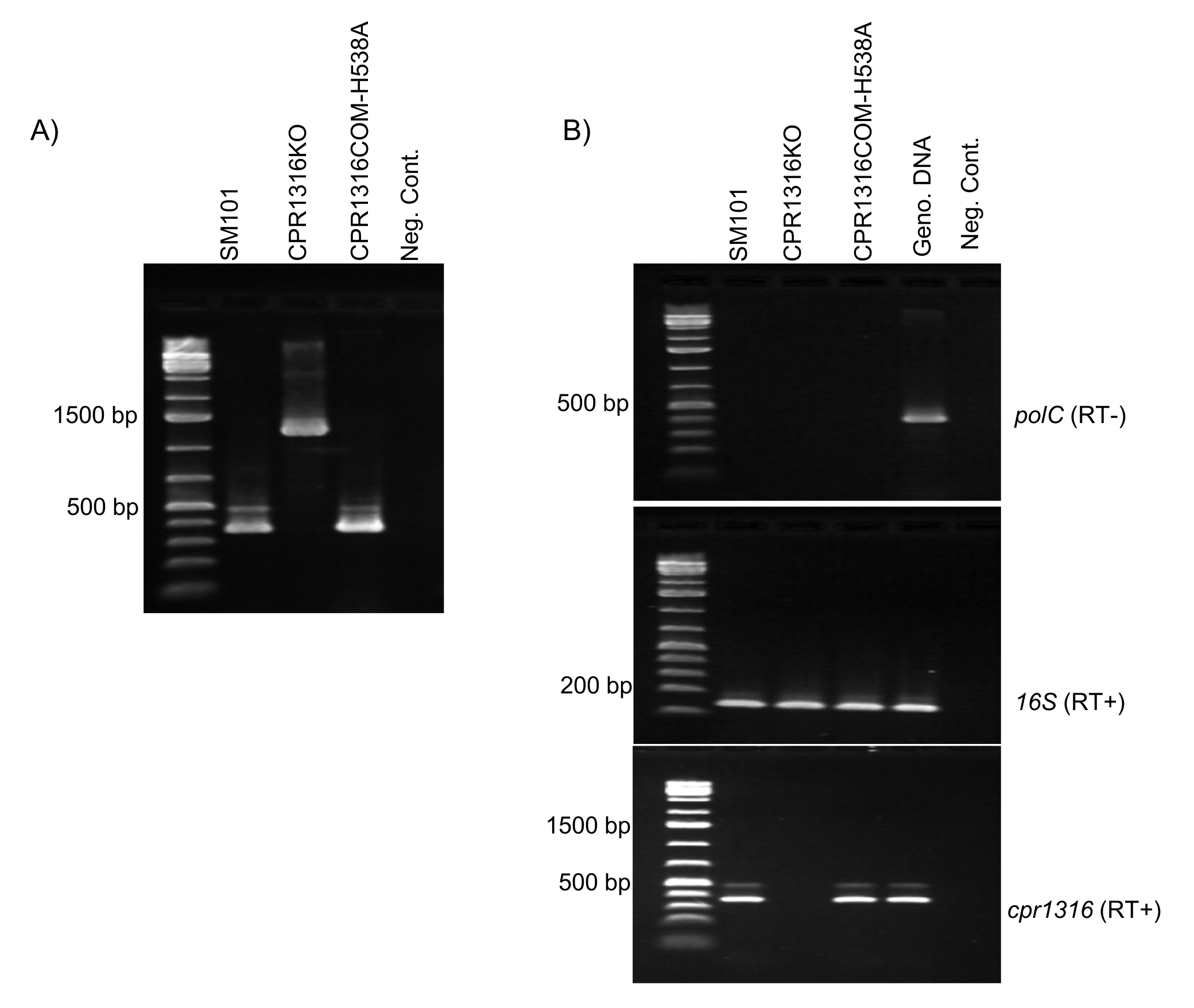

Supplement: S12 Fig — (A) PCR assay confirming construction of CPR1316COM-H538A complementing strain. Specific internal primers for cpr1316 amplified a larger PCR product in CPR1316KO (1249 bp) versus wild-type SM101 (349 bp), consistent with insertion of a 900 bp intron into the cpr1316 gene of the mutant. The complemented strain also amplified a 349 bp product using the same primers, consistent with the introduction of the complementing cpr1316 gene encoding an alanine substitution for the key functional His residue into CPR1316KO. (B) RNA was isolated from SM101, CPR1316KO, and CPR1316COM-H538A grown in MDS for 3 h at 37°C and the purity of each isolated RNA was shown by PCR, without reverse transcriptase, for the polC housekeeping gene (top panel). Genomic DNA or a sample lacking DNA template were used as positive and negative controls, respectively. (middle panel) RT-PCR analysis for the 16S housekeeping gene to demonstrate the quality of each prepared RNA. (lower panel) RT-PCR analysis for expression of the cpr1316 gene. (TIF) [file ppat.1011429.s012.tif]

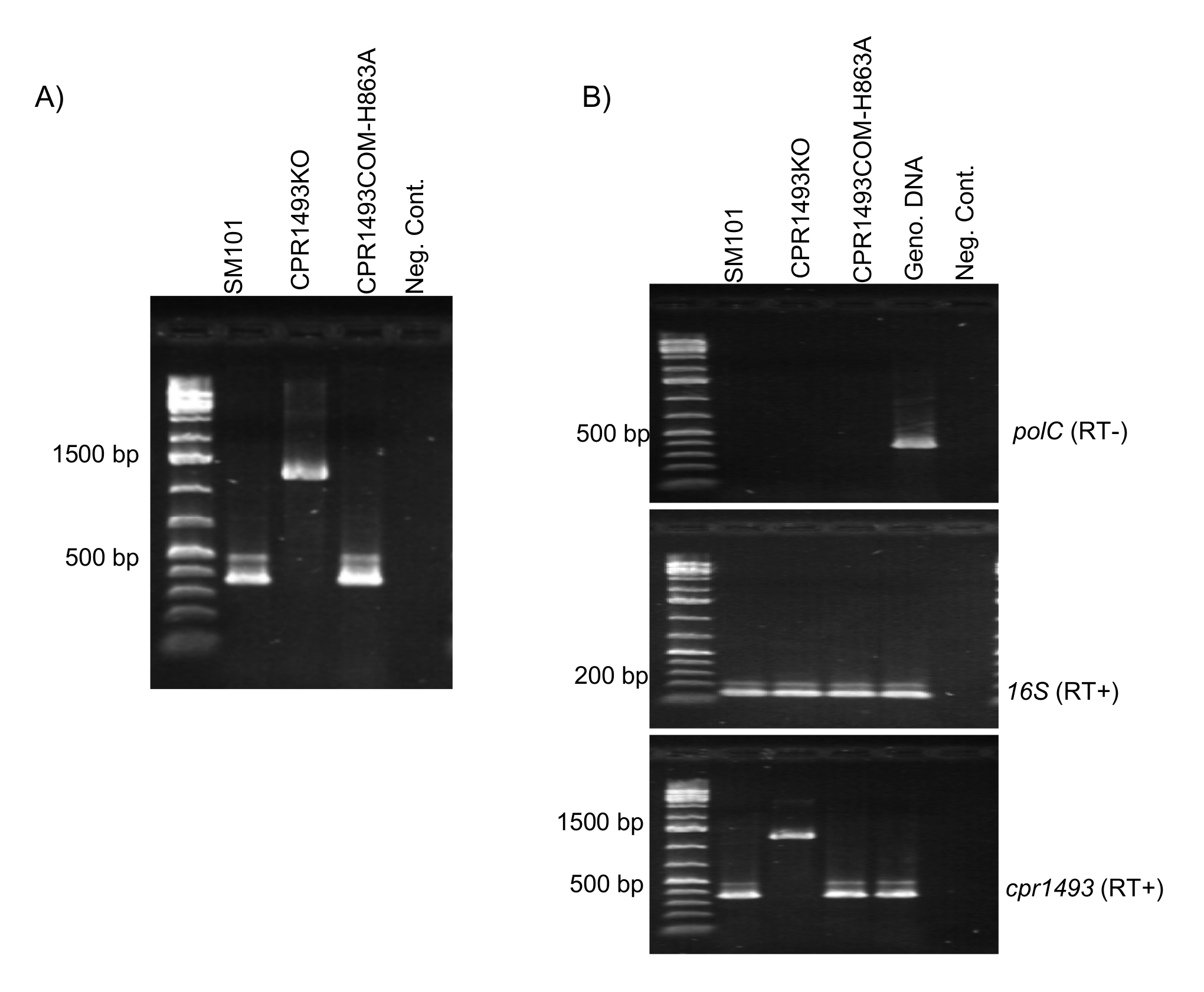

Supplement: S13 Fig — (A) PCR assay confirming construction of CPR1493COM-H863A complementing strain. Specific internal primers for cpr1493 amplified a larger PCR product in CPR1493KO (1250 bp) versus wild-type SM101 (350 bp), consistent with the insertion of a 900 bp intron into the cpr1493 gene of the mutant. The complemented strain amplified a 350 bp product when the same primers were used, consistent with the introduction of the complementing cpr1493 gene encoding an alanine substitution for the key functional His residue into CPR1493KO. (B) RNA was isolated from SM101, CPR1493KO, and CPR1493COM-H863A grown in MDS for 3 h at 37°C and the purity of each isolated RNA was shown by PCR, without reverse transcriptase, for the polC housekeeping gene (top panel). Genomic DNA or a sample lacking DNA template were used as positive and negative controls, respectively. (middle panel) RT-PCR analysis for the 16S housekeeping gene to demonstrate the quality of each prepared RNA. (lower panel) RT-PCR analysis for expression of the cpr1493 gene. (TIF) [file ppat.1011429.s013.tif]

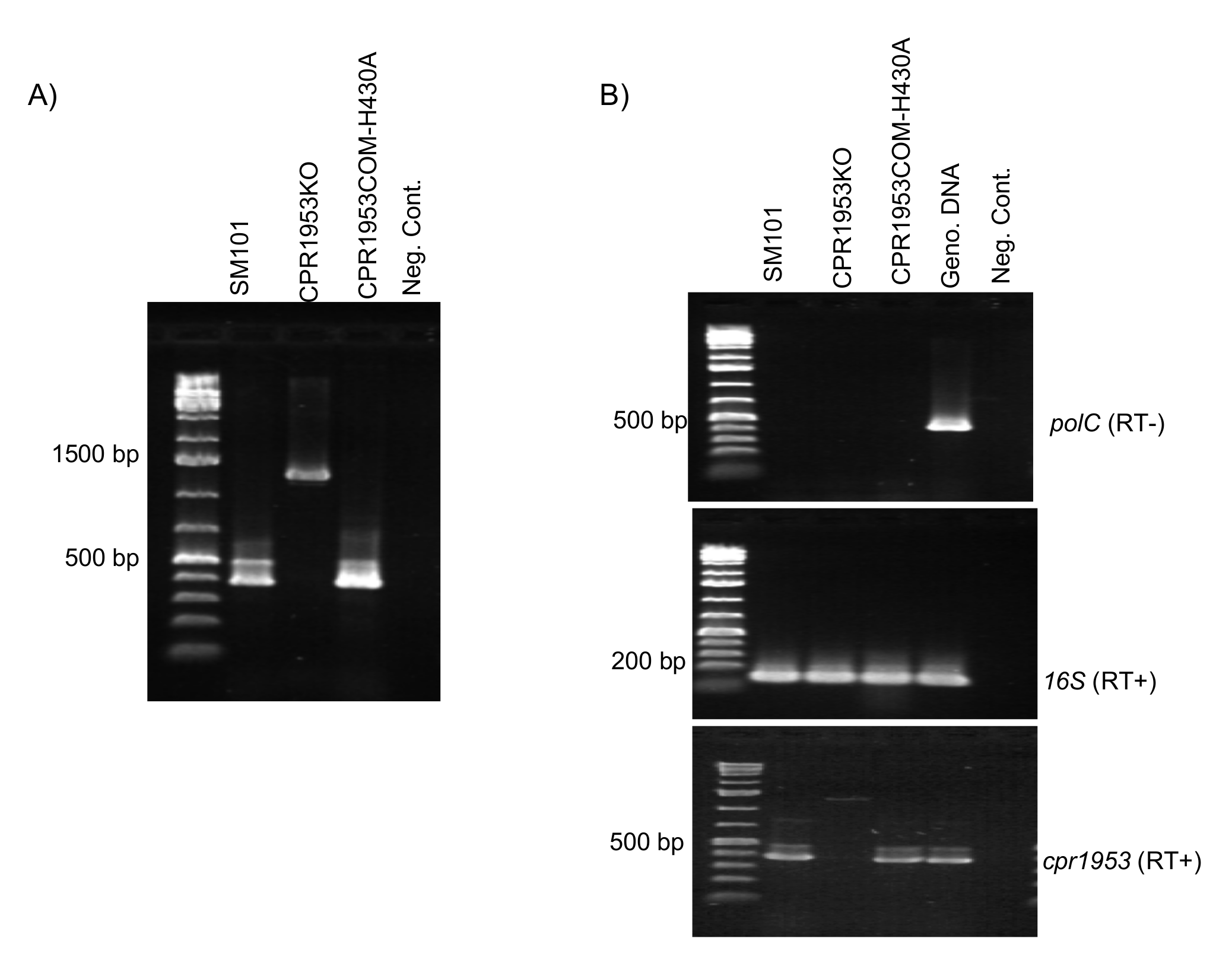

Supplement: S14 Fig — (A) PCR assay confirming construction of CPR1953COM-H430A complementing strain. Specific internal primers for cpr1953 amplified a larger PCR product using DNA from CPR1953KO (1265 bp) versus DNA from SM101 (365 bp), consistent with the insertion of a 900 bp intron into the cpr1953 gene of the mutant. The complemented strain amplified a 365 bp product when the same primers were used, consistent with the introduction of the complementing cpr1953 gene encoding an alanine substitution for the key functional His residue into CPR1953KO. (B) RNA was isolated from SM101, CPR1953KO, and CPR1953COM-H430A grown in MDS for 3 h at 37°C and the purity of each isolated RNA was shown by PCR, without reverse transcriptase, for the polC housekeeping gene (top panel). Genomic DNA or a sample lacking DNA template were used as positive and negative controls, respectively. (middle panel) RT-PCR analysis for the 16S housekeeping gene to demonstrate the quality of each prepared RNA. (lower panel) RT-PCR analysis for expression of the cpr1953 gene. (TIF) [file ppat.1011429.s014.tif]

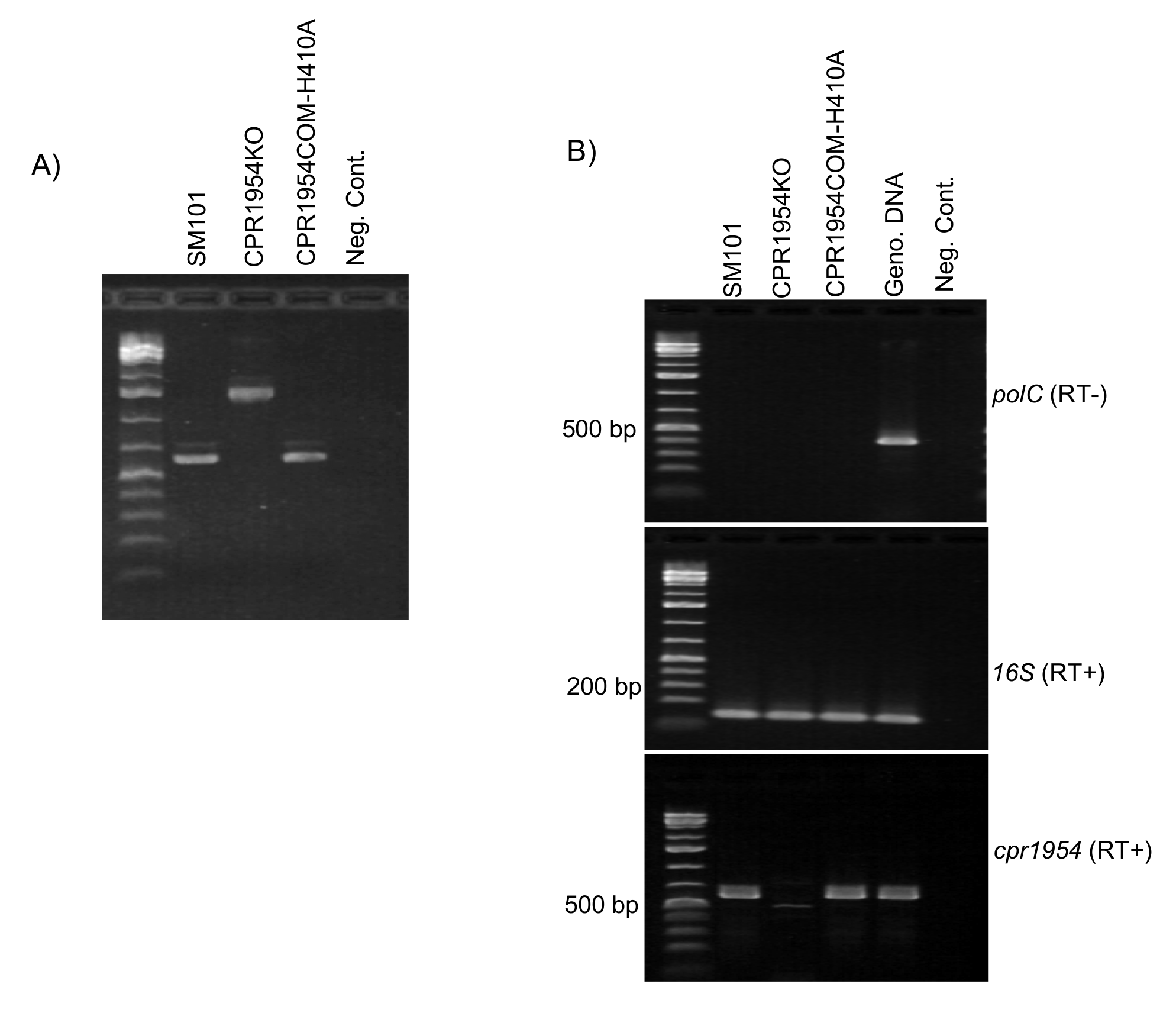

Supplement: S15 Fig — (A) PCR assay confirming construction of CPR1954COM-H410A complementing strain. Specific internal primers for the cpr1954 gene amplified a larger PCR product in CPR1954KO (1496 bp) versus SM101 (596 bp), consistent with the insertion of a 900 bp intron into the cpr1954 gene of the mutant. The complemented strain amplified a 596 bp product when the same primers were used, consistent with the introduction of the complementing cpr1954 gene encoding an alanine substitution for the key functional His residue into CPR1954KO. (B) RNA was isolated from SM101, CPR1954KO, and CPR1954COM-H410A grown in MDS for 3 h at 37°C and the purity of each isolated RNA was shown by PCR, without reverse transcriptase, for the polC housekeeping gene (top panel). Genomic DNA or a sample lacking DNA template were used as positive and negative controls, respectively. (middle panel) RT-PCR analysis for the 16S housekeeping gene to demonstrate the quality of each prepared RNA. (lower panel) RT-PCR analysis for expression of the cpr1954 gene. (TIF) [file ppat.1011429.s015.tif]
